# Therapeutic stress triggers tumor STAT1 acetylation to disarm immunotherapy

**DOI:** 10.1016/j.xcrm.2025.102448

**Published:** 2025-11-07

**Authors:** Po-Hsien Chiu, Kuan-Chen Lai, Hung-Ling Wang, Yao-Wen Chang, Wen-Chi Wu, Tien-Hua Chen, Yu-Shuen Tsai, Jie-Hong Song, Nai-Yun Sun, Gar-Yang Chau, Wen-Liang Fang, Ju-Pei Chen, Hung-Ming Wang, Huai-Cheng Huang, Meng-Che Hsieh, Chun-Hung Hua, Ming-Yu Lien, Yi-Fang Chang, Hui-Ching Wang, Chih-Yen Chien, Tai-Lin Huang, Chen-Chi Wang, Yi-Chun Liu, Jo-Pai Chen, Wei-Chen Lu, Ching-Yi Yiu, Chien-Liang Lin, Pei-Jen Lou, Pen-Yuan Chu, Shao-Chun Wang, Mien-Chie Hung, Muh-Hwa Yang

**Affiliations:** 1 Program in Molecular Medicine, National Yang Ming Chiao Tung University and Academia Sinica, Taipei 112304, Taiwan; 2 Institute of Clinical Medicine, National Yang Ming Chiao Tung University, Taipei 112304, Taiwan; 3 Cancer and Immunology Research Center, National Yang Ming Chiao Tung University, Taipei 112304, Taiwan; 4 Center for Molecular Medicine, China Medical University Hospital, Taichung 406040, Taiwan; 5 Research Center for Cancer Biology, China Medical University, Taichung 40402, Taiwan; 6 Cancer Biology and Precision Therapeutics Center, China Medical University, Taichung 40402, Taiwan; 7 Department of Oncology, Taipei Veterans General Hospital, Taipei 112201, Taiwan; 8 Division of General Surgery, Department of Surgery, Taipei Veterans General Hospital, Taipei 112201, Taiwan; 9 Division of Hematology-Oncology, Department of Internal Medicine, Chang Gung Memorial Hospital, Linkou, Taoyuan 333423, Taiwan; 10 Department of Oncology, National Taiwan University Hospital and College of Medicine, Taipei 100225, Taiwan; 11 Department of Hematology and Oncology, E-Da Cancer Hospital, Kaohsiung 824005, Taiwan; 12 Department of Otorhinolaryngology, China Medical University Hospital, Taichung 404327, Taiwan; 13 Division of Hematology and Oncology, Department of Internal Medicine, China Medical University Hospital, Taichung 404327, Taiwan; 14 Division of Hematology and Oncology, Department of Internal Medicine, MacKay Memorial Hospital, Taipei 104217, Taiwan; 15 Division of Hematology and Oncology, Department of Internal Medicine, MacKay Memorial Hospital, Taitung 950408, Taiwan; 16 Division of Hematology and Oncology, Department of Internal Medicine, Kaohsiung Medical University Hospital, Kaohsiung 807377, Taiwan; 17 Department of Otolaryngology, Kaohsiung Chang Gung Memorial Hospital, Doctoral Program of Clinical and Experimental Medicine, National Sun Yat-sen University, Kaohsiung 833401, Taiwan; 18 Department of Hematology-Oncology, Kaohsiung Chang Gung Memorial Hospital, Kaohsiung 833401, Taiwan; 19 Department of Otolaryngology-Head and Neck Surgery, Taichung Veterans General Hospital, Taichung 407219, Taiwan; 20 Department of Radiation Oncology, Taichung Veterans General Hospital, Taichung 407219, Taiwan; 21 Department of Oncology, National Taiwan University Hospital Yunlin Branch, Yunlin 632007, Taiwan; 22 Department of Otolaryngology, Yumin Medical Corporation Yumin Hospital, Caotun, Nantou 542007, Taiwan; 23 Division of Hematology-Oncology, Department of Internal Medicine, Chi Mei Medical Center, Liouying, Tainan 736402, Taiwan; 24 Department of Otolaryngology, National Taiwan University Hospital and National Taiwan University College of Medicine, Taipei 100225, Taiwan; 25 Department of Otolaryngology, Taipei Veterans General Hospital, Taipei 112201, Taiwan; 26 Graduate Institute of Biomedical Sciences, Institute of Biochemistry and Molecular Biology, China Medical University, Taichung 40402, Taiwan; 27 Department of Research and Education, Taipei City Hospital, Taipei 103212, Taiwan

**Keywords:** inteferon gamma signaling, immune checkpoint blockade, STAT1, acetylation

## Abstract

Sequential cancer therapy presents a critical challenge, as the impact of prior treatments on immunotherapy remains unclear. Here, we demonstrate that therapeutic stress from prolonged cetuximab exposure induces tumor-intrinsic resistance to immune checkpoint blockade (ICB) in head and neck squamous cell carcinoma (HNSCC). In a multicenter analysis, extended cetuximab treatment correlates with poor ICB response and survival. Mechanistically, chronic therapeutic stress provokes an initial inflammatory response that transitions into immune resistance. A previously unknown post-translational modification, STAT1 lysine 637 acetylation, serves as the molecular switch driving this process. Triggered by treatment-induced tumor necrosis factor alpha (TNF-α), this acetylation impairs STAT1 dimerization and transcriptional activity, while treatment-induced interferon (IFN)-β promotes STAT1 phosphorylation at tyrosine 701 and subsequent degradation. These modifications disrupt tumor IFN-γ responsiveness. Importantly, STAT1 acetylation in pre-treatment tumor samples predicts ICB efficacy, underscoring its potential as a clinically relevant biomarker for guiding immunotherapy decisions.

## Highlights

• Cetuximab resistance impairs tumor IFN-γ response and reduces ICB efficacy
• STAT1 Lys637 acetylation blocks dimerization and suppresses transcription
• IFN-β triggers STAT1 degradation and TNF-α drives Lys637 acetylation
• High STAT1 acetylation predicts poor survival and worse ICB response in HNSCC

## Graphical abstract

**Figure:**
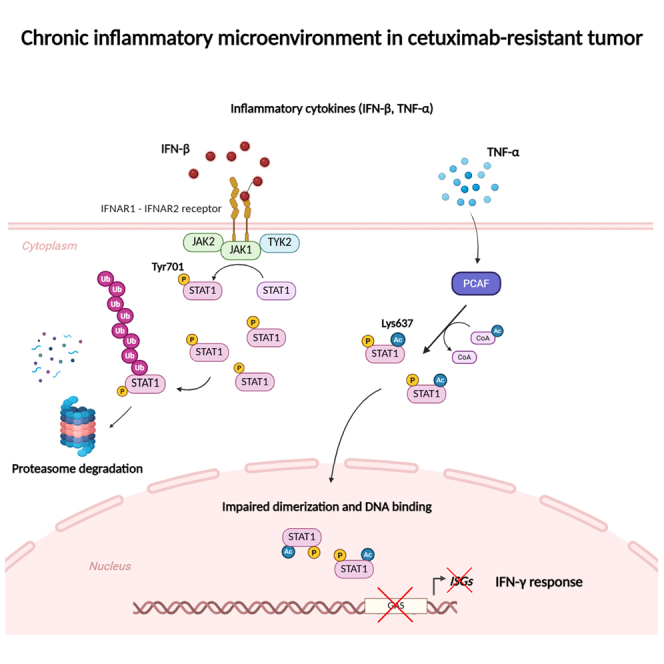

## Teaser

Chiu et al. show that prolonged cetuximab treatment in head and neck cancer triggers STAT1 Lys637 acetylation, disrupts interferon (IFN)-γ signaling, and drives resistance to immune checkpoint blockade. This identifies a clinically relevant biomarker that links prior therapy to immunotherapy response and suggests strategies for treatment stratification.

## Introduction

The expanding arsenal of cancer therapeutics has created unprecedented complexity in treatment scheduling, where the order of different modalities profoundly influences patient outcomes. While immune checkpoint blockade (ICB) has revolutionized cancer treatment,^1^^,^^2^ its efficacy remains suboptimal with only 20% response rate across most cancer types.^3^ The impact of treatment sequence on therapeutic efficacy is emerging as a critical question in head and neck squamous cell carcinoma (HNSCC), where both anti-EGFR therapy and immunotherapy represent major therapeutic advances.^2^^,^^4^^,^^5^^,^^6^^,^^7^ Subgroup analyses from pivotal anti-PD1 trials have indicated that HNSCC patients previously treated with the anti-EGFR antibody cetuximab might experience reduced response to subsequent immunotherapy.^4^^,^^8^ These observations from clinical trials, although not powered for definitive conclusions, align with findings in other cancers, such as melanoma where randomized trials have demonstrated that BRAF inhibitor treatment before immunotherapy yields inferior outcomes.^9^ While real-world evidence examining the impact of prior targeted therapy on ICB efficacy in HNSCC remains limited, understanding the molecular basis of treatment sequence effects has become increasingly critical.

Recent evidence reveals that cancer cells undergo dynamic adaptations along a resistance continuum during therapy. These adaptations, rather than following discrete resistant states, represent a progressive evolution of cellular programs that fundamentally alter treatment response.^10^ During this adaptation process, cancer cells exhibit dynamic changes in transcriptional networks, particularly in IFN and inflammatory signaling pathways.^10^^,^^11^ These findings suggest that therapeutic pressure induces systematic cellular reprogramming that extends beyond the primary drug target, potentially affecting the efficacy of subsequent treatments. However, the mechanisms governing such adaptation-mediated cross-resistance, especially how prior targeted therapy might compromise immunotherapy response, remain largely undefined.

Given the clinical observations of treatment sequence effects in HNSCC and the emerging concept of therapy-induced adaptation, understanding EGFR pathway modulation becomes particularly relevant. The relationship between EGFR signaling and tumor immunity illustrates this complexity. EGFR activation promotes immune evasion through multiple mechanisms, including downregulation of antigen presentation,^12^ increased checkpoint ligand expression,^13^ secretion of immune inhibitory factors,^14^ and altered immune metabolic profiles.^15^ Paradoxically, inhibiting EGFR does not restore antitumor immunity but can enhance immunosuppression, as evidenced by increased intratumoral regulatory T cells after treatment.^16^ Moreover, established mechanisms of EGFR-targeted therapy resistance^17^^,^^18^^,^^19^^,^^20^^,^^21^^,^^22^ and ICB resistance^23^ appear largely distinct, suggesting undiscovered adaptations that bridge these therapeutic modalities.

In this study, we show that therapeutic stress induces a previously unrecognized STAT1 modification that weakens tumor immune response, linking prior targeted therapy to immunotherapy resistance and highlighting implications for treatment scheduling and patient stratification.

## Results

### Cetuximab resistance correlates with decreased efficacy of immune checkpoint blockades and reduced tumor-infiltrative T cells

**Figure 1:**
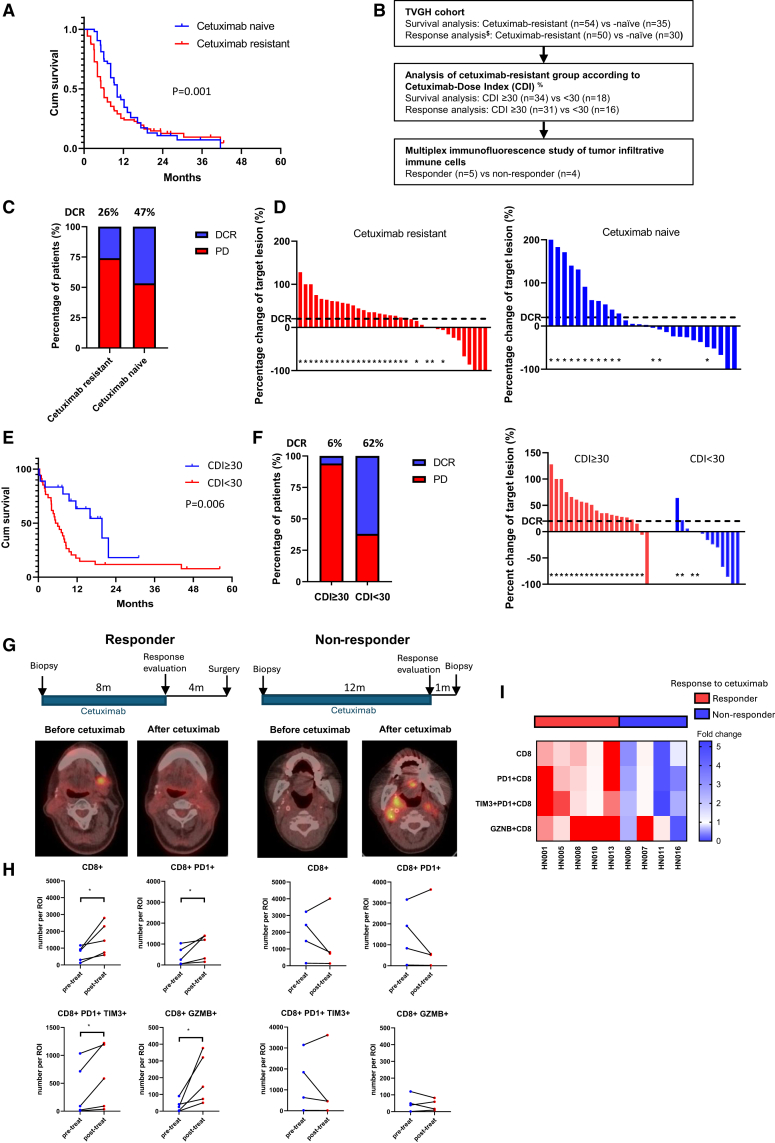
Correlation between cetuximab resistance and decreased ICB efficacy alongside reduced tumor-infiltrating immune cells (A) Kaplan-Meier survival curves and log rank test for overall survival from the initiation of immunotherapy in cetuximab-resistant (*n* = 104) vs. cetuximab-naive (*n* = 60) patients from the THNS registry. (B) Flowchart depicting the clinical efficacy evaluation and multiplex immunofluorescence study of tumor-infiltrating immune cells in the TVGH cohort. (C) The proportion of patients who achieved disease control with immunotherapy in cetuximab-resistant (*n* = 50) vs. cetuximab-naive (*n* = 30) patients in the TVGH cohort. Blue indicates disease control rate (DCR), and red indicates progressive disease (PD). (D) Waterfall plots showing the percentage change in measurable target lesions in cetuximab-resistant (left, *n* = 36) vs. cetuximab-naive (right, *n* = 28) patients in the TVGH cohort. Asterisks denote PD as the best overall response. (E) Kaplan-Meier survival curve and log rank test for overall survival in patients with a cetuximab density index (CDI) > 30 (*n* = 34) vs. CDI <30 (*n* = 18) from the TVGH cohort. CDI was calculated as follows: (accumulated cetuximab dose [mg])/(interval of cetuximab treatment [months] × interval between the end of cetuximab and the beginning of immunotherapy [months]). (F) Left: Proportion of patients achieving disease control with immunotherapy in CDI >30 (*n* = 31) vs. CDI <30 (*n* = 16) patients in the TVGH cohort. Blue represents DCR, and red represents PD. Right: Waterfall plots showing the percentage change in measurable target lesions in patients with CDI >30 (*n* = 20) vs. CDI <30 (*n* = 13) from the TVGH cohort. Asterisks indicate PD as the best overall response. (G) Representative cases of tumor-infiltrating immune cells in cetuximab-responsive (left) and cetuximab-resistant (right) samples. Schematic of the clinical course of patients. (Bottom) Positron emission tomography imaging before and after cetuximab treatment. (H) Representative cases of tumor-infiltrating immune cells in cetuximab-responsive (left) and cetuximab-resistant (right) samples. Fold change in CD8^+^, CD8^+^PD1^+^, CD8^+^PD1^+^TIM3^+^, and CD8^+^GZMB^+^ cell numbers in the regions of interest (ROIs, 924 × 693 μm^2^) of pre-cetuximab and post-cetuximab biopsy specimens. (I) Heatmap illustrating the fold change in tumor-infiltrating immune cell numbers between pre- and post-cetuximab biopsy specimens, categorized as cetuximab responders (*n* = 5) and non-responders (*n* = 4). See also Figure S1. Tables S1 and S2.

To comprehensively evaluate how prior cetuximab exposure affects immunotherapy outcomes, we analyzed two independent cohorts of HNSCC patients. In the Taiwan Head and Neck Society (THNS) registry cohort (characteristics in Table S1A), patients who were cetuximab naive showed significantly longer overall survival (OS) under ICB treatment compared to those with prior cetuximab exposure (*p* = 0.001; Figure 1A). Multivariate analysis confirmed that the absence of prior cetuximab exposure represents an independent prognostic factor for recurrent/metastatic HNSCC (Table S1B). We validated these findings in an independent cohort from Taipei Veterans General Hospital (TVGH; patient flow in Figure 1B and patient characteristics in Table S2). The OS and progression-free survival (PFS) showed similar trends to the THNS cohort (*p* = 0.07 for OS, *p* = 0.11 for PFS; Figure S1A), and the disease control rate was significantly higher in cetuximab-naive patients (Figures 1C and 1D). To precisely quantify the impact of prior cetuximab exposure, we developed a cetuximab density index (CDI), which considers both the cumulative dose and the time interval between cetuximab and ICB treatment (see STAR Methods for details). This analysis revealed that patients with lower cetuximab exposure (CDI <30) showed both longer survival (Figure 1E) and higher disease control rates (Figure 1F) during subsequent ICB therapy. Moreover, extending the interval between cetuximab and ICB treatment (≥6 months) is associated with improved survival and treatment response (Figures S1B and S1C).

We further analyzed tumor immune infiltration in paired patient samples collected before and after cetuximab treatment (*n* = 9, with 5 responders and 4 non-responders). Multiplex immunofluorescence analysis revealed distinct patterns of CD8^+^ T cell dynamics: non-responders have a higher overall number of tumor infiltrating lymphocytes (TILs) compared to responders, both before and after cetuximab treatment. However, this increase in non-responders is primarily due to exhausted CD8^+^PD1^+^TIM3^+^ T cells. In contrast, the number of effector CD8^+^GZMB^+^ T cells is very low in non-responders before treatment and remains very low after treatment, typically fewer than 100 cells per region of interest. In responders, CD8^+^GZMB^+^ T cells increase markedly following treatment (Figures 1G–1I). These findings suggest that cetuximab resistance not only compromises subsequent immunotherapy efficacy but also fundamentally alters the tumor immune microenvironment through modulation of T cell infiltration patterns.

### Dynamic alteration of pro-inflammatory response in HNSCC cells with prolonged cetuximab treatment

**Figure 2:**
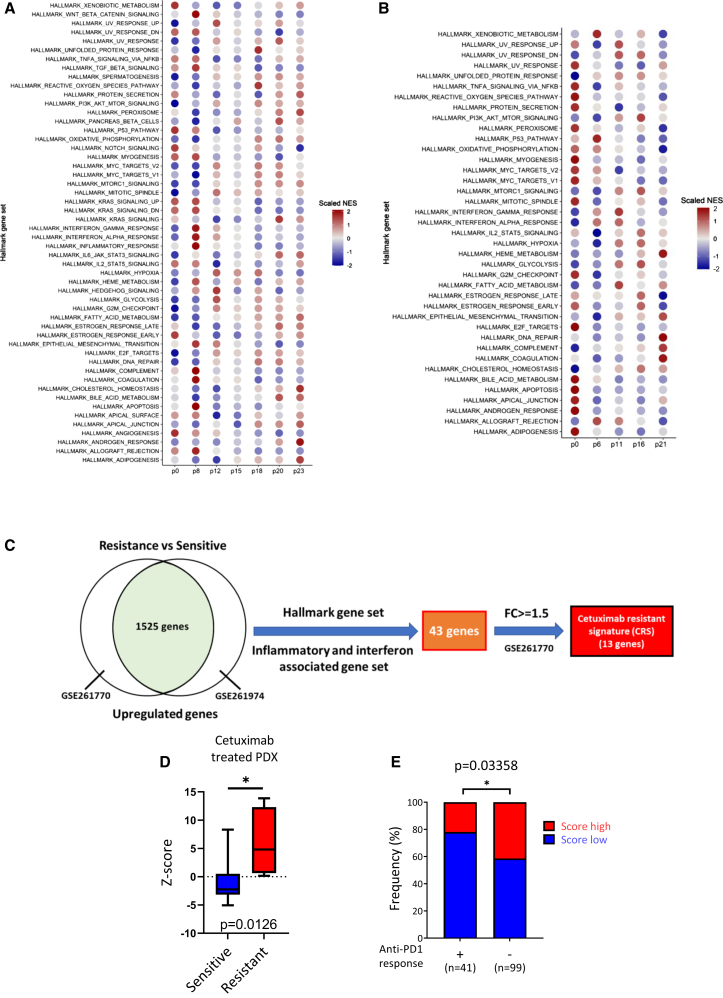
Proteogenomic analysis of HNSCC cells following prolonged cetuximab exposure and correlation of cetuximab-resistance signature with ICB resistance (A) RNA sequencing of OECM-1 cells treated with cetuximab (500 μg/mL) across the indicated passages, followed by single-sample GSEA of hallmark gene sets. Red, upregulated gene sets; blue, downregulated gene sets (*N* = 3). (B) Mass spectrometric analysis of OECM-1 cells treated with cetuximab (500 μg/mL) at different passages highlighted up- and downregulated proteins identified from the mass spectrometry results (*N* = 3). (C) Schematic representation of the identification of cetuximab resistance signature (CRS). A total 1,525 upregulated genes identified from GEO: GSE261700 (OECM-1-Ctx^R^/OECM-1-WT and CAL27-Ctx^R^/CAL-27-WT) and GEO: GSE261974 (patient pre- and post-cetuximab treatment) were used to intersect with the inflammatory and interferon-associated gene within hallmark gene set. Thirteen genes were selected with a fold change ≥1.5. (D) Validation of CRS in a head and neck cancer patient-derived xenograft dataset, comparing cetuximab-sensitive and -resistant tissues. The data were obtained from the GEO database (GSE183881). Sensitive tissues (*n* = 12) and resistant tissues (*n* = 4). Boxplots display the minimum, median, and maximum values. Statistical analysis was performed using unpaired Student’s *t* test. ∗*p* < 0.05. (E) Frequency of anti-PD1 response based on the cetuximab resistance signature (CRS) status (high vs. low) across four cohorts (GEO: GSE179730, GSE78220, GSE159067, and GSE195832). Responders (*n* = 41) and non-responders (*n* = 99) are shown. Statistical significance was assessed using a two-sided Fisher’s exact test. ∗*p* < 0.05. See also Figure S2 and Tables S3, S4, S5, S6, S7, and S8.

To understand how prolonged cetuximab treatment affects tumor cell programs and leads to ICB resistance, we conducted a systematic analysis of treatment-induced cellular adaptations. We screened a panel of HNSCC cell lines for cetuximab response (Figure S2A) and selected OECM-1 cells for detailed longitudinal analysis during resistance development. OECM-1 cells were treated with cetuximab (500 μg/mL) for 30 passages over more than 2 months, and samples from different passages were collected for RNA sequencing (Table S3) and proteomic analysis (Table S4). Time-course RNA sequencing revealed a dynamic pattern of cellular adaptation: single-sample gene set enrichment analysis (GSEA) showed strong enhancement of pro-inflammatory signatures, including IFN-γ and IFN-α, during early cetuximab exposure (≤8 passages), which diminished in later passages (>8 passages). Epithelial-mesenchymal transition (EMT) signaling increased in mid-passages (8–12 passages), while metabolic reprogramming and DNA repair pathways were enriched in late passages (≥18 passages) (Figure 2A; Table S5). Gene Ontology analysis comparing p8 vs. p0 highlighted the enrichment of type I IFN signaling and related immune pathways in the early treatment phase (Figure S2B). Mass spectrometric analysis of proteomic changes confirmed the presence of these pathways, including the upregulation of IFN-stimulated genes (ISGs) and inflammatory response genes in early passages, followed by the emergence of EMT in middle passages and the enrichment of metabolism and DNA repair pathways in later passages (Figures 2B and S2C; Table S4).

To validate these findings, we established two cetuximab-resistant cell lines (OECM-1-Ctx^R^ and CAL-27-Ctx^R^) through continuous drug exposure (500 μg/mL for 30 passages over 2 months) (Figure S2D). These resistant lines, after confirming their phenotypes through viability assays and EGFR pathway analysis (Figures S2E and S2F), showed persistent upregulation of inflammatory and IFN-response genes (Figures S2G–S2I). Notably, as upregulation of IFN response and ISG resistance signature (ISG-RS) has been reported to associate with ICB resistance,^24^ we examined this correlation in our models. GSEA revealed that cetuximab resistance was highly correlated with ISG-RS. However, there is no significant association between the non-RS ISG (hallmark ISG gene set excluding ISG-RS genes) and resistance, supporting the selective enrichment of the ISG-RS in the context of resistance (Figure S2J). Analysis of patient samples before (*n* = 3) and after (*n* = 5) cetuximab treatment further validated these findings, showing significant upregulation of inflammatory and IFN-associated responses in post-treatment samples (Figure S2K; Table S6).

To develop a cetuximab resistance signature (CRS), we integrated our cellular and clinical findings by identifying overlapping upregulated genes between the two resistant sublines (Table S7) and post-cetuximab patient samples (Table S6). We focused on inflammatory-related hallmark genes and IFN-related genes that showed ≥1.5-fold upregulation in post-cetuximab samples (Figure 2C). The complete CRS gene list is provided in Table S8. The expression of these 13 genes comprising the CRS were validated in two cetuximab-resistant HNSCC cell lines compared to their corresponding parental wild-type cell lines (Figure S2L). The CRS was further validated using sequencing data from cetuximab-resistant patient-derived xenografts (PDXs) (*n* = 4 resistant PDXs, *n* = 12 sensitive PDXs; Figure 2D). The CRS correlated with poor ICB response in an independent clinical cohort (*n* = 140, with 99 non-responders and 41 responders) (Figure 2E), demonstrating its potential to predict ICB resistance. Taken together, these results indicate that therapeutic stress induced by cetuximab elicits a dynamic inflammatory response in tumor cells, which evolves during the development of resistance. The correlation between our CRS and ICB resistance patterns highlights the link between targeted therapy-induced cellular adaptations and immunotherapy resistance.

### STAT1 downregulation drives impaired interferon-γ response in cetuximab-resistant HNSCC

**Figure 3:**
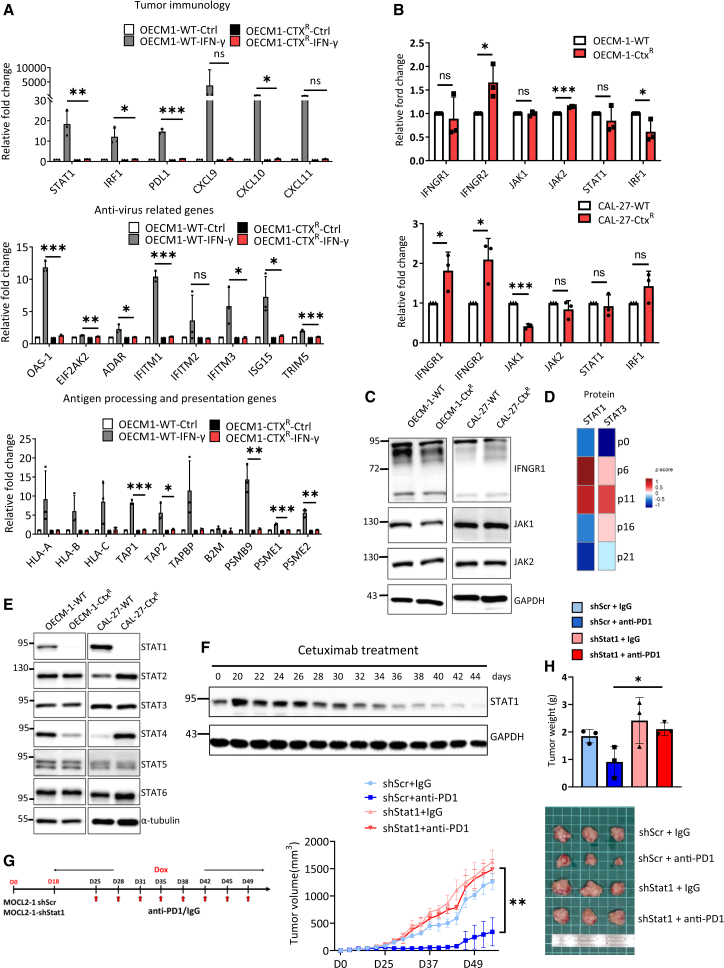
Impaired IFN-γ response and reduced STAT1 protein in cetuximab-resistant HNSCC (A) RT-qPCR of IFN-γ response-associated gene expression, including tumor immunology-related genes (upper), antiviral-related genes (middle), and antigen processing and presentation genes (lower) in OECM-1-WT and OECM-1-Ctx^R^ cells. *n* = 3 (each with two technical replicates). The cells were then treated with IFN-γ (100 ng/mL) for 24 h. Data are presented as mean ± SD. Statistical significance was determined using unpaired Student’s *t* test. ∗*p* < 0.05; ∗∗*p* < 0.01; ∗∗∗*p* < 0.001; ns, not significant. (B) RT-qPCR of IFN-γ signaling-associated components in OECM-1-WT/CAL-27-WT and OECM-1-Ctx^R^/CAL-27-Ctx^R^ cells. *n* = 3 (each with two technical replicates). Data are presented as mean ± SD. Statistical significance was determined using unpaired Student’s *t* test. ∗*p* < 0.05; ∗∗∗*p* < 0.001; ns, not significant. (C) Representative western blot analysis of IFN-γ signaling-related proteins in OECM-1-WT/OECM-1-Ctx^R^ and CAL-27-WT/CAL-27-Ctx^R^ cells. GAPDH was the loading control. The experiments were performed in triplicate. (D) Heatmap showing STAT1 and STAT3 protein levels from mass spectrometry in OECM-1 cells after cetuximab treatment (500 μg/mL) across different passages. (E) Representative western blot analysis of STAT family in OECM-1-WT/OECM-1-Ctx^R^ and CAL-27-WT/CAL-27-Ctx^R^ cells. α-tubulin was used as the loading control. The experiments were performed in triplicate. (F) Representative western blot analysis of STAT1 protein levels in OECM-1 cells across different passages of cetuximab treatment (500 μg/mL). GAPDH was used as a loading control. The experiments were performed in triplicate. (G) Left: Schematic of the mouse experiment. Murine oral squamous cell carcinoma MOC-L2-1 cells were transduced with a doxycycline (DOX)-inducible vector for the knockdown of Stat1 (shStat1) or a scramble control (shScr) and were then inoculated subcutaneously into C57BL/6 mice. Doxycycline administration was initiated on day 18 to induce vector expression in syngeneic tumors. Mice were treated with either isotype IgG or murine anti-PD1 (200 μg) for 8 doses at specified time points. Right: Tumor growth curves are presented as mean ± SD. *n* = 3 per group. Statistical significance was determined using unpaired Student’s *t* test. ∗∗*p* < 0.01. (H) Upper: Histogram showing weights of shScr and shStat1 MOC-L2-1 tumors. *n* = 3 per group. Statistical significance was determined using unpaired Student’s *t* test. ∗*p* < 0.05. Lower: Representative images of tumors. See also Figure S3.

The IFN-γ response in tumor cells is critical for the efficacy of ICB therapy, as it regulates the expression of genes involved in antigen presentation, cytokine production, and immune checkpoint ligands.^25^^,^^26^ In this study, we found that cetuximab resistance in HNSCC is linked to an early inflammatory signature and an initial IFN response that diminishes over time (Figure 2). We explored the impact of cetuximab resistance on the IFN-γ response in HNSCC. We found that the induction of IFN-γ response genes, including those associated with tumor immunology, antiviral activity, and antigen processing and presentation, was abolished in cetuximab-resistant cells at both the mRNA (Figure 3A) and protein levels (Figure S3A). This suggests an intrinsic inactivation of the IFN-γ pathway within tumor cells during cetuximab treatment. Analysis of the IFN-γ axis showed no significant differences in *IFNGR1*, *JAK1/2*, *STAT1*, or *IRF1* mRNA levels between parental and cetuximab-resistant cells (Figure 3B). The protein levels of IFNGR1, JAK1, and JAK2 also remained unchanged in the resistant sublines (Figure 3C). As mutations in IFN-γ signaling genes are involved in ICB resistance,^27^ we performed whole-exome sequencing for the parental and resistant sublines. The results showed no genetic alterations in *IFNGR1/2*, *JAK1/2*, or *STAT1* in the cetuximab-resistant sublines (Figure S3B). Additionally, no consistent alteration in the expression of other checkpoint ligands was noted (Figure S3C), ruling out the possibility of induction of alternative checkpoint ligands as a mechanism for ICB resistance in cetuximab-resistant HNSCC cells.

Given that STAT family proteins are crucial transcription factors regulating IFN responses^28^^,^^29^ and essential for ICB efficacy,^30^ we examined the levels of STAT proteins in cetuximab-resistant HNSCC cells. Proteomic analysis of STAT family proteins revealed that among the detectable STAT proteins, both STAT1 and STAT3 were initially upregulated during early cetuximab exposure, but this induction was followed by a later decrease (Figure 3D). The expression pattern of STAT family proteins was further validated in two resistant sublines. While the expression of other STAT proteins was not consistently altered, STAT1 was significantly downregulated in the resistant sublines compared to the parental cells (Figure 3E). Consistent with the proteomic analysis, western blot analysis confirmed that STAT1 protein level was initially upregulated and then downregulated during prolonged cetuximab treatment (Figure 3F). In contrast, STAT3 protein level remained unchanged during prolonged cetuximab treatment (Figure S3D). To further validate the role of STAT1, we employed inducible knockdown of Stat1 in a syngeneic oral SCC (OSCC) murine model.^31^ The reduced IFN-γ response following *Stat1* knockdown in the murine OSCC cell line was confirmed *in vitro* before inoculation into mice (Figure S3E). Importantly, the induction of Stat1 knockdown in murine OSCC cells led to sustained tumor growth despite anti-PD1 administration, whereas significant tumor shrinkage was observed in the control group (Figures 3G and 3H). In summary, cetuximab resistance results in an impaired IFN-γ response through the downregulation of STAT1.

### Chronic STAT1 activation leads to its degradation in cetuximab-resistant HNSCC

**Figure 4:**
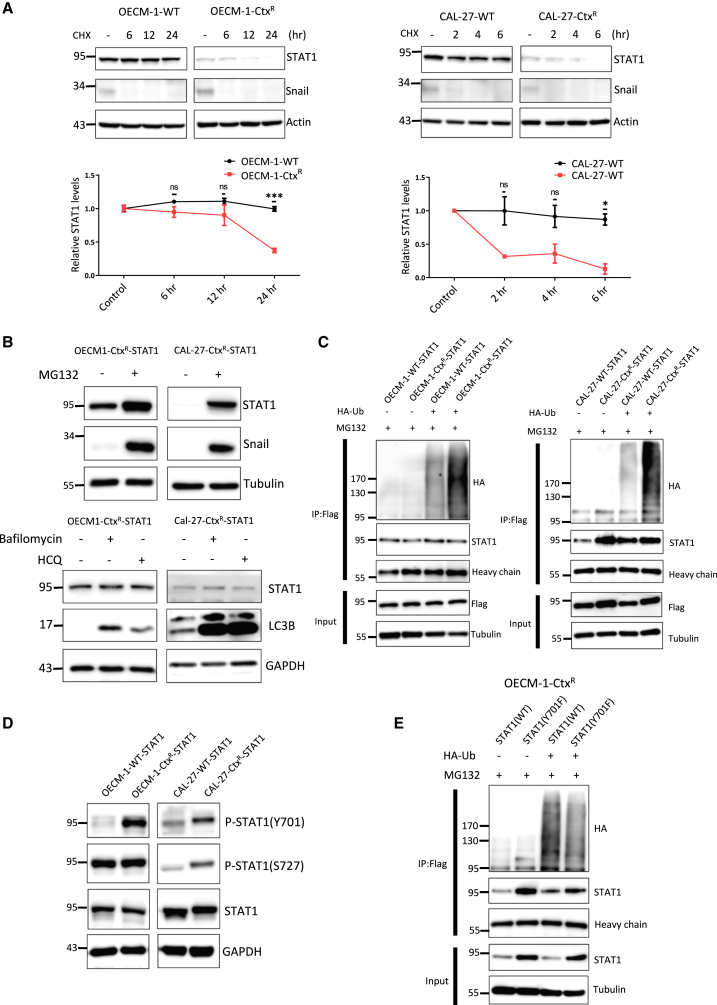
Tyrosine 701 phosphorylation promotes STAT1 degradation in cetuximab-resistant HNSCC (A) Upper: Representative western blot analysis of STAT1 protein levels in OECM-1-WT/OECM-1-Ctx^R^ (left) and CAL-27-WT/CAL-27-Ctx^R^ (right) cells following treatment with cycloheximide (20 μg/mL) for the indicated times. β-actin was the loading control. Lower: Quantification of STAT1 protein levels. Data are presented as the mean ± SD. *n* = 3 per group. Statistical significance was determined using unpaired Student’s *t* test. ∗*p* < 0.05; ∗∗∗*p* < 0.001; ns, not significant. (B) Upper: Representative western blot analysis of STAT1 protein levels in OECM-1-Ctx^R^ (left) and CAL-27-Ctx^R^ (right) cells transfected with STAT1 (OECM-1-Ctx^R^-STAT1 and CAL-27-Ctx^R^-STAT1) and treated with proteasome inhibitor (MG132, 20 μM) for 18 h. Snail was the positive control for proteasomal degradation. Lower: Representative western blot analysis of STAT1 protein levels in OECM-1-Ctx^R^ (left) and CAL-27-Ctx^R^ (right) cells transfected with STAT1 (OECM-1-Ctx^R^-STAT1 and CAL-27-Ctx^R^-STAT1) and treated with lysosomal inhibitor (bafilomycin A1, 100 nM) or autophagic degradation inhibitor (hydroxychloroquine [HCQ], 20 μM). LC3B is a marker for monitoring autophagy. GAPDH was the loading control. The experiments were performed in triplicate. (C) Representative immunoprecipitation and western blot analyses of polyubiquitinated STAT1 in OECM-1-WT/OECM-1-Ctx^R^ (left) and CAL-27-WT/CAL-27-Ctx^R^ (right) cells transfected with STAT1. The cells were treated with MG132 (20 μM) for 6 h to inhibit proteasome degradation. The experiments were performed in triplicate. (D) Representative western blot analysis of total STAT1, Tyr701-phosphorylated STAT1, and Ser727-phosphorylated STAT1 in OECM-1-WT/OECM-1-Ctx^R^ (left) and CAL-27-WT/CAL-27-Ctx^R^ (right) cells transfected with STAT1. The cells were treated with MG132 (10 μM) for 16 h to inhibit proteasome degradation. GAPDH was the loading control. The experiments were performed in triplicate. (E) Representative immunoprecipitation and western blot analyses of polyubiquitinated STAT1 in OECM-1-Ctx^R^ cells transfected with wild-type (WT) or Tyr701-unphosphorylatable mutant (Y701F) STAT1. Cells were treated with MG132 (10 μM) for 6 h to inhibit proteasomal degradation. The experiments were performed in triplicate. See also Figure S4.

Based on the finding that cetuximab resistance blunts the IFN-γ response by reducing STAT1 protein levels without genetic aberrations or transcriptional repression, we investigated the mechanism of reduced STAT1 protein in cetuximab-resistant HNSCC. A pulse-chase assay confirmed reduced STAT1 protein stability in both cetuximab-resistant sublines compared to their parental cells (Figure 4A). To investigate the mechanism of STAT1 protein degradation, we reconstituted STAT1 in resistant sublines. Inhibition of proteasomal degradation successfully rescued STAT1 levels, whereas inhibition of lysosomal or autophagic degradation did not (Figure 4B). We explored the post-translational modifications of STAT1 that influence its stability in resistant cells. Polyubiquitination of STAT1 was observed in resistant cells reconstituted with STAT1 (Figure 4C). Mass spectrometric analysis revealed phosphorylation of tyrosine 701 (Tyr701) and serine 727 (Ser727) of STAT1 in resistant cells (Figure S4A). Phosphorylation at both Tyr701 and Ser727 leads to the full activation of STAT1,^32^ while Tyr701 phosphorylation negatively feeds back to downregulate STAT1 through polyubiquitination and degradation to prevent overactivation.^33^ We found that Tyr701 phosphorylation of STAT1 was consistently enriched in two cetuximab-resistant sublines compared to the parental cells, whereas Ser727 phosphorylation was enriched in CAL-27-Ctx^R^ but not OECM-1-Ctx^R^ (Figure 4D). Reconstitution with a Tyr701-unphosphorylatable mutant [STAT1(Y701F)] reduced STAT1 polyubiquitination in 293T cells (Figure S4B) and cetuximab-resistant cells (Figure 4E), while the Ser727-unphosphorylatable mutant [STAT1(S727A)] did not affect polyubiquitination (Figure S4C). The dual-unphosphorylatable mutant [STAT1(Y701F/S727A)] abrogated STAT1 polyubiquitination (Figure S4D). To identify the potential E3 ligase responsible for STAT1 Tyr701 phosphorylation in cetuximab-resistant cells, we knocked down previously reported STAT1-targeting E3 ligases, including *SMURF1* (encoding Smurf1) and *PDLIM2* (encoding SLIM).^34^^,^^35^ However, silencing either gene failed to restore STAT1 protein levels in resistant cells (Figures S4E and S4F). Taken together, these results indicate that in cetuximab-resistant HNSCC cells, chronic exposure to cetuximab leads to an inflammatory signature, resulting in the chronic activation of STAT1 and its subsequent degradation through polyubiquitination. The specific E3 ligase mediating STAT1 degradation in this context remains to be identified.

### STAT1 lysine 637 acetylation functions as a molecular switch to disable IFN-γ response

**Figure 5:**
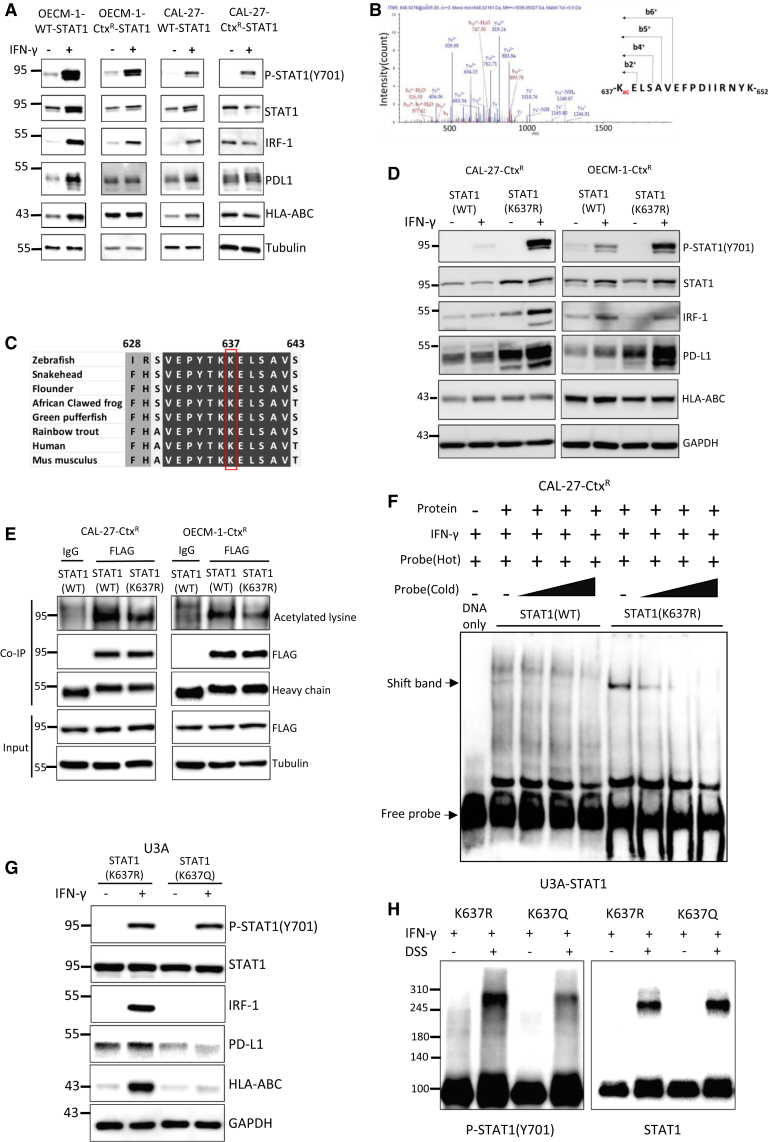
Reduced transcriptional activity of STAT1 in cetuximab-resistant HNSCC via Lys637 acetylation (A) Representative western blot analysis of the indicated proteins in OECM-1-WT/OECM-1-CtxR (left) and CAL-27-WT/CAL-27-Ctx^R^ (right) cells transfected with STAT1 and treated with or without IFN-γ (100 ng/mL) for 24 h. α-tubulin was the loading control. The experiments were performed in triplicate. (B) Mass spectrometric analysis of CAL-27-Ctx^R^ cells, identifying acetylation at Lys637 of STAT1. (C) Sequence alignment showing the conservation of STAT1 Lys637 across various species. (D) Representative western blot analysis of CAL-27-Ctx^R^ and OECM-1-Ctx^R^ cells transfected with wild-type or unacetylatable mutant STAT1(K637R), treated with or without IFN-γ (100 ng/mL) for 24 h. GAPDH was the loading control. The experiments were performed in triplicate. (E) Representative co-immunoprecipitation and western blot analyses detecting lysine-acetylated STAT1 in CAL-27-Ctx^R^ and OECM-1-Ctx^R^ cells transfected with wild-type STAT1 or STAT1(K637R). The cells were treated with MG132 (10 μM) for 16 h. The experiments were performed in triplicate. (F) Representative electrophoretic mobility shift assay assesses the DNA binding of wild-type STAT1 or STAT1(K637R) in CAL-27-Ctx^R^ cells. The cells were transfected with the corresponding vectors, treated with MG132 (10 μM, 16 h) and IFN-γ (100 ng/mL, 30 min). (G) Representative western blot analysis of the indicated proteins in U3A cells transfected with STAT1(K637R) or STAT1(K637Q) mutants and treated with IFN-γ (100 ng/mL) for 24 h. GAPDH was a loading control. The experiments were performed in triplicate. (H) Representative blot detecting dimerized STAT1 and Tyr701-phosphorylated STAT1 in U3A cells transfected with STAT1(K637R) or STAT1(K637Q) mutants treated with IFN-γ (100 ng/mL) with or without disuccinimidyl suberate (DSS) (2.5 μM) for 10 min. The experiments were performed in triplicate. See also Figure S5.

To dissect the molecular basis of IFN-γ resistance, we attempted to rescue the phenotype by reconstituting wild-type STAT1 in resistant cells. Unexpectedly, despite successful restoration of STAT1 protein levels, the IFN-γ response remained significantly impaired in both resistant sublines (Figures 5A, S5A, and S5B). This observation suggested that STAT1 protein downregulation alone could not fully explain the resistant phenotype, prompting us to investigate additional mechanisms. We first examined whether altered subcellular localization might explain the persistent dysfunction. Immunofluorescence analysis and nuclear-cytoplasmic fractionation assay revealed comparable nuclear accumulation of STAT1 upon IFN-γ stimulation in both parental and cetuximab-resistant cells (Figures S5C and S5D), indicating that defective nuclear translocation was not responsible for the impaired IFN-γ response.

We next investigated whether additional post-translational modifications might regulate STAT1 activity in resistant cells. Through unbiased mass spectrometric analysis, we identified a previously unrecognized acetylation site at lysine 637 (Lys637) of STAT1 (Figure 5B). This residue is particularly significant as it resides in the SH2 domain of STAT1 and shows high conservation across different species (Figure 5C). Given that the SH2 domain is essential for binding Tyr701 phosphorylation from the opposite STAT1 protein to form a functional homodimer and enable transcription activity,^36^ we hypothesized that acetylation at Lys637 might affect STAT1 transcriptional activity and compromise IFN-γ response in resistant cells. Supporting this hypothesis, reconstitution of an unacetylatable STAT1 mutant [STAT1(K637R)] in both resistant sublines restored IFN-γ response (Figures 5D and S5E). This functional rescue occurred without affecting protein stability, as demonstrated by comparable degradation kinetics between wild-type and mutant STAT1 (Figure S5F). Immunoprecipitation experiment confirmed reduced total acetylated lysine levels in STAT1 in resistant cells reconstituted with STAT1(K637R) compared to wild-type STAT1 (Figure 5E). We generated an antibody specifically recognizing STAT1 Lys637 acetylation for subsequent experiments. To validate its specificity, we performed western blot analysis in STAT1-null U3A cells and in cetuximab-resistant cells transfected with STAT1. No detectable acetylation signal was observed in U3A cells, whereas substantial signals were detected in the STAT1-transfected resistant cell lines, confirming the specificity of the antibody (Figure S5G). Moreover, increased STAT1 acetylation was observed in CAL-27-Ctx^R^ cells expressing wild-type STAT1 compared to parental cells. Transfection with the STAT1(K637R) mutant markedly diminished the Lys637 acetylation signal (Figure S5H).

To further evaluate the functional impact of Lys637 acetylation on STAT1 activity, we performed electrophoretic mobility shift assay, which revealed enhanced DNA-binding ability of the STAT1(K637R) mutant compared to wild-type STAT1 (Figure 5F). Consistent with this finding, reporter assays showed stronger response to IFN-γ in U3A cells expressing STAT1(K637R) vs. an acetylation-mimicking STAT1(K637Q) mutant (Figure S5I). Reconstitution of an acetylation-mimicking STAT1(K637Q) mutant in STAT1-null U3A cells failed to restore IFN-γ response, while the STAT1(K637R) mutant successfully rescued the phenotype (Figures 5G and S5J). The molecular mechanism underlying these functional differences became clear when we examined STAT1 dimerization. Upon IFN-γ stimulation, cells expressing STAT1(K637Q) showed significantly reduced levels of dimeric Tyr701-phosphorylated STAT1 compared to cells expressing STAT1(K637R) (Figure 5H). We further investigated the interplay between the two major post-translational modifications of STAT1 in cetuximab-resistant cells. Expression of either wild-type STAT1 or the acetylation-deficient mutant STAT1(K637R) did not affect Tyr701 phosphorylation upon IFN-γ stimulation (Figure S5K). Conversely, mutation of Tyr701 to a non-phosphorylatable form [STAT1(Y701F)], had no impact on Lys637 acetylation (Figure S5L). Together, these results identify Lys637 acetylation as a previously unknown regulatory modification that compromises STAT1 function by preventing proper homodimerization, thereby reducing its transcriptional activity and ultimately blunting tumor cell response to IFN-γ in cetuximab-resistant HNSCC.

### Inflammatory cytokines IFN-β and TNF-α trigger STAT1 inactivation in cetuximab-resistant HNSCC

**Figure 6:**
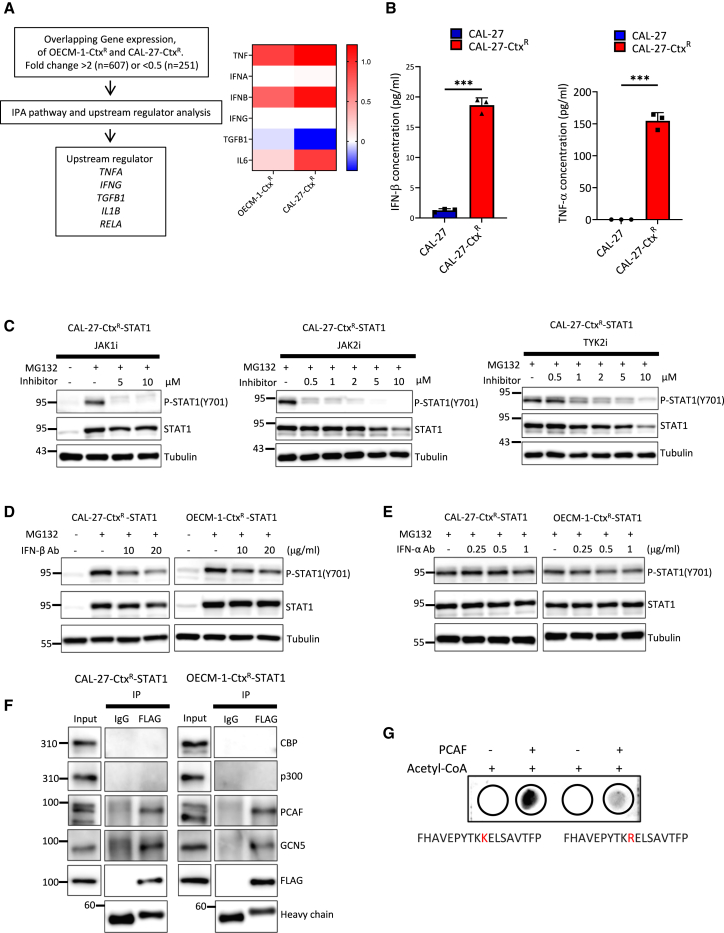
IFN-β and TNF-α as potential upstream regulators of STAT1 inactivation in cetuximab-resistant HNSCC (A) Schematic representation of the identification of upstream regulators using Ingenuity Pathway Analysis in OECM-1-Ctx^R^ and CAL-27-Ctx^R^ cells (left). Expression levels of the indicated genes based on RNA sequencing in OECM-1-Ctx^R^ and CAL-27-Ctx^R^ cells compared to parental cells (right). (B) ELISA of IFN-β (left) and TNF-α (right) concentrations in conditioned media from CAL-27 and CAL-27-Ctx^R^ cells (*n* = 3, with two technical replicates each). Data are presented as mean ± SD. Statistical analyses were performed using unpaired Student’s *t* test. ∗∗∗*p* < 0.001. (C) Representative western blot of the indicated proteins in CAL-27-Ctx^R^ cells transfected with STAT1 (CAL-27-Ctx^R^-STAT1) and treated with MG132 (10 μM) combined with JAK1 (left), JAK2 (middle), and TYK2 inhibitors (right) at the indicated concentrations for 16 h. GAPDH was a loading control. The experiments were performed in triplicate. (D) Representative western blot of the indicated proteins in CAL-27-Ctx^R^ (left) and OECM-1-Ctx^R^ (right) cells transfected with STAT1 (CAL-27-Ctx^R^-STAT1 and OECM-1-Ctx^R^-STAT1) and treated with MG132 (10 μM) and IFN-β-neutralizing antibody at indicated concentrations for 16 h. GAPDH was the loading control. The experiments were performed in triplicate. (E) Representative western blot of STAT1 Tyr701 phosphorylation in OECM-1-Ctx^R^ (left) and CAL-27-Ctx^R^ (right) cells transfected with STAT1 (CAL-27-Ctx^R^-STAT1 and OECM-1-Ctx^R^-STAT1) and treated with MG132 (10 μM) combined with an IFN-α-neutralizing antibody at indicated concentrations for 16 h. α-tubulin was the loading control. Experiments were duplicated. (F) Representative co-immunoprecipitation and western blot analyses to investigate the interaction between STAT1 and histone acetyltransferases in the CAL-27-Ctx^R^ and OECM-1-Ctx^R^ cells transfected with STAT1 (CAL-27-Ctx^R^-STAT1 and OECM-1-Ctx^R^-STAT1). The cells were then treated with MG132 (10 μM) for 16 h. The experiments were performed in triplicate. (G) Representative *in vitro* acetylation assay. Biotin-labeled synthetic peptides, corresponding to the sequence encompassing STAT1 lysine 637 (K637) or a mutant variant where K637 was substituted with arginine (K637R), were utilized. These peptides were incubated in the presence or absence of the histone acetyltransferase (PCAF) and with acetyl-coenzyme A (acetyl-CoA). Following the incubation, the reaction products were analyzed by dot blot for assessing acetylation levels. The experiments were performed in triplicate. See also Figure S6.

To identify upstream regulators of STAT1 modifications in cetuximab-resistant cells, we performed Ingenuity Pathway Analysis of differentially expressed genes. This analysis revealed *TNFA, IFNG*, *TGFB1*, *IL1B*, and *RELA* as key upstream regulators in resistant cells (Figure 6A, left panel). When integrating these findings with the expression profiles of inflammatory cytokines in resistant cells (Figure 6A, right panel) and our earlier proteogenomic analysis of resistance evolution (Figure 2), IFN-β and tumor necrosis factor alpha (TNF-α) emerged as the most promising targets due to their consistent upregulation across both resistant sublines. Supporting this notion, we detected significantly elevated levels of both IFN-β and TNF-α in conditioned media from resistant cells compared to parental cells (Figure 6B). A trend toward higher serum IFN-β levels was noted in HNSCC patients who showed poor response to ICB therapy (Figure S6A).

We next investigated the regulation of STAT1 modifications by these cytokines. Since IFN-JAK/TYK signaling mediates STAT1 Tyr701 phosphorylation,^37^ we examined the roles of specific JAK family members. In cetuximab-resistant cells expressing reconstituted STAT1, inhibition of JAK1, JAK2, or TYK2, abolished IFN-γ-mediated STAT1 Tyr701 phosphorylation (Figures 6C and S6B). Inhibition of the type I IFN receptor IFNAR1 prevented STAT1 Tyr701 phosphorylation in resistant cells (Figure S6C). Notably, while IFN-β-neutralizing antibodies reduced STAT1 Tyr701 phosphorylation (Figure 6D), IFN-α neutralization had no effect (Figure 6E). The neutralizing efficacy of anti-IFN-α antibody was confirmed using wild-type cells treated with IFN-α (Figure S6D). These results identify IFN-β as the key upstream regulator of STAT1 phosphorylation through JAK1/JAK2/TYK2.

In parallel, we investigated which acetyltransferase mediates STAT1 Lys637 acetylation in resistant cells. Co-immunoprecipitation experiments identified two candidates, GCN5 and PCAF, that physically interact with STAT1 (Figure 6F). Pharmacological inhibition of PCAF with NSC694621, but not GCN5 inhibition with MB-3, restored IFN-γ response (Figures S6E and S6F). Knockdown of PCAF restored the expression of PD-L1 and IRF1 in cetuximab-resistant cells (Figure S6G). The interaction between PCAF and endogenous STAT1 was confirmed (Figure S6H). *In vitro* acetylation assays using STAT1-derived peptides demonstrated that PCAF directly acetylates STAT1 at Lys637 (Figure 6G). We examine the expression of STAT1 Lys637 acetylation in a panel of wild-type HNSCC cell lines. The results revealed heterogeneous expression, with overall relatively low levels of STAT1 Lys637 acetylation across these HNSCC cell lines (Figure S6I). However, TNF-α treatment induces a greater increase in STAT1 Lys637 acetylation in cetuximab-resistant CAL-27 cells than in parental CAL-27 counterparts (Figure S6J), indicating that TNF-α-mediated acetylation is context dependent and more prominent in the resistant setting. Consistently, neutralization of TNF-α reduces STAT1 Lys637 acetylation (Figure S6K). Together, these findings reveal that inflammatory signaling orchestrates dual post-translational modifications of STAT1: IFN-β induces JAK1/JAK2/TYK2-dependent Tyr701 phosphorylation, leading to STAT1 degradation, while TNF-α promotes PCAF-mediated Lys637 acetylation, resulting in functional inactivation. This coordinated regulation ultimately impairs tumor cell responsiveness to IFN-γ in cetuximab-resistant HNSCC.

### Lys637 acetylation of STAT1 is associated with worse response to ICB treatment in HNSCC

**Figure 7:**
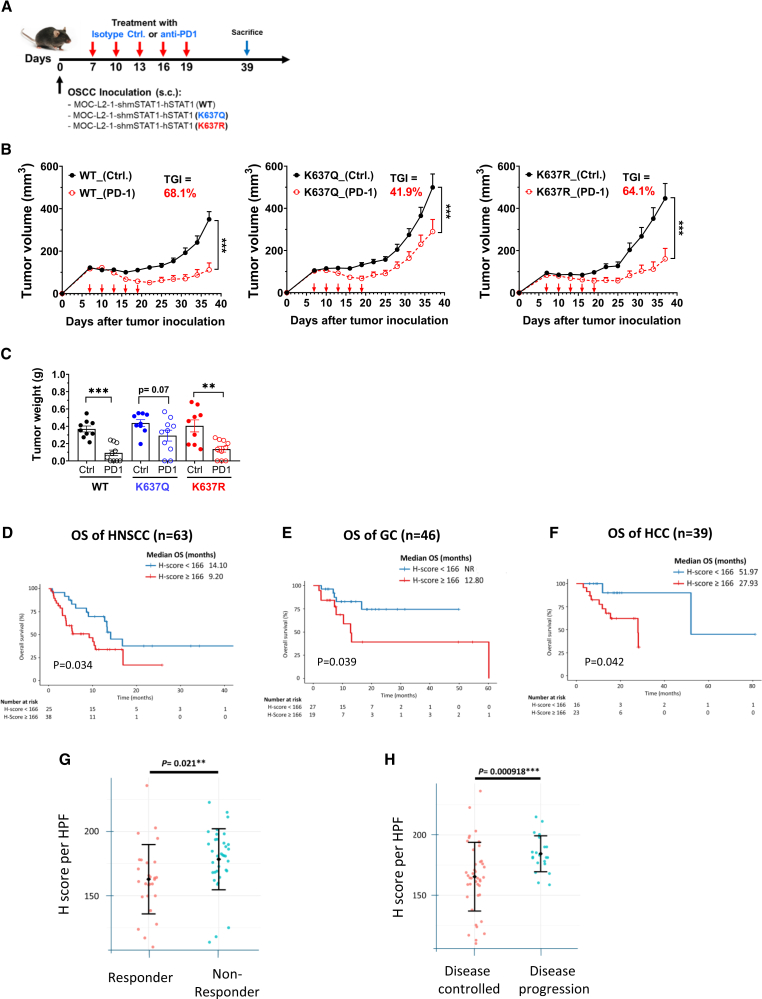
STAT1 Lys637 acetylation correlates with poor response to ICB therapy (A) Schematic of a syngeneic murine oral cancer model receiving anti-PD1 injection. The murine oral squamous cell carcinoma cell line MOC-L2-1 with Stat1 knockdown (shmStat1) and reconstituted with human STAT1 (hSTAT1(WT) or hSTAT1(K637Q) or hSTAT1(K637R)) was inoculated subcutaneously into C57BL/6J mice until tumors reached a volume of 100 mm^3^. Five doses of anti-PD1 or isotype IgG were administered to tumor-bearing mice. *n* = 9–10 per group. (B) Tumor growth inhibition (TGI, %) calculated as the relative change in tumor volume between day 0 and day 38 in different groups. Data presented as mean ± SEM. ∗∗∗*p* < 0.001. (C) Tumor weight in the mouse experiments. Data presented as mean ± SEM. ∗∗*p* < 0.01; ∗∗∗*p* < 0.001; ns, not significant. (D) Kaplan-Meier overall survival curves for HNSCC patients (*n* = 63) stratified by H-score cutoff of 166 with median follow-up of 8.0 months (range 0.5–45.1). (E) Kaplan-Meier overall survival curves for GC patients (*n* = 46) stratified by H-score cutoff of 166 with median follow-up of 9.7 months (range 1.97–60.2). (F) Kaplan-Meier overall survival curves for hepatocellular carcinoma (HCC) patients (*n* = 39) stratified by H-score cutoff of 166 with median follow-up of 15.5 months (range 3.1–81.1). (G) Comparison of STAT1 Lys637 acetylation levels between HNSCC responders (*n* = 27) and non-responders (*n* = 36) to ICB treatment. Statistical analyses were performed using an unpaired Student’s *t* test. ∗∗*p* < 0.01. (H) Comparison of STAT1 K637 acetylation levels between HNSCC disease control patients (*n* = 42) and those with progressive disease (*n* = 21) following ICB therapy. Statistical analyses were performed using an unpaired Student’s *t* test. ∗∗∗*p* < 0.001. See also Figure S7, Tables S9, S10, S11, and S12.

We validated the significance of STAT1 Lys637 acetylation in ICB response using a murine syngeneic OSCC model. The murine OSCC cell line MOC-L2-1 was knocked down for *mStat1* and reconstituted with either a wild-type hSTAT1, Lys637 unacetylatable mutant hSTAT1(K637R), or a Lys637 acetylation-mimicking mutant hSTAT1(K637Q). A reduced response to mIFN-γ was confirmed in cells expressing hSTAT1(K637Q) compared with those expressing wild-type hSTAT1 or hSTAT1(K637R) (Figure S7A). We inoculated these cell lines into C57BL/6J mice and administered five doses of anti-PD1 or isotype IgG once the tumors reached 100 mm^3^. Mice bearing MOC-L2-1-shmStat1 cells expressing wild-type hSTAT1 or hSTAT1(K637R) exhibited a more prominent antitumor effect from anti-PD1 compared to those expressing hSTAT1(K637Q) (Figures 7A–7C).

We next investigated the clinical relevance of STAT1 Lys637 acetylation in HNSCC samples. Using immunohistochemistry with validated scoring criteria (Figure S7B shows representative images), we examined paired patient samples collected before and after prolonged cetuximab treatment. Post-cetuximab samples exhibited a trend toward higher H-scores compared to pre-treatment specimens (Figure S7C). Patients who did not respond to cetuximab showed higher levels of STAT1 Lys637 acetylation compared to those who responded (Figure S7D).

To evaluate whether STAT1 Lys637 acetylation could serve as a predictive biomarker for ICB response independent of prior cetuximab exposure, we analyzed an independent cohort of cancer patients with tumor samples collected before ICB treatment. This cohort included 148 cases, comprising HNSCC (63 cases), gastric cancer (GC, 46 cases), and hepatocellular carcinoma (HCC, 39 cases). All patients received ICB therapies at TVGH between 2018 and 2025. Patient characteristics are summarized in Table S9. A prognostic cutoff was determined by time-dependent receiver operating characteristic (ROC) analysis using 12-month OS as the endpoint.^38^ This analysis identified an H-score of 166 as the optimal threshold, showing moderate discriminative ability (area under the curve, 0.685; sensitivity, 0.753; specificity, 0.572; Figure S7E; Table S10). Subgroup analyses yielded similar thresholds for each cancer type (GC 170, HCC 165, and HNSCC 167), with comparable performance metrics (area under the curve ranging from 0.625 to 0.708), supporting the use of a unified cutoff for cross-cancer comparisons.

Survival analysis revealed cancer-specific prognostic patterns. High H-scores were associated with reduced OS across all three types of cancer (Figures 7D–7F). In multivariate analysis, STAT1 Lys637 acetylation emerged as an independent prognostic factor for PFS in HNSCC (Table S11). Treatment response patterns varied by cancer type (Table S12). In HNSCC, high H-scores were significantly associated with lower objective response rates (Figure 7G) and lower disease control rates (Figure 7H). In contrast, H-score did not correlate with treatment response in GC or HCC (Figures S7F and S7G). Taken together, these findings suggest that STAT1 Lys637 acetylation has prognostic relevance in the context of immunotherapy, with the strongest and most consistent associations observed in HNSCC.

## Discussion

A central finding of our work is how therapeutic stress progressively reshapes tumor cell response to immune attack. While IFN-γ signaling is essential for immunotherapy efficacy,^25^^,^^26^ chronic therapeutic pressure induces cellular adaptations that systematically alter this pathway. Prolonged treatment of cetuximab leads to two critical post-translational modifications of STAT1, leading to dysfunction and degradation. This mechanistic insight provides molecular evidence for how prior therapy can compromise subsequent treatment response.

The evolution of treatment resistance through adaptation to inflammatory signals represents another critical aspect of our findings. The initial inflammatory response to cetuximab, characterized by enhanced IFN-γ and IFN-α signaling, gradually transitions to an immunosuppressive state through upstream regulators IFN-β and TNF-α. For TNF-α, our previous work and other studies have demonstrated that EMT confers cetuximab resistance^21^^,^^22^^,^^39^; the EMT transcriptional factor Snail induces TNF-α that promotes tumor-related inflammation and boosts EMT.^40^ Regarding IFN-β, sustained signaling has been implicated in acquired immunotherapy resistance by inducing PD-L1 and NOS2 expression in both tumor and dendritic cells, leading to the accumulation of regulatory T cells (Tregs) and myeloid cells and resulting in acquired resistance to ICB.^41^ Despite these findings, the anti-tumor effects of type I IFNs have been demonstrated in various tumor types.^42^ The detrimental effects of proinflammatory cytokines on ICB treatment and the potential of anti-cytokine agents to augment ICB efficacy remain unclear and warrant further investigation.

Our findings have immediate implications for clinical practice. The identification of STAT1 Lys637 acetylation as a potential biomarker for immunotherapy response provides a practical tool for treatment stratification. Moreover, understanding the dynamic nature of therapeutic adaptation suggests that the timing and sequence of treatments may be as crucial as their selection. The observation that longer intervals between cetuximab and ICB associated with better outcomes opens possibilities for optimizing treatment scheduling. These results also raise important questions about therapeutic resistance in broader contexts. First, does the STAT1-mediated adaptation mechanism we identified operate in other cancer types or with different targeted therapies? Second, could targeting the inflammatory phase prevent or delay resistance development? Future studies addressing these questions could help develop strategies to prevent or reverse therapy-induced adaptations. In conclusion, our study shows that therapeutic stress activates molecular switches that reshape tumor responses to subsequent ICB, offering a framework to understand treatment sequence effects and practical guidance for improving cancer therapy.

### Limitations of the study

A principal limitation of this study is the inability to directly model cetuximab resistance in immunocompetent murine systems. Cetuximab is a humanized antibody that specifically targets human EGFR and does not cross-react with murine EGFR. Although murine-specific anti-EGFR antibodies, such as 7A7, may partially mimic cetuximab activity,^43^ differences in binding epitopes and pharmacodynamics substantially limit the validity of direct comparisons. Consequently, generation of a murine cetuximab-resistant model in syngeneic systems is not feasible. Another limitation is that the cetuximab resistance signature (CRS) presented in Figure 2C demonstrates limited predictive capacity for anti-PD1 efficacy, likely due to its derivation from only two cell lines and the absence of integrated proteomic or other multiomic data. Future expansion of the platform to incorporate larger multiomic datasets, together with the implementation of AI-based predictive modeling, is anticipated to enhance predictive accuracy and facilitate clinical translation.

## Resource availability

### Lead contact

Further information and requests for resources, reagents, and samples should be directed to the lead contact, Muh-Hwa Yang (mhyang2@nycu.edu.tw).

### Materials availability

The materials and reagents used in this study are listed in the key resources table. Reagents generated in our laboratory in this study or previous studies are available upon request.

### Data and code availability

• RNA-seq and exome sequencing data have been deposited at GEO and are publicly available under accession numbers GEO: GSE261770, GSE261974, and GSE264007.
• Mass spectrometry data are available in the ProteomeXchange Consortium under accession numbers PXD054332, PXD054186, and PXD054113.
• This paper does not report the original code.
• Any additional information required to reanalyze the data reported in this work paper is available from lead contact upon request.

## Acknowledgments

All samples for this study were obtained from the Biobank of Taipei Veterans General Hospital, and the authors acknowledge the support of the Biobank. The authors acknowledge the technical services provided by the Genomics Center for Clinical and Biotechnological Applications of the Cancer and Immunology Research Center (National Yang Ming Chiao Tung University), supported by the National Core Facility for Biopharmaceuticals (NCFB) of the 10.13039/100020595National Science and Technology Council (NSTC 112-2740-B-A49-001 and 111-2321-B-A49-007, MOST 110-2740-B-A49A-501, MOST 109-2740-B-010-002, and MOST 108-2319-B-010-001). This work received financial support from grants provided by the National Council of Science and Technology (NSTC 114-2314-B-A49-068-MY3, 114-2320-B-A49-038, 113-2320-B-A49-001, 112-2320-B-A49-006, 111-2314-B-A49-030-MY3, and 110-2320-B-A49A-542 to M.-H.Y.), T-Star Center (NSTC 113-2634-F-039-001 to M.-C. Hung and M.-H.Y.), and NYCU Cancer Progression Research Center and Cancer & Immunology Research Center (to M.-H.Y.) from The Featured Areas Research Center Program within the framework of the Higher Education Sprout Project by the Ministry of Education and the 10.13039/501100004737National Health Research Institutes (NHRI-EX112-11215BI to M.-H.Y.), 10.13039/501100011912Taipei Veterans General Hospital (V112C-130 and V112E-002-2 to M.-H.Y.), and 10.13039/501100015835Department of Health, Taipei City Government (grant no. 11201-62-042).

## Author contributions

P.-H.C, conceptualization, methodology, data curation, and writing – original draft; K.-C.L., H.-L.W., Y.-W.C., and N.-Y.S., methodology; T.-H.C. and W.-C.W., software, data curation, formal analysis, and writing – original draft; Y.-S.T., J.-H.S., and Ju-Pei Chen, software, data curation, and formal analysis; G.-Y.C., W.-L.F., H.-M.W., H.-C.H., M.C. Hsieh, C.-H.H., M.-Y.L., Y.-F.C., H.-C.W., C.-Y.C., T.-L.H., C.-C.W., Y.-C.L., Jo-Pai Chen, W.-C.L., C.-Y.Y., C.-L.L., and P.-J.L., resources; P.-Y.C., resources and funding acquisition; S.-C.W., methodology and resources; M.-C. Hung, resources, funding acquisition, and writing – review & editing; M.-H.Y., conceptualization, investigation, resources, writing – review & editing, supervision, project administration, and funding acquisition.

## Declaration of interests

The authors declare no competing interests.

## STAR★Methods

### Key resources table

**Table:** 

REAGENT or RESOURCE	SOURCE	IDENTIFIER
**Antibodies**		

Rabbit recombinant monoclonal GAPDH (14C10)	Cell Signaling Technology	Cat# 2118 (also 2118L); RRID: AB_561053
Rabbit monoclonal Stat1 (D1K9Y)	Cell Signaling Technology	Cat# 14994; RRID: AB_2737027
Rabbit monoclonal Phospho-Stat1 (Tyr701) (58D6)	Cell Signaling Technology	Cat#9167; RRID: AB_561284
Rabbit monoclonal Stat2 (D9J7L)	Cell Signaling Technology	Cat# 72604; RRID:AB_2799824
Mouse monoclonal Stat3 (124H6)	Cell Signaling Technology	Cat# 9139; RRID:AB_331757
Rabbit monoclonal Stat4 (C46B10)	Cell Signaling Technology	Cat# 2653 RRID:AB_2255156
Rabbit polyclonal Stat5 Antibody	Cell Signaling Technology	Cat# 9363 RRID:AB_2196923
Rabbit monoclonal Stat6 (D3H4)	Cell Signaling Technology	Cat# 5397 RRID:AB_11220421
Rabbit monoclonal Phospho-Stat1 (Ser727) (D3B7)	Cell Signaling Technology	Cat# 8826; RRID: AB_2773718
Rabbit monoclonal IRF-1 (D5E4) XP®	Cell Signaling Technology	Cat# 8478; RRID: AB_10949108
Rabbit recombinant monoclonal PD-L1 (E1L3N®) XP®	Cell Signaling Technology	Cat# 13684; RRID: AB_2687655
Rabbit monoclonal EGF Receptor (D1P9C)	Cell Signaling Technology	Cat# 4267 (also 4267S, 4267L, 4267T, 4267P); RRID:AB_2246311
Rabbit polyclonal Phospho-EGF Receptor (Tyr1068)	Cell Signaling Technology	Cat# 2234 (also 2234S, 2234L); RRID:AB_331701
Rabbit monoclonal p44/42 MAPK (Erk1/2) (137F5)	Cell Signaling Technology	Cat# 4695 (also 4695P, 4695S); RRID:AB_390779
Rabbit recombinant monoclonal Phospho-p44/42 MAPK (Erk1/2) (Thr202/Tyr204) (D13.14.4E) XP®	Cell Signaling Technology	Cat# 4370 (also 4370L, 4370S, 4370P, 4370T); RRID:AB_2315112
Rabbit monoclonal Jak1 (6G4)	Cell Signaling Technology	Cat# 3344; RRID:AB_2265054
Rabbit polyclonal Phospho-Jak1 (Tyr1022/1023)	Cell Signaling Technology	Cat# 3331; RRID:AB_2265057
Rabbit monoclonal Jak2 (D2E12) XP	Cell Signaling Technology	Cat# 3230 (also 3230S, 3230P, 3230L); RRID:AB_2128522
Rabbit polyclonal Phospho-Jak2 (Tyr1007/1008)	Cell Signaling Technology	Cat# 3771; RRID: AB_330403
Rabbit monoclonal Tyk2 (D4I5T)	Cell Signaling Technology	Cat# 14193; RRID: AB_2798419
Rabbit polyclonal Phospho-Tyk2 (Tyr1054/1055)	Cell Signaling Technology	at# 9321; RRID: AB_2303972
Rabbit IFNGR1 (E444)	Cell Signaling Technology	Cat# 10405; RRID: AB_2797720
Rabbit polyclonal Ubiquitin	Cell Signaling Technology	Cat# 3933 (also 3933S); RRID: AB_2180538
HA-Tag (C29F4)	Cell Signaling Technology	Cat# 3724 (also 3724S); RRID: AB_1549585
Mouse Monoclonal Snail	Cell Signaling Technology	Cat# 3895; RRID:AB_2191759
Mouse monoclonal HLA Class 1 ABC antibody [EMR8-5]	Abcam	Cat# ab70328; RRID:AB_1269092
Mouse monoclonal Anti-Lysine, acetyl Antibody, Unconjugated, Clone 1C6	Abcam	Cat# ab22550; RRID:AB_447149
Anti-β-Actin Antibody	Sigma-Aldrich	Cat# A5441; RRID:AB_476744
Mouse monoclonal ANTI-FLAG® M2 antibody	Sigma-Aldrich	Cat# F1804; RRID:AB_262044
Mouse monoclonal Anti-alpha-Tubulin Antibody, Unconjugated, Clone B-5-1-2	Sigma-Aldrich	Cat# T6074; RRID:AB_477582
Mouse monoclonal SQSTM1/p62 (D-3)	Santa Cruz Biotechnology	Cat# sc-28359; RRID:AB_628279
Rat monoclonal InVivoMAb anti-mouse PD-1 (CD279)	Bio X Cell	Cat# BE0146; RRID:AB_10949053
InVivoPlus rat IgG2a isotype control	Bio X Cell	Cat# BE0089; RRID:AB_1107769
Human TNF-α Antibody	R&D systems	Cat# AF-210-NA;
Human IFN-β Antibody	R&D systems	Cat# AF814; RRID:AB_2122897
Rabbit polyclonal IFITM1 Antibody	Proteintech	Cat# 11727-3-AP; RRID:AB_2122083
Rabbit polyclonal IFITM2 Antibody	Proteintech	Cat# 12769-1-AP; RRID:AB_2122089
Rabbit polyclonal IFITM3 Antibody	Proteintech	Cat# 11714-1-AP; RRID:AB_2295684

**Bacterial and virus strains**		

ECOSOEMCompetentCells [Stabl3]	YeasternBiotech	FYE307-80VL

**Biological samples**		

20 slide specimens from 10 HNSCC patients pre and post cetuximab treatment for Multiplex Immunofluorescence (Figures 1G and 1H)	Taipei Veterans General Hospital	TVGH-IRB certificate 2019-04-001BC
8 specimens from 5 HNSCC patients pre or post cetuximab treatment for RNA sequencing (Figure S2K)	Taipei Veterans General Hospital	TVGH-IRB certificate 2019-04-001BC
42 serum specimens from 35 HNSCC patients treated with either pembrolizumab or nivolumab (Figure S6A)	Taipei Veterans General Hospital	TVGH-IRB certificate 2020-08-013BC
6 slide specimens from 3 HNSCC patients pre and post cetuximab treatment for STAT1 Lys637 acetylation antibody validation (Figures S7C and S7D)	Taipei Veterans General Hospital	TVGH-IRB certificate 2020-08-013BC
slide specimens from 63 HNSCC patients treated with either pembrolizumab or nivolumab (Figure 7D)	Taipei Veterans General Hospital	TVGH-IRB certificate 2020-08-013BC
Slide specimens from 46 gastric cancer and 39 hepatocellular carcinoma patients treated with immune checkpoint inihibitors (Figures 7E, 7F, S7F, and S7G)	Taipei Veterans General Hospital	TVGH-IRB certificate 2025-03-001CC

**Chemicals, peptides, and recombinant proteins**		

Recombinant Human IFN-γ	PEPROTECH	Cat#300-02
Recombinant Human TNF-α	PEPROTECH	Cat#300-01A
Cetuximab	Merck	L01XC06
Bafilomycin A_1_	Cayman Chemical	NSC 381866, CAS: 88899-55-2
MG132	Cayman Chemical	CAS:1211877-36-9
Solcitinib	MedChemExpress	CAS: 1206163-45-2
AZ 960	Cayman Chemical	CAS: 905586-69-8
PF-06826647	AOBIOUS INC	CAS: 2127109-84-4
HyClone RPMI-1640 Medium (1×)	cytiva	Cat# SH30027.01
HyClone Characterized Fetal Bovine Serum, CA Origin	cytiva	Cat#SH30396.03
Dulbecco’s Modified Eagle Medium (DMEM)	Gibco™	Cat# 12-100-046
Ampicillin	BioShop	CAS: 69-52-3
Tween 20	BioShop	Cat# TWN508; CAS: 9005-64-5
LB BROTH (MILLER)	BioShop	Cat# LBL407
AGAR, Bacteriological Grade	BioShop	Cat# AGR001; CAS: 9002-18-0
Tritos X-100	BioShop	Cat# TRX506; CAS: 9002-93-1
SODIUM CHLORIDE, Reagent Grade, min 99%	BioShop	Cat# TSOD002; CAS: 7647-14-5
MERCAPTOETHANOL, Biotechnology Grade	BioShop	Cat#MER002; CAS: 60-24-2
Potassium chloride	BioShop	Cat# POC308; CAS: 7447-40-7
Liquid acrylamide 29:1	CYRUSBIOSCIENCE	Cat#A3217
Phosphatase Inhibitor Cocktail 2	Sigma-Aldrich	Cat#P5726
Cycloheximide	Sigma-Aldrich	Cat# C7698; CAS:66-81-9
Thiazolyl Blue Tetrazolium Bromide	Sigma-Aldrich	Cat#M5655; CAS: 298-93-1
Tetramethylethylenediamine(TEMED)	Sigma-Aldrich	Cat#T9281; CAS: 110-18-9
Sodium aside	Sigma-Aldrich	Cat# S2002; CAS: 26628-22-8
Doxycycline	Sigma-Aldrich	Cat# D9891; CAS:4390-14-5
Ethylenediaminetetraacetic acid (EDTA)	Sigma-Aldrich	CAS:60-00-4
2-Propanol	Sigma-Aldrich	Cat# I9516; CAS: 67-63-0
1-Bromo-3-chloropropane	Sigma-Aldrich	Cat# B9673; CAS: 109-70-6
DMSO	Sigma-Aldrich	Cat# D2650; CAS: 67-68-5
Bromophenol Blue sodium salt	Sigma-Aldrich	Cat# B5525; CAS: 34725-61-6
Sodium phosphate dibasic	Sigma-Aldrich	Cat# S0876; CAS: 7558-79-4
Sodium phosphate monobasic	Sigma-Aldrich	Cat# S0751; CAS: 7558-80-7
Magnesium chloride	Sigma-Aldrich	Cat# M8266; CAS: 7786-30-3
Kaiser’s glycerol gelatine for microscopy	Sigma-Aldrich	Cat# 109242; CAS: 9000-70-8
Trident femto Western HRP Substrate	GeneTex	Cat#GTX14698;
Sodium Dodecyl Sulfate (SDS)	JT Baker®	4095-02;
TRIS (Base)	Bioman	Cat#TRS001; CAS:77-86-1
GLYCINE	Bioman	Cat#GLN011; CAS: 56-40-6
Agarose	Bioman	Cat#PB1200
GLYCEROL, Biotechnology Grade, min 99.7%	Bioman	Cat#GLY001; CAS: 56-81-5
TBE 5X Buffer	Bioman	Cat#TBE055000
PBS 10X buffer Sterile Solution	Bioman	Cat#PBS105000
Trizol	Invitrogen™	15596018; CAS: 9048-46-8
Dynabeads™ Protein G	Invitrogen™	10004D
Dynabeads™ Protein A	Invitrogen™	10002D
Fast SYBR™ Green Master Mix	Thermo Scientific	4385612
Pierce™ Bovine Serum Albumin Standard Ampules	Thermo Scientific	23209
HCL	CHONEYE PURE CHEMICALS	N/A
95% Alcohol	ECHO CHEMICAL CO. LTD	N/A
METHYL ALCOHOL	Macron Fine Chemicals™	CAS: 67-56-1
cOmplete(TM), Mini, EDTA-free Protease Inhibitor Cocktail Tablets provided in EASYpacks	Roche	04693159001
T-Pro NTR III (non-liposomal transfection reagent II)	T-Pro Biotechnology	NO.JT97-N006M

**Critical commercial assays**		

Human Interferon beta ELISA Kit	Abcam	ab278127
Nuclear/Cytosol Fractionation Kit	Abcam	ab289882
Stat1 EMSA Kit	Signosis N-acetyl-L-cysteine (NAC) (#A7250)	GS-0043
Luciferase Assay System	Promega	Cat#E1501
Human TNF-alpha Quantikine ELISA Kit	R&D Systems	Cat#DTA00D
Human IFN-beta Quantikine ELISA Kit	R&D Systems	Cat# DIFNB0
NucleoBond Xtra Midi kit	MACHEREY-NAGEL	Item number: 740410.50
GenepHlow™ Gel/PCR Kit	Geneaid	Cat# DFH300
Presto™ Mini Plasmid Kit	Geneaid	Cat# PDH300
QIAamp DNA Micro Kit	QIAGEN	Cat# QIA56304
Opal Polaris 7 Color IHC Detection Kits	Akoya Biosciences	Cat# NEL861001KT
Novolink Polymer Detection Systems	Leica Biosystems	Cat# RE7280-K
HiScript I Reverse Transcriptase	BIONOVAS	Cat# AM0670-1000

**Deposited data**		

Affinity-based mass spectrometry Mass spectrometry of OECM-1 and CAL-27 cetuximab-resistant sublines reconstituted with STAT1	This paper	PXD054332 PXD054186 PXD054113
Raw and analyzed data Whole exome sequencing of HNSCC (CAL27 and OECM-1) wild type and resistant cell lines	This paper	SRA: PRJNA1089712 (https://www.ncbi.nlm.nih.gov/sra)
Raw and analyzed data Genes expression of OECM-1 and CAL-27 wild-type (WT)/cetuximab-resistant sublines	This paper	GEO: GSE261770
Raw and analyzed data Genes expression between patients pre and post-cetuximab treatment	This paper	GEO: GSE261974
Raw and analyzed data Gene expression of OECM-1 treated with cetuximab in different passages.	This paper	GEO: GSE264007

**Experimental models: Cell lines**		

293 [HEK-293]	ATCC	Cat# CRL-1573 ™
OECM-1	Dr. Kuo-Wei Chang (National Yang Ming Chiao Tung University of Taiwan)	Yang and Meng^44^
CAL-27	were originally from ATCC	Cat# CRL-2095 ™
2fTGH-U3A	ECACC	Cat# 12021503
OECM-1-Cetuximab-resistant cell	This paper	N/A
CAL27-Cetuximab-resistant cell	This paper	N/A
MOC-L2	Dr. Kuo-Wei Chang (National Yang Ming Chiao Tung University of Taiwan)	Chen et al.^31^
MOC-L2-1	This paper	N/A
FaDu	were originally from ATCC	Cat# HTB-43™
SAS	were originally from AcceGen	Cat# ABL-TC0611
HSC-3	were originally from AcceGen	Cat# ABL-TC0290

**Experimental models: Organisms/strains**		

Mouse:C57BL/6JNarl	National Laboratory Animal Center (NLAC, Taiwan)	https://www.nlac.narl.org.tw/eng/index.asp

**Oligonucleotides**		

Short hairpin RNA against mouse STAT1 CCGGGGACTAGAGTGCGAGTATTTGCT CGAGCAAATACTCGCACTCTAGTCCTT TTTG	National RNAi Core Facility, Academia Sinica, Taiwan https://rnai.genmed.sinica.edu.tw/?wicket:interface=:2:rnaiHome::ILinkListener	TRCN0000235837
Primers for quantitative PCR, see Table S14	This paper	N/A
Short hairpin RNA against mouse STAT1 CCGGCCGAAGAACTTCACTCTCTTAC TCGAGTAAGAGAGTGAAGTTCTTCGG TTTTTG	National RNAi Core Facility, Academia Sinica, Taiwan https://rnai.genmed.sinica.edu.tw/?wicket:interface=:2:rnaiHome::ILinkListener	TRCN0000235837

**Recombinant DNA**		

pLV-WT-STAT1	Cheon et al.^45^	Cat#71454 RRID:Addgene_71454 http://n2t.net/addgene:71454
pLV-Y701F-STAT1	This paper	N/A
pLV-S727A-STAT1	This paper	N/A
pLV-K637R-STAT1	This paper	N/A
pLV-K637Q-STAT1	This paper	N/A

**Software and algorithms**		

ImageJv1.53e	Schneider et al., 2012^46^	https://imagej.net/ij/list.html
Ingenuity Pathway Analysis (IPA)	QIAGEN	https://digitalinsights.qiagen.com/
GraphPad Prism 8	GraphPad Software, San Diego, CA	https://www.graphpad.com/
DAVID Bioinformatics Resources 6.8	LHRI (Huang et al., 2009)^47^	https://david.ncifcrf.gov/
Gene Set Enrichment Analysis (GSEA)	UC San Diego and Broad Institute (Subramanian et al., 2005)^48^	https://www.gsea-msigdb.org/gsea/index.jsp

### Experimental model and study participant details

#### Patient enrollment and clinical data analysis of the Taiwan Head and Neck Society (THNS) registry and Taipei Veterans General Hospital cohorts

Clinical data analysis was approved by the Institutional Review Board (IRB) of Taipei Veterans General Hospital (TVGH) under case number 2020-08-013BC. Recurrent/metastatic HNSCC patients who received cetuximab treatment at 13 institutions and two branch hospitals in Taiwan were enrolled in the THNS registry. These institutions included TVGH, Chang Gung Memorial Hospital, New Taipei City Municipal TuCheng Hospital, National Taiwan University Hospital, E-Da Cancer Hospital, National Cheng Kung University Hospital, China Medical University Hospital, MacKay Memorial Hospital, Kaohsiung Medical University Hospital, Kaohsiung Chang Gung Memorial Hospital, Chung Shan Medical University Hospital, Changhua Christian Hospital, Taichung Veterans General Hospital, National Taiwan University Hospital Yunlin Branch, and the Chi Mei Medical Center. A total of 1,434 patients were enrolled between January 1, 2017, and June 6, 2022, and clinical data were retrospectively collected. For analysis, TVGH cases were separated into independent cohorts. Within the THNS cohort, we focused on two specific patient groups: (a) patients who received cetuximab followed by immunotherapy (cetuximab-resistant group, *n* = 104), and (b) patients who received immunotherapy followed by cetuximab (cetuximab-naïve group, *n* = 60). In the TVGH cohort, patients with recurrent/metastatic HNSCC treated between January 26, 2015, and October 29, 2020, were included. Two groups were analyzed: (a) patients treated with cetuximab followed by immunotherapy (cetuximab-resistant group, *n* = 35), and (b) patients treated with immunotherapy without prior cetuximab exposure (cetuximab-naïve group, *n* = 54). The cetuximab density index (CDI) was defined as follows: CDI = (accumulated cetuximab dose (mg))/[(interval of cetuximab treatment (months) × interval between the end of cetuximab and the beginning of immunotherapy treatment (months)].

#### Mice

C57BL/6 mice were obtained from the National Laboratory Animal Center (NLAC; Taiwan). All animal experiments adhered to the guidelines and protocols approved by the Institutional Animal Care and Utilization Committee of the National Yang Ming Chiao Tung University (IACUC certificate No. 110428). Female mice aged 6–8 weeks were used in all experiments.

#### Cell lines

The cells used in the experiments were authenticated using STR profiling and tested negative for mycoplasma contamination. HEK293T (RRID: CVCL_0063), CAL-27 (RRID:CVCL_1107), SAS (RRID:CVCL_1675), HSC-3 (RRID:CVCL_1288), 2fTGH-U3A (RRID:CVCL_9469), and MOCL2-1 (RRID:CVCL_S518) cells were cultured in Dulbecco’s modified Eagle’s medium (DMEM; Gibco, Carlsbad, CA, USA). OECM1 (RRID: CVCL_6782) and FaDu (RRID:CVCL_1218) were cultured in Roswell Park Memorial Institute medium (RPMI, Gibco, Carlsbad, CA, USA). The HNSCC cell line FaDu, CAL-27 and the human embryonic kidney cell line HEK293T were obtained from the American Type Culture Collection (ATCC, Rockville, MD, USA). The human head and neck cancer cell line OECM-1 and murine oral squamous cell carcinoma cell line MOCL2-1 were provided by Dr. Kuo-Wei Chang (National Yang-Ming Chiao Tung University, Taiwan). The human sarcoma cell line 2fTGH-U3A was purchased from the European Collection of Authenticated Cell Cultures (ECACC, Salisbury, SP4 0JG, UK). The HNSCC cell line SAS and HSC-3 were obtained from AcceGen (New Jersey, USA).

### Method details

#### Tumor samples for experimental analyses

To analyze the changes induced by cetuximab treatment in HNSCC, we examined samples from 10 patients treated at Taipei Veterans General Hospital (TVGH). Multiplex immunofluorescent staining was performed on paired pre-treatment and post-treatment tumor tissues (10 paired samples, comprising a total of 146 regions of interest [ROIs]; Figures 1G and 1H). In addition, RNA sequencing was conducted on 3 pre-treatment and 5 post-treatment samples (Figure S2K), and immunohistochemistry (IHC) was used to evaluate STAT1 acetylation at Lys637 (Figures S7C and S7D). To assess the potential of Lys637-acetylated STAT1 as a predictive biomarker for immune checkpoint blockade (ICB) response, we conducted a retrospective analysis of patients with advanced solid tumors treated with immunotherapy at TVGH between January 2018 and February 2025. This cohort included individuals with gastric cancer (GC), hepatocellular carcinoma (HCC), and HNSCC. Inclusion criteria were histologically confirmed advanced malignancy, treatment with anti-PD-1/PD-L1 agents, age ≥20 years, receipt of ≥2 cycles of immunotherapy, and availability of sufficient tumor tissue for IHC analysis. Patients with concurrent active secondary malignancies, insufficient tissue specimens, or incomplete treatment records were excluded. A total of 63 HNSCC, 46 GC, and 39 HCC cases met the eligibility criteria and were included in the analysis. Tumor tissues from these patients were subjected to IHC staining to assess STAT1 Lys637 acetylation. Patient characteristics are summarized in Table S9.

#### Statistical analysis and cutoff determination for human tumor samples

H-score distribution normality was assessed using Shapiro-Wilk testing and Quantile-Quantile plots. Given significant deviation from normality (W = 0.96268, *p* = 0.0005), non-parametric methods were employed for subsequent analyses. A unified H-score cutoff was determined using time-dependent ROC analysis with 12-month OS as the primary endpoint across the entire cohort. The optimal threshold was identified by maximizing the Youden index.^44^ This unified cutoff was applied consistently across all cancer types to enable valid cross-comparisons and avoid overfitting bias in smaller subgroups. All statistical analyses were performed using R software version 4.1.0 (R Foundation for Statistical Computing, Vienna, Austria). A two-sided *p*-value <0.05 was considered statistically significant.

#### Outcome assessment and survival analysis

Treatment response was evaluated using Response Evaluation Criteria in Solid Tumors (RECIST) version 1.1 criteria^45^ every 2–3 months. For HCC patients, modified RECIST (mRECIST) was additionally applied.^49^ OS was defined as time from immunotherapy initiation to death, and PFS as time to disease progression or death. Patients were stratified into high or low H-score groups based on the unified cutoff. Kaplan-Meier survival curves were constructed with log rank testing for group comparisons. Fisher’s exact test was used for categorical variables.

#### Generation of Lys637-acetylated STAT1-specific antibodies and validation of antibody specificity

Antibodies specific to Lys637-acetylated STAT1 were generated using synthetic acetylated peptides corresponding to the region surrounding lysine 637 of STAT1. BALB/c mice (6–8 weeks old) were immunized subcutaneously with KLH-conjugated acetylated peptide emulsified in complete Freund’s adjuvant (Sigma, Cat. No. F5881), followed by four booster immunizations at two-week intervals using KLH-conjugated acetylated peptide in incomplete Freund’s adjuvant (Sigma, Cat. No. F5506). For hybridoma generation, spleen cells from immunized mice were fused with P3/NS1/1-Ag4-1 myeloma cells (ATCC TIB-18) using polyethylene glycol (PEG 1500; Roche, Cat. No. 783641), following standard protocols. The resulting hybridomas were plated in 96-well plates, and supernatants were collected two weeks later for screening by ELISA. Positive clones were preserved for further validation. For ELISA screening, 96-well plates were coated overnight at 4°C with 20 μg/mL of KLH-conjugated acetylated peptide, acetylated peptide, or unmodified peptide in carbonate buffer (pH 9.5). Plates were washed three times with washing buffer (0.05% Tween 20 in PBS), blocked with 200 μL of blocking buffer (0.5% BSA [w/v], 0.05% Tween 20 in PBS) for 2 h at room temperature, and washed again three times. Then, 100 μL of hybridoma supernatant or diluted antibody was added and incubated for 2 h. After five washes, horseradish peroxidase (HRP)-conjugated goat anti-mouse IgG diluted in dilution buffer (0.1% BSA [w/v], 0.05% Tween 20 in PBS) was added and incubated for 1 h. Following five additional washes, TMB substrate (BD, Cat. No. 555214) was added and allowed to develop for 15 min in the dark. The reaction was stopped using 2N sulfuric acid, and absorbance was measured at 450 nm. ELISA results are presented in Table S13.

#### Cell culture

Cells were passaged every two days, and all cells were used within 20 passages after thawing. Cultured medium was supplemented with 10% (v/v) fetal bovine serum (FBS) (Gibco, Carlsbad, CA, USA) and 1% penicillin/streptomycin (Gibco, Carlsbad, CA, USA). To generate cetuximab-resistant cell lines, cells were cultured in the corresponding medium containing 500 μg/mL cetuximab (Merck KGaA, Darmstadt, Germany) for 30 passages. To induce the IFN-γ response, cells were treated with 100 ng/mL IFN-γ (PeproTech, 300-02-100UG) in serum-free medium for 24 h. For inhibitor treatment, cells were treated with different inhibitors: 100 nM of the autophagic inhibitor bafilomycin A1 (Sigma, #B1793), 20 μM of the proteasome inhibitor MG132 (Cayman chemicals, Cat# 13697). The cells were then treated with inhibitors for 18 h for analysis.

#### Plasmid construction and cell line transduction

To generate the pLV-STAT1(Y701F), pLV-STAT1(S727A), pLV-STAT1(Y701F/S727A), pLV-STAT1(K637R), and pLV-STAT1(K637Q) plasmids, human STAT1 cDNA was amplified from p-LV-STAT1 (RRID: Addgene_71454). Stable cell lines expressing these plasmids or a control vector were established using a lentiviral expression system. For mouse Stat1 knockdown, MOCL2-1 cells were transduced with a mouse Stat1 3′ UTR shRNA (TRCN0000235837). For human PCAF knockdown, OECM-1-Ctx^R^-STAT1 and CAL-27-Ctx^R^-STAT1 were transduced a PCAF shRNA (TRCN0000018530). For human Smurf1 and PDLIM2 knockdown, OECM-1-Ctx^R^ and CAL-27-Ctx^R^ were transduced with Smurf1 (TRCN0000003471) (TRCN0000003473) and PDLIM2 (TRCN0000154971) (TRCN0000155146). For lentivirus production, 10 μg of the expression vector, 9 μg of the pCMVΔR8.91 plasmid, and 2.5 μg of the pMD.G envelope plasmid was transfected into 293T cells using the T-Pro P-Fect Transfection Reagent (JT97-N005M). Experiments with cells expressing pLV-STAT1(Y701F), pLV-STAT1(S727A), pLV-STAT1(Y701F/S727A), pLV-STAT1(K637R), and pLV-STAT1(K637Q) were conducted using early passages (P2–P5). The transcriptional activities of pLV-STAT1(K637R) and pLV-STAT1(K637Q) were assessed using the pGreenFire1-STAT1 Lentivector (SBI, CA, USA), which contains STAT1 transcriptional response elements.

#### Immunoblotting and immunoprecipitation

Cells were lysed in a buffer containing 50 mM Tris-HCl (pH 7.4), 150 mM NaCl, 1 mM EDTA, 1% Triton X-100, and 5% glycerol supplemented with 1X protease inhibitor (Roche, Mannheim, Germany) and 1X phosphatase inhibitor (Sigma-Aldrich, Cat# P5726). Lysates were then transferred to microtubes and incubated on ice for 20 min. After incubation, the lysates were centrifuged at 14,000 rpm for 20 min at 4°C and the supernatants were collected. The protein concentration was measured using an Infinite M200 (Tecan, Switzerland) in conjunction with a BCA protein assay (Thermo Scientific Pierce BCA Protein Assay, Waltham, MA, USA). All samples were adjusted to equal protein concentrations by adding appropriate volumes of lysis buffer and 6X sample buffer. The mixture was then heated at 95°C for 10 min. Protein extracts were resolved by SDS-PAGE at appropriate concentrations and transferred to PVDF membranes (Millipore, Billerica, MA, USA). Transfer was performed at 300 mA on ice for 1.5 h. The membranes were blocked in PBST containing 5% skim milk at room temperature for at least 1 h and then incubated with specific primary antibodies overnight at 4°C. Following incubation, the membranes were washed with PBST and incubated with HRP-conjugated secondary antibodies (Jackson ImmunoResearch, 115-035-003 and 111-035-003) in 5% skim milk at room temperature for 1 h. The membranes were washed thrice with PBST before and after incubation. Detection was performed using a GE AI600 imaging system (GE Healthcare Inc., Marlborough, MA, USA) following incubation with an ECL substrate (Millipore, Billerica, MA, USA). For quantitative analysis of Western blots, all protein signals were first normalized to their corresponding tubulin signal to control for loading variations. To specifically assess the level of STAT1 acetylation, the signal corresponding to STAT1 acetylated at Lys637 was further normalized to the total STAT1 protein signal in each sample. For immunoprecipitation, the lysates were mixed with an equal volume of Co-IP buffer (150 mM NaCl, 1% NP-40, 1 mM EDTA, 5% glycerol, and 50 mM Tris-HCl, pH 7.5). The primary antibody or IgG control (Santa Cruz Biotechnology, Dallas, Texas, U.S.A.) was added to the lysates, and the mixture was incubated at 4°C on a rotary device for 1 h. Dynabeads Oligo(dT)25 (Thermo Scientific Pierce, Waltham, MA, USA) was added to block non-specific binding for 1 h. Beads were collected using a magnet and gently washed with TNTG buffer before immunoblotting. The beads were resuspended in 6X sample buffer and heated at 95°C for 10 min. Lysine-acetylated proteins were purified using anti-acetyl-lysine antibody-coated agarose (Cat. #ICP0388; ImmuneChem Pharmaceuticals Inc., Burnaby, Canada).

#### RT-qPCR

RNA from 293T, 2fTGH-U3A, OECM-1, CAL-27, and cetuximab-resistant sublines was extracted using 1 mL of Trizol (Invitrogen). The lysates were centrifuged at 12,000 × g for 15 min, and the colorless aqueous phase was transferred to new microtubes containing isopropanol (Sigma-Aldrich, Cat# I9516). The mixture was then centrifuged at 12,000 × g for 10 min and washed with 75% ethanol (Cat# E7023; Sigma-Aldrich). The RNA concentration was quantified using a NanoDrop spectrophotometer (Thermo Fisher Scientific, Waltham, MA, USA). The extracted RNA was reverse transcribed into cDNA using HiScript I Reverse Transcriptase (Bionovas Biotechnology, Toronto, Canada) according to the manufacturer’s instructions. The resulting cDNA was used as a template for subsequent PCR amplification using gene-specific primers. RT-qPCR was performed using Fast SYBR Green Master Mix (Thermo Scientific Pierce, Waltham, MA, USA) in 96-well plates, and the reactions were read using the StepOnePlus real-time PCR system (Applied Biosystems Inc., Foster City, CA, USA). The primers used for amplification are listed in Table S14.

#### RNA sequencing

RNA isolation was performed as previously described. RNA concentration was measured using a Qubit RNA High Sensitivity Assay Kit (Thermo Fisher, Cat# Q32855), and RNA integrity was assessed using an RNA 6000 Pico chip (Agilent, Cat# 5067-1513) on an Agilent 2100 Bioanalyzer. Libraries were prepared from the extracted RNA using the QIAseq FastSelect-rRNA HMR Kit (QIAGEN, Hilden, Germany) and the Stranded mRNA Prep Ligation Kit (Illumina, San Diego, CA, USA), following the manufacturer’s protocols. Sequencing was conducted on a NextSeq 550 (Illumina, San Diego, CA, USA) in high-output mode at the Cancer and Immunology Research Center core facility, National Yang Ming Chiao Tung University, Taiwan.

#### Immunofluorescent staining

Cells were seeded onto Millicell EZ Slides (Sigma-Aldrich, Cat# PEZGS0816) and treated with IFN-γ (100 ng/mL, PeproTech, Cat# 300-02-100UG) for 1 h. The cells were then fixed with 4% paraformaldehyde, permeabilized with 0.5% Triton X-100, and blocked with 1% BSA. Primary antibodies against STAT1 (Cell Signaling Technology, Danvers, MA, USA) were used, and Hoechst 33342 (Thermo Fisher Scientific, Waltham, MA, USA) was used for nuclear staining. Images were captured using a ZEISS LSM 900 confocal microscope (Carl Zeiss Microscopy, White Plains, NY, USA) with a 40× oil objective (Plan-Apochromat 40×/1.3 Oil DIC M27). The images were acquired sequentially using a confocal laser scanning microscope and analyzed using ZEN Microscopy 3.3 Software.

#### Mass spectrometry

Details of mass spectrometry protocol have been described previously.^40^ Protein solutions were reconstituted in 50 mM ammonium bicarbonate (ABC, Sigma) and disulfide bonds were reduced using 10 mM dithiothreitol (DTT, Merck) at 56°C for 45 min. Cysteine residues were alkylated with 55 mM iodoacetamide (IAM, Sigma) at 25°C for 30 min. Proteins were digested overnight at 37°C using sequencing-grade modified porcine trypsin (Promega) at a protein-to-trypsin ratio of 20:1. After digestion, the peptides were desalted, dried using a vacuum centrifuge, and stored at −80°C until further use. The digested peptides were reconstituted in HPLC buffer A (0.1% formic acid) and separated on a reverse-phase column (Zorbax 300SB-C18, 0.3 × 5 mm; Agilent Technologies). Chromatographic separation was performed on a column (Waters BEH 1.7 μm, 100 μm I.D. × 10 cm with a 15 μm tip) using gradient elution with HPLC buffer B (99.9% acetonitrile/0.1% formic acid) for 70 min at a flow rate of 0.3 μL/min. The eluted peptides were analyzed using a 2D linear ion trap mass spectrometer (Orbitrap Elite ETD; Thermo Fisher) operated with the Xcalibur 2.2 software (Thermo Fisher). Full-scan spectra were acquired over a mass range of 400–2,000 Da at a resolution of 120,000 at m/z 400. The ion signal of protonated dodecamethylcyclohexasiloxane at m/z 536.165365 was used as the lock mass for internal calibration. Precursor ions were isolated using a dynamic exclusion window (DEW) of 40 s with a relative mass window of 15 ppm. The 20 most abundant precursor ions were selected for fragmentation using data-dependent MS/MS scans following each MS scan. The electrospray voltage was set to 2.0 kV, and the capillary temperature was maintained at 200°C. The automatic gain control was set to 1,000 ms for full scans and 200 ms for MS/MS, with maximum accumulated ions of 3 × 106 for full scans and 3,000 ions for MS/MS.

#### Enzyme-linked immunosorbent assay (ELISA)

The concentrations of secreted TNF-α and IFN-β in each supernatant were measured using enzyme-linked immunosorbent assay (ELISA) kits (Quantikine ELISA, R&D Systems, Bio-Techne, Cat# DTA00D for TNF-α and Cat# DIFNB0 for IFN-β). To detect IFN-β in the human serum, we used an ELISA kit from Abcam (Cat# ab278127). Calibrator dilutions and sample preparation were performed according to the manufacturer’s instructions.

#### Immunohistochemistry (IHC)

Immunohistochemistry (IHC) was performed using the Novolink Polymer Detection System Kit (Leica Biosystems, Cat# RE7150-K) according to the manufacturer’s protocol. Briefly, formalin-fixed, paraffin-embedded (FFPE) tumor sections (5 μm thick) were deparaffinized, rehydrated, and subjected to antigen retrieval. For STAT1 and Lys637-acetylated STAT1, antigen retrieval was performed by autoclaving the slides in sodium citrate solution (pH 6.0) for 10 min. After washing with PBS-T (0.05% Tween 20) three times, the specimens were treated with peroxidase and protein blocking reagents (Leica Biosystems) before overnight incubation with primary antibodies (1:2000 for STAT1, CST Cat# 14994; 1:2000 for Lys637-acetylated STAT1) at 4°C. The following day, the tissues were incubated with horseradish peroxidase (HRP)-conjugated polymer for 30 min at room temperature, followed by diaminobenzidine (DAB) development and counterstaining with Mayer’s hematoxylin. Images were captured at 20× magnification using an Olympus microscope system (Olympus BX51; Olympus Corp., Japan). Staining scores for STAT1 and STAT1 Lys637 acetylation were assessed on a scale of 0 (negative) to 3 (high). The percentage of positive tumor cells was categorized as follows: 0% = 0, 1–25% = 1, 26–50% = 2, 51–75% = 3, and >75% = 4. The final staining score was calculated by multiplying the staining intensity score by the percentage of positive tumor cells.

#### Multiplex immunofluorescence staining

The experiment was performed as previously described.^50^ Human HNSCC samples were stained using an Opal IHC Kit (Akoya Biosciences) with two distinct multicolor opal panels. Opal Panel Set #1: This panel identified tumor and lymphoid cells using DAPI, CD4 (Thermo, 1:20, Opal 520), 4-HNE (ab46545, 1:250, Opal 570), CD8a (Thermo, 1:50, Opal 480), PD-L1 (CST#13684, 1:100, Opal 620), PD1 (ab137132, 1:250, Opal 690), and PanCK (ab27988, 1:500, Opal 780). Opal Panel Set #2: This panel identified tumor and myeloid cells using DAPI, CD11c (Abcam, 1:500, Opal 520), CD33 (Ab269456, 1:200, Opal 570), 4-HNE (ab46545, 1:250, Opal 480), CD66b (BD555723, 1:500, Opal 540), PD-L1 (CST#13684, 1:100, Opal 620), CD11b (ab52478, 1:1000, Opal 650), CD68 (ab955, 1:100, Opal 690), and PanCK (ab27988, 1:500, Opal 780). For each round of staining, the epitope-retrieval tissue slides were washed twice with PBST, followed by blocking with a blocking/antibody diluent solution (Akoya #ARD1001EA) for 10 min at room temperature. The slides were incubated with the primary antibody overnight at 4°C, followed by incubation with an HRP-conjugated polymer secondary antibody for 10 min at room temperature. After washing twice with PBST, the slides were incubated with a single Opal fluorophore working solution (Opal 480, 520, 540, 570, 620, and 690 stock reagents) for 10 min for first-round Opal signal development. Following each round of staining, the primary antibody-HRP polymer-Opal complex was removed by heat-induced epitope retrieval (HIER) treatment to allow the binding of the next primary antibody. This process of staining, signal development, and complex removal was repeated until all opal fluorophores were applied. After staining, the tissue slides were mounted using Fluoroshield medium containing DAPI (Sigma, Aldrich, #F6057). The Vectra Polaris Automated Quantitative Pathology Imaging System (Akoya Biosciences) was used to scan multispectral data, which were then analyzed using Phenochart (1.0.12) software (Akoya Biosciences). Further image analysis, including cell density quantification, was performed using the inForm software (Ver. 2.6).

#### Electrophoretic mobility shift assay (EMSA)

This experiment was conducted following a previously described protocol.^51^ The STAT1 EMSA kit (GS-0043, Signosis, Santa Clara, CA, USA) was used to detect differences in the DNA-binding activity of wild-type (WT) and mutant STAT1. Briefly, nuclear extracts from U3A cells infected with either pFLAG-STAT1 or pFLAG-STAT1-637R were incubated with a biotin-labeled STAT1 binding probe. For the competition assay, sequential dilutions of unlabeled competitors (cold probes) were added to the labeled probes. The resulting protein/DNA complexes were separated by electrophoresis on a non-denaturing polyacrylamide gel. The gel was subsequently transferred to a nylon membrane and detected using streptavidin-HRP and chemiluminescent substrate. The shifted bands corresponding to the protein/DNA complexes were visualized in comparison with unbound dsDNA. Bands were visualized using either film exposure or chemiluminescent imaging.

#### Luciferase reporter assay

For luciferase reporter assays, 2fTGH-U3A cells were infected with pGreenFire1-STAT1 lentivirus. Transduced cells were seeded in 6-well plates at a density of 1 × 105 cells/well and incubated for 24 h. The cells were then transiently transfected with 450 ng of either pLv-STAT1(K637R) or pLv-STAT1(K637Q) using the T-Pro P-Fect Transfection Reagent (JT97-N005M) and incubated overnight. After transfection, the cells were washed with PBS and 100 μL of reporter lysis buffer (Promega, Madison, WI, USA, Cat# E3971) combined with protease inhibitors (Roche, Mannheim, Germany) was added. The cells were harvested by scraping and the lysate was collected into microtubes. The lysate was briefly centrifuged and the supernatant was transferred to a new tube. For the luciferase assay, 20 μL of cell lysate was mixed with 100 μL of Luciferase Assay Reagent, and luminescence was measured using a Multimode Microplate Reader TECAN SPARK (TECAN, Männedorf, Switzerland). Luciferase activity was normalized to the total protein concentration.

#### *In vitro* acetylation assay

The recombinant PCAF/KAT2B protein (Abcam, Cat# ab268695) was used to perform *in vitro* acetylation assays. Biotin-tagged peptides, encompassing the STAT1 K637 region (FHAVEPYTKKELSAVTFP), served as the substrate. A control peptide was synthesized where lysine was substituted with non-acetylatable arginine (FHAVEPYTKRELSAVTFP). The standard assay mixture comprised 1 μg of peptide, 25 ng of the PCAF HAT domain protein, 25 μM acetyl-CoA (Sigma-Aldrich, No. A2056), and 8 μL of HAT assay buffer (200 mM Tris-HCl, pH 8.0, 10% glycerol, 0.1 mM EDTA, 1 mM dithiothreitol). Reactions were incubated for 60 min. Subsequently, reaction products were immobilized on nitrocellulose membrane and immunoblotted with an anti-acetyl-lysine antibody.

#### Murine tumor model

To investigate the impact of Stat1 knockdown on the ICB response (Figures 3F and 3G), MOCL2-1 sublines expressing a doxycycline-inducible Stat1 shRNA or a control vector were inoculated subcutaneously into female C57BL/6J mice to establish tumors. Stat1 knockdown was induced in 6-week-old tumor-bearing mice by providing doxycycline (2 mg/mL) in drinking water, supplemented with 5% sucrose, starting 18 days after tumor inoculation. Additionally, the mice received intraperitoneal injections of 250 μg anti-PD-L1 antibody (Bio X Cell, Lebanon, NH, USA) or isotype IgG every 3 days, beginning 18 days after tumor cell injection and continuing until the 35th day. The mice were sacrificed 53 days after the tumor cell injection. Tumor volumes were measured regularly, and tumor weights were recorded after harvest. To examine the impact of STAT1 Lys637 acetylation on anti-PD-1 efficacy (Figures 7A–7C), MOCL2-1 sublines transduced with mouse shRNA against Stat1 3′UTR (TRCN0000235837) and co-expressing pLV-human STAT1 or pLV-humanSTAT1(K637R) or pLV-human STAT1(K637Q) were inoculated subcutaneously into female C57BL/6J mice. When the tumor volume reached 100 mm^3^, the mice received intraperitoneal treatment with either Isotype IgG or anti-PD-1 antibody (Bio X Cell, Lebanon, NH, USA) every 3 days for a total of 5 doses (150 μg/dose). Tumor volumes and mice body weights were measured regularly, and tumor weights were recorded after harvest.

### Quantification and statistical analysis

Numerical data are presented as mean ± standard deviation (S.D.). Continuous variables were compared between the two groups using a two-tailed independent Student’s *t* test. Categorical variables were analyzed using either the chi-squared test or Fisher’s exact test, depending on the sample size and expected frequency conditions. Statistical analyses were based on at least three independent biological replicates to ensure reliability of the findings. A significance level of *p* ≤ 0.05 was applied across all statistical tests, with significance levels indicated as follows: ∗*p* ≤ 0.05, ∗∗*p* ≤ 0.01, and ∗∗∗*p* ≤ 0.001.

Survival analysis was conducted using the Kaplan-Meier method, with group comparisons made using the log rank test. The results are presented with 95% confidence intervals (CI) for median overall survival and progression-free survival. Prognostic factors for OS were initially identified using a univariate Cox proportional hazard model. Factors with extremely low event counts were excluded from the univariate analysis to avoid statistical instability. Logistic regression analysis was performed to identify risk factors for mortality, with variables showing *p* ≤ 0.1 in the univariate analysis included in a multivariate model. A *p*-value ≤0.05 in the multivariate analysis was considered indicative of an independent prognostic factor.

The relationship between the H-score and treatment outcomes was evaluated using the Mann-Whitney U test, which is appropriate for comparing non-normally distributed data. The optimal predictive cutoff value for the H-score was determined by generating a ROC curve. The area under the curve (AUC) was calculated to assess the discriminatory power of the test, with AUC values between 0.7 and 0.8 considered acceptable and an AUC of 1.0 indicating a perfect test. The optimal cut-off value was identified at the point corresponding to the highest Youden index (calculated as sensitivity + specificity −1).

## Supplemental information

Document S1. Figures S1–S8 and Tables S1, S2, and S8–S14

Table S3. RNA sequencing of HNSCC cells with cetuximab treatment, related to Figure 2

Table S4. Mass spectrometry of HNSCC cells with cetuximab treatment Column legends are provided at the bottom, related to Figure 2

Table S5. List of signature genes for gene set enrichment analysis, related to Figure 2

Table S6. RNA sequencing of patients before and after cetuximab treatment, related to Figure 2

Table S7. RNA sequencing of parental and cetuximab-resistant HNSCC cells, related to Figure 2

Document S2. Article plus supplemental information
