# Supplementary material for: Therapeutic stress triggers tumor STAT1 acetylation to disarm immunotherapy
Source: Cell Rep Med. 2025 Nov 7;6(11):102448. doi: 10.1016/j.xcrm.2025.102448 (PMC12711663; doi:10.1016/j.xcrm.2025.102448)
Supplement: Document S1. Figures S1–S8 and Tables S1, S2, and S8–S14 [file mmc1.pdf]

Cell Reports Medicine, Volume *6*

Supplemental information

Therapeutic stress triggers tumor

STAT1 acetylation to disarm immunotherapy

Po-Hsien Chiu, Kuan-Chen Lai, Hung-Ling Wang, Yao-Wen Chang, Wen-Chi Wu, Tien-Hua Chen, Yu-Shuen Tsai, Jie-Hong Song, Nai-Yun Sun, Gar-Yang Chau, Wen-Liang Fang, Ju-Pei Chen, Hung-Ming Wang, Huai-Cheng Huang, Meng-Che Hsieh, Chun-Hung Hua, Ming-Yu Lien, Yi-Fang Chang, Hui-Ching Wang, Chih-Yen Chien, Tai-Lin Huang, Chen-Chi Wang, Yi-Chun Liu, Jo-Pai Chen, Wei-Chen Lu, Ching-Yi Yiu, Chien-Liang Lin, Pei-Jen Lou, Pen-Yuan Chu, Shao-Chun Wang, Mien-Chie Hung, and Muh-Hwa Yang

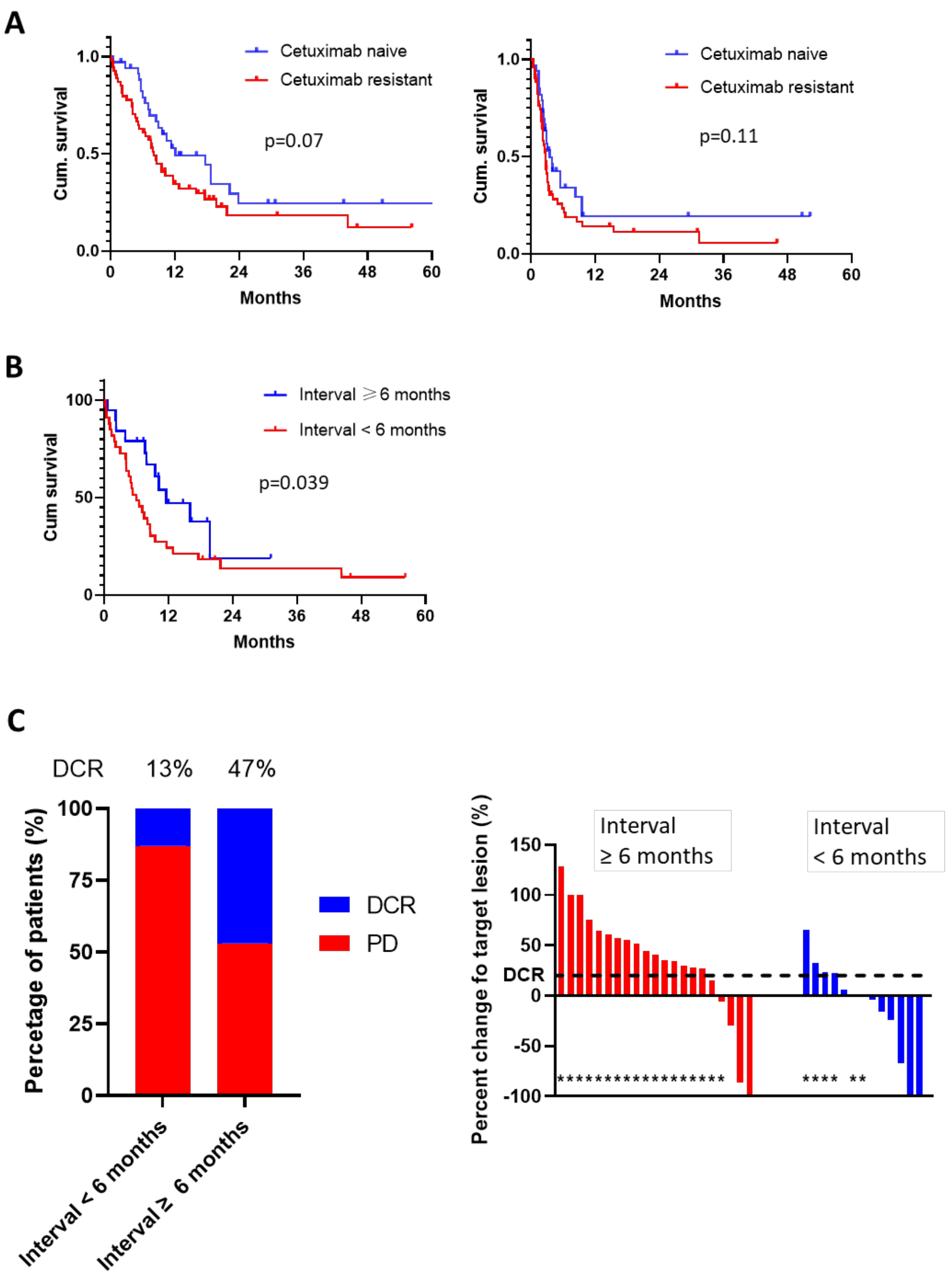

Figure S1. Survival analysis in cetuximab-naïve and cetuximab-resistant patient groups from the TVGH cohort. Related to Figure 1.

(A) Kaplan-Meier survival curves and log-rank tests for overall survival (left) and progression-free survival (right) from the start of immunotherapy in cetuximab-resistant (n=54) versus cetuximab-naïve (n=35) patients from the TVGH cohort.

(B) Kaplan-Meier curve and log-rank test for overall survival since the initiation of immunotherapy in patients with a cetuximab-ICB interval ≥ 6 months (n=19) versus < 6 months (n=33).

(C) Left: proportion of patients who achieved disease control with immunotherapy treatment in cetuximab-ICB interval ≥ 6 months (n=17) and <6 months (n=31) group from TVGH cohort. Blue: DCR. Red: PD. Right: percentage of change of measurable target lesions in cetuximab-ICB interval ≥ 6 months (n=13) and <6 months (n=21) group. Asterisk: PD as the best overall response.

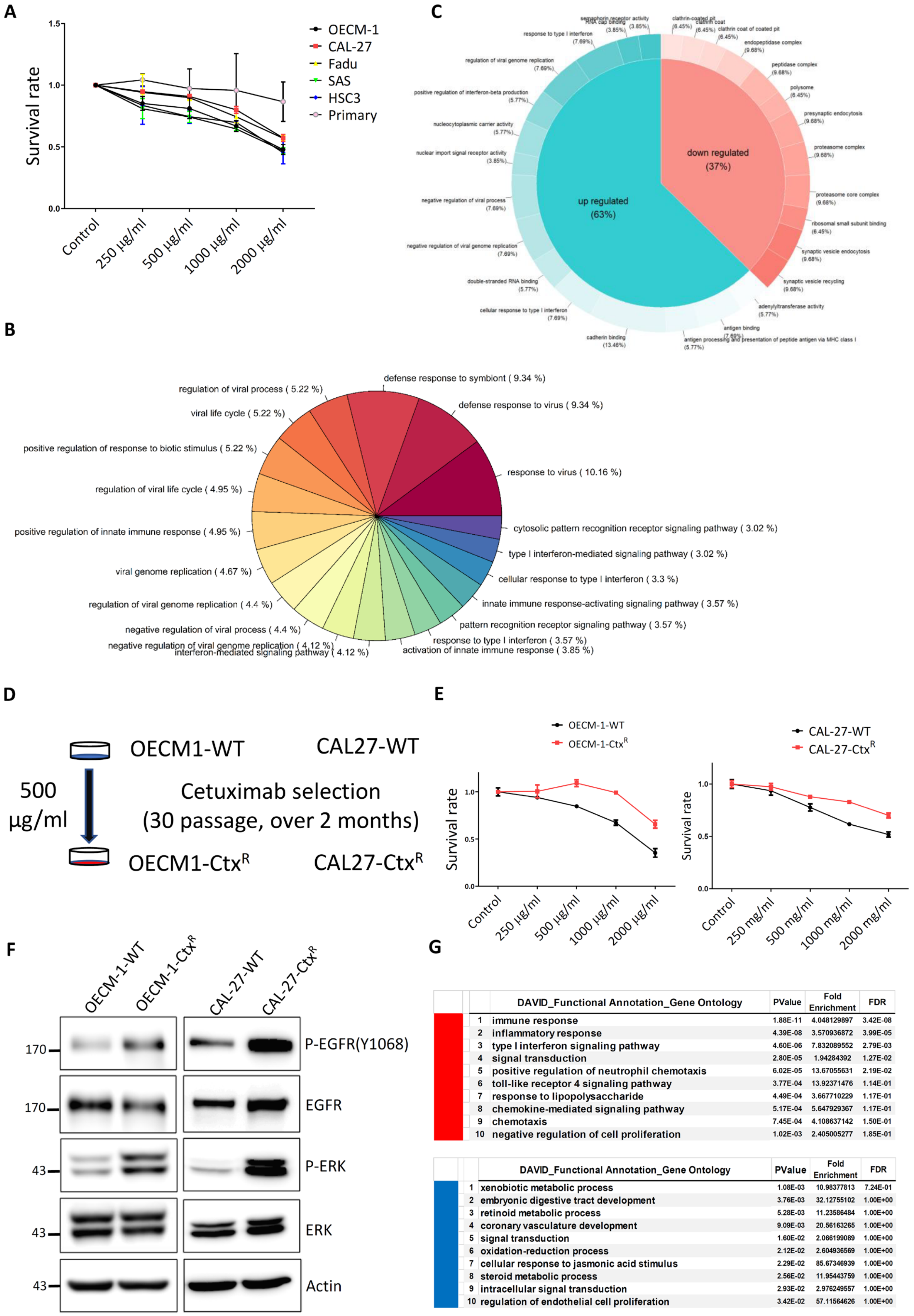

	DAVID_Functional Annotation_Gene Ontology	PValue	Fold Enrichment	FDR
1	immune response	1.88E-11	4.048129897	3.42E-08
2	inflammatory response	4.39E-08	3.570936872	3.99E-05
3	type I interferon signaling pathway	4.60E-06	7.832089552	2.79E-03
4	signal transduction	2.80E-05	1.94284392	1.27E-02
5	positive regulation of neutrophil chemotaxis	6.02E-05	13.67055631	2.19E-02
6	toll-like receptor 4 signaling pathway	3.77E-04	13.92371476	1.14E-01
7	response to lipopolysaccharide	4.49E-04	3.667710229	1.17E-01
8	chemokine-mediated signaling pathway	5.17E-04	5.647929367	1.17E-01
9	chemotaxis	7.45E-04	4.108637142	1.50E-01
10	negative regulation of cell proliferation	1.02E-03	2.405005277	1.85E-01

	DAVID_Functional Annotation_Gene Ontology	PValue	Fold Enrichment	FDR
1	xenobiotic metabolic process	1.08E-03	10.98377813	7.24E-01
2	embryonic digestive tract development	3.76E-03	32.12755102	1.00E+00
3	retinoid metabolic process	5.28E-03	11.23586484	1.00E+00
4	coronary vasculature development	9.09E-03	20.56163265	1.00E+00
5	signal transduction	1.60E-02	2.066199089	1.00E+00
6	oxidation-reduction process	2.12E-02	2.604936569	1.00E+00
7	cellular response to jasmonic acid stimulus	2.29E-02	85.67346939	1.00E+00
8	steroid metabolic process	2.56E-02	11.95443759	1.00E+00
9	intracellular signal transduction	2.93E-02	2.976249557	1.00E+00
10	regulation of endothelial cell proliferation	3.42E-02	57.11564626	1.00E+00

H

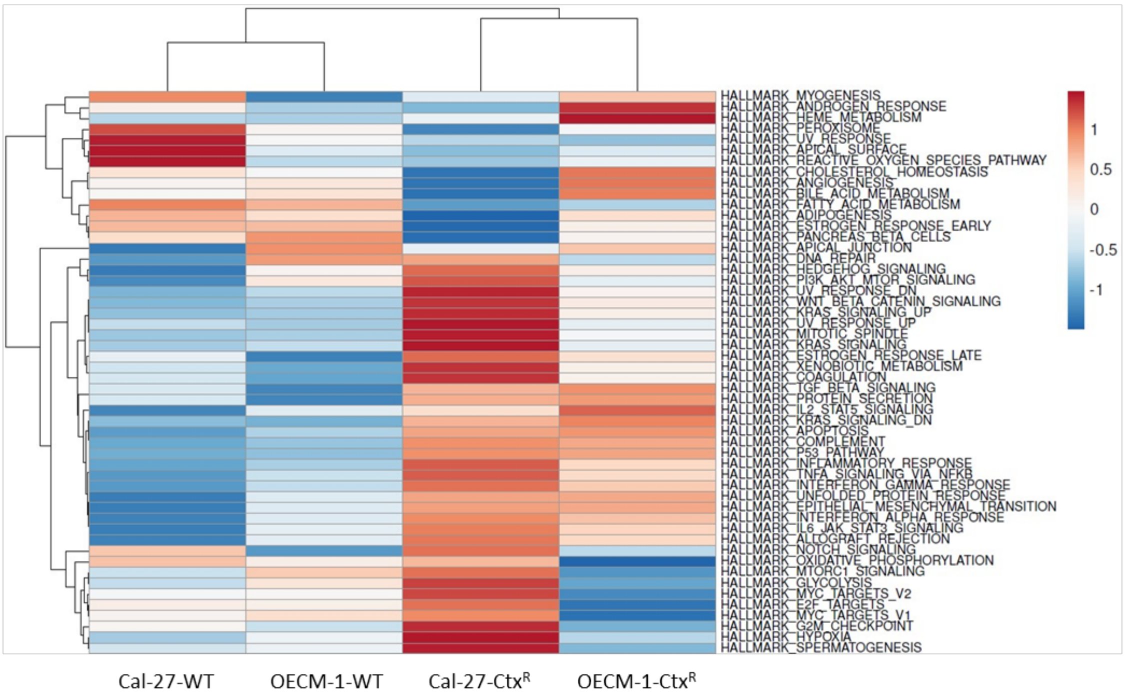
HALLMARK_MYOGENESIS
HALLMARK_ANDROGEN_RESPONSE
HALLMARK_HEME_METABOLISM
HALLMARK_PEROXISOME
HALLMARK_UV_RESPONSE
HALLMARK_APICAL_SURFACE
HALLMARK_REACTIVE_OXYGEN_SPECIES_PATHWAY
HALLMARK_CHOLESTEROL_HOMEOSTASIS
HALLMARK_ANGIOGENESIS
HALLMARK_BILE_ACID_METABOLISM
HALLMARK_FATTY_ACID_METABOLISM
HALLMARK_ADIPOGENESIS
HALLMARK_ESTROGEN_RESPONSE_EARLY
HALLMARK_PANCREAS_BETA_CELLS
HALLMARK_APICAL_JUNCTION
HALLMARK_DNA_REPAIR
HALLMARK_HEDGEHOG_SIGNALING
HALLMARK_PI3K_AKT_MTOR_SIGNALING
HALLMARK_UV_RESPONSE_DN
HALLMARK_WNT_BETA_CATENIN_SIGNALING
HALLMARK_KRAS_SIGNALING_UP
HALLMARK_UV_RESPONSE_UP
HALLMARK_MITOTIC_SPINDLE
HALLMARK_KRAS_SIGNALING
HALLMARK_ESTROGEN_RESPONSE_LATE
HALLMARK_XENOBIOTIC_METABOLISM
HALLMARK_COAGULATION
HALLMARK_TGF_BETA_SIGNALING
HALLMARK_PROTEIN_SECRETION
HALLMARK_IL2_STAT5_SIGNALING
HALLMARK_KRAS_SIGNALING_DN
HALLMARK_APOPTOSIS
HALLMARK_COMPLEMENT
HALLMARK_P53_PATHWAY
HALLMARK_INFLAMMATORY_RESPONSE
HALLMARK_TNFA_SIGNALING_VIA_NFKB
HALLMARK_INTERFERON_GAMMA_RESPONSE
HALLMARK_UNFOLDED_PROTEIN_RESPONSE
HALLMARK_EPITHELIAL_MESENCHYMAL_TRANSITION
HALLMARK_INTERFERON_ALPHA_RESPONSE
HALLMARK_IL6_JAK_STAT3_SIGNALING
HALLMARK_ALLOGRAFT_REJECTION
HALLMARK_NOTCH_SIGNALING
HALLMARK_OXIDATIVE_PHOSPHORYLATION
HALLMARK_MTORC1_SIGNALING
HALLMARK_GLYCOLYSIS
HALLMARK_MYC_TARGETS_V2
HALLMARK_E2F_TARGETS
HALLMARK_MYC_TARGETS_V1
HALLMARK_G2M_CHECKPOINT
HALLMARK_HYPOXIA
HALLMARK_SPERMATOGENESIS
1
0.5
0
-0.5
-1
Cal-27-WT
OECM-1-WT
Cal-27-Ctx^R
OECM-1-Ctx^R

I

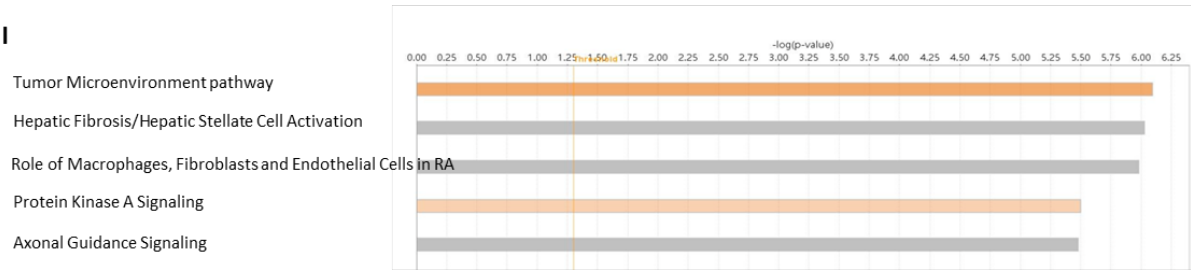
Tumor Microenvironment pathway
Hepatic Fibrosis/Hepatic Stellate Cell Activation
Role of Macrophages, Fibroblasts and Endothelial Cells in RA
Protein Kinase A Signaling
Axonal Guidance Signaling
-log(p-value)
0.00 0.25 0.50 0.75 1.00 1.25 1.50 1.75 2.00 2.25 2.50 2.75 3.00 3.25 3.50 3.75 4.00 4.25 4.50 4.75 5.00 5.25 5.50 5.75 6.00 6.25

J

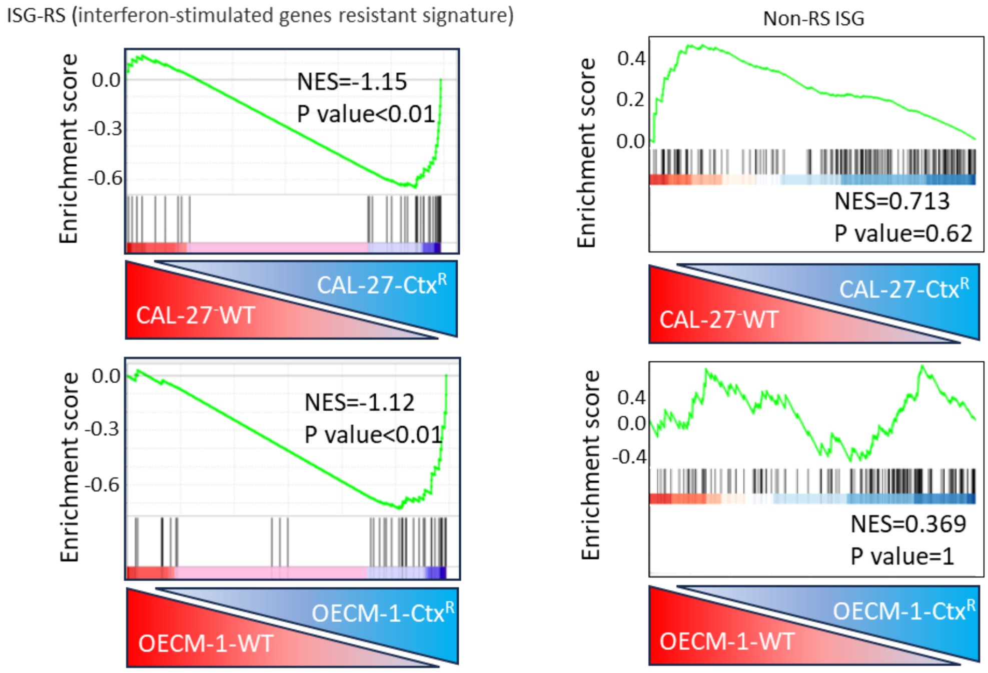
ISG-RS (interferon-stimulated genes resistant signature)
Non-RS ISG
Enrichment score
0.0
-0.3
-0.6
NES=-1.15
P value<0.01
CAL-27-Ctx^R
CAL-27 WT
0.4
0.2
0.0
NES=0.713
P value=0.62
NES=-1.12
P value<0.01
OECM-1-Ctx^R
OECM-1-WT
-0.4
NES=0.369
P value=1

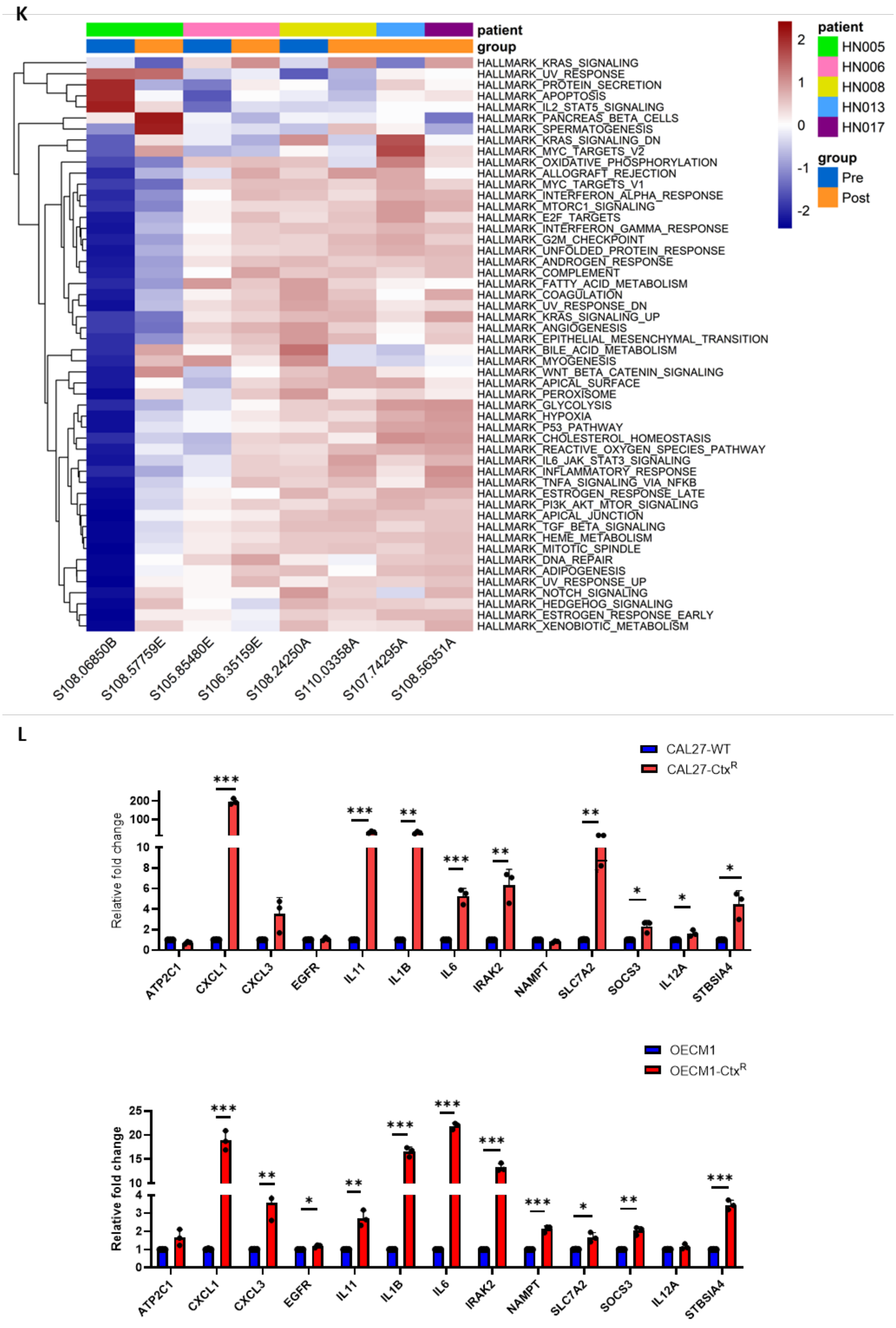

Figure S2. Proteogenomic analysis of HNSCC cells with prolonged cetuximab exposure. Related to Figure 2.

(A) Cell viability assay of HNSCC cell lines treated with the indicated cetuximab concentrations for 24 hours. Data represents mean ± SD (n=3).

(B) Gene ontology analysis of RNA sequencing results from OECM-1 cells treated with cetuximab (500 μg/ml) at the 8th passage (p8) versus parental cells (p0).

(C) Gene ontology analysis of mass spectrometry results from OECM-1 cells treated with cetuximab (500 μg/ml) at the 8th passage (p8) versus parental cells (p0).

(D) Schematic representation of cetuximab-resistant HNSCC sublines generation.

(E) Cell viability of OECM-1-WT/Ctx^R (left) and CAL-27-WT/Ctx^R (right) cells treated with the indicated cetuximab concentrations for 24 hours (n=2).

(F) Representative western blot analysis of the indicated protein expressions in OECM-1-WT/Ctx^R (left) and CAL-27-WT/Ctx^R (right) cells. β-Actin was used as the loading control. Experiments were performed in triplicate.

(G) Gene ontology analysis of differentially expressed genes in cetuximab-resistant sublines compared to parental cells.

(H) Single-sample Gene Set Enrichment Analysis (ssGSEA) of RNA sequencing data from OECM-1-WT/Ctx^R and CAL-27-WT/Ctx^R cells.

(I) Ingenuity Pathway Analysis (IPA) of canonical pathways in OECM-1-WT/Ctx^R and CAL-27-WT/Ctx^R cells.

(J) Gene Set Enrichment Analysis (GSEA) for interferon-stimulated gene resistance signature (ISG-RS; left) and non-resistance signature interferon-stimulated gene (non-RS ISG; right) in CAL-27-WT/Ctx^R (upper) and OECM-1-WT/Ctx^R (lower) cells. Normalized Enrichment Score (NES) and p-value are shown.

(K) ssGSEA of RNA sequencing data from patient samples before and after cetuximab treatment.

(L) qRT-PCR analysis examining the individual gene expression from cetuximab resistant signature (n=3). Data presents in mean ± SD. Statistical analyses were performed using an unpaired Student's t-test. *$p<0.05$, **$p<0.01$, ***$p<0.001$.

A

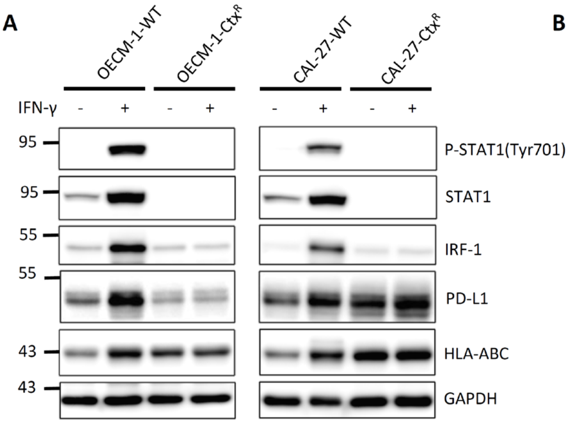

B

OECM-1-WT and OECM-1-Ctx^R

	misssense	stop_gained	start_lost	frameshift
IFNGR1	ND	ND	ND	ND
IFNGR2	ND	ND	ND	ND
JAK1	ND	ND	ND	ND
JAK2	ND	ND	ND	ND
STAT1	ND	ND	ND	ND

CAL-27-WT and CAL-27-Ctx^R

	misssense	stop_gained	start_lost	frameshift
IFNGR1	ND	ND	ND	ND
IFNGR2	ND	ND	ND	ND
JAK1	ND	ND	ND	ND
JAK2	ND	ND	ND	ND
STAT1	ND	ND	ND	ND

C

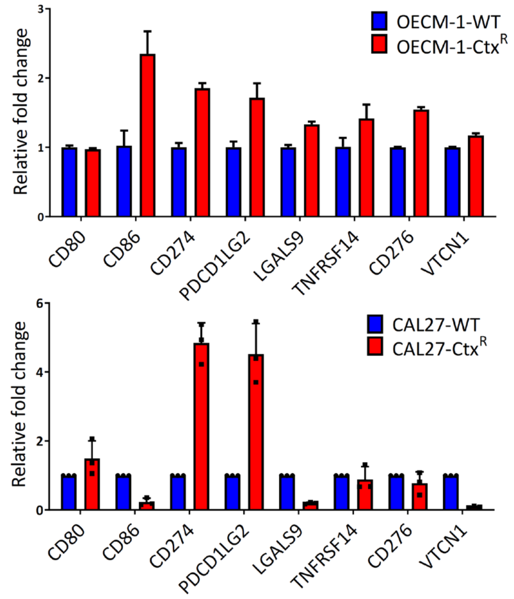

D

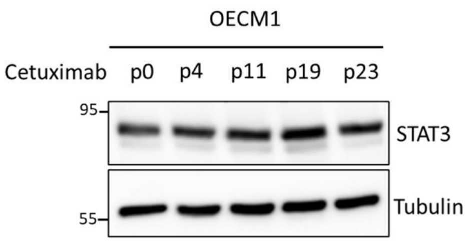

E

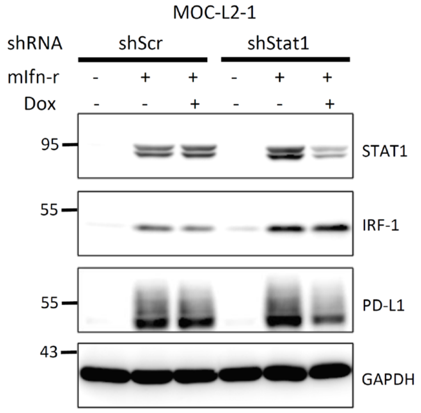

Figure S3. Impaired IFN-γ response in cetuximab-resistant sublines. Related to Figure 3.

(A) Representative western blot analysis of the indicated protein levels in OECM1-WT/Ctx^R and CAL27-WT/Ctx^R cells treated with IFN-γ (100 ng/ml) for 24 hours. GAPDH was used as the loading control. Experiments were performed in triplicate.

(B) A part of the whole exome sequencing results of the IFN-γ axis related genes for OECM1-WT/Ctx^R (upper) and CAL27-WT/Ctx^R (lower) cells.

(C) qRT-PCR analysis examining the expression of immune checkpoint ligands in OECM1-WT/Ctx^R (upper) and CAL27-WT/Ctx^R (lower) cells (n=3).

(D) Representative western blot analysis of STAT3 protein level in OECM-1 cells after cetuximab treatment (500 μg/ml) across different passages. Tubulin was a loading control.

(E) Representative western blot analysis of the indicated protein levels in MOCL2-1 cells transduced with shScramble or shStat1, treated with murine IFN-γ (100 ng/ml) and doxycycline (2 μg/ml) for 24 hours. GAPDH was used as the loading control. Experiments were performed in triplicate.

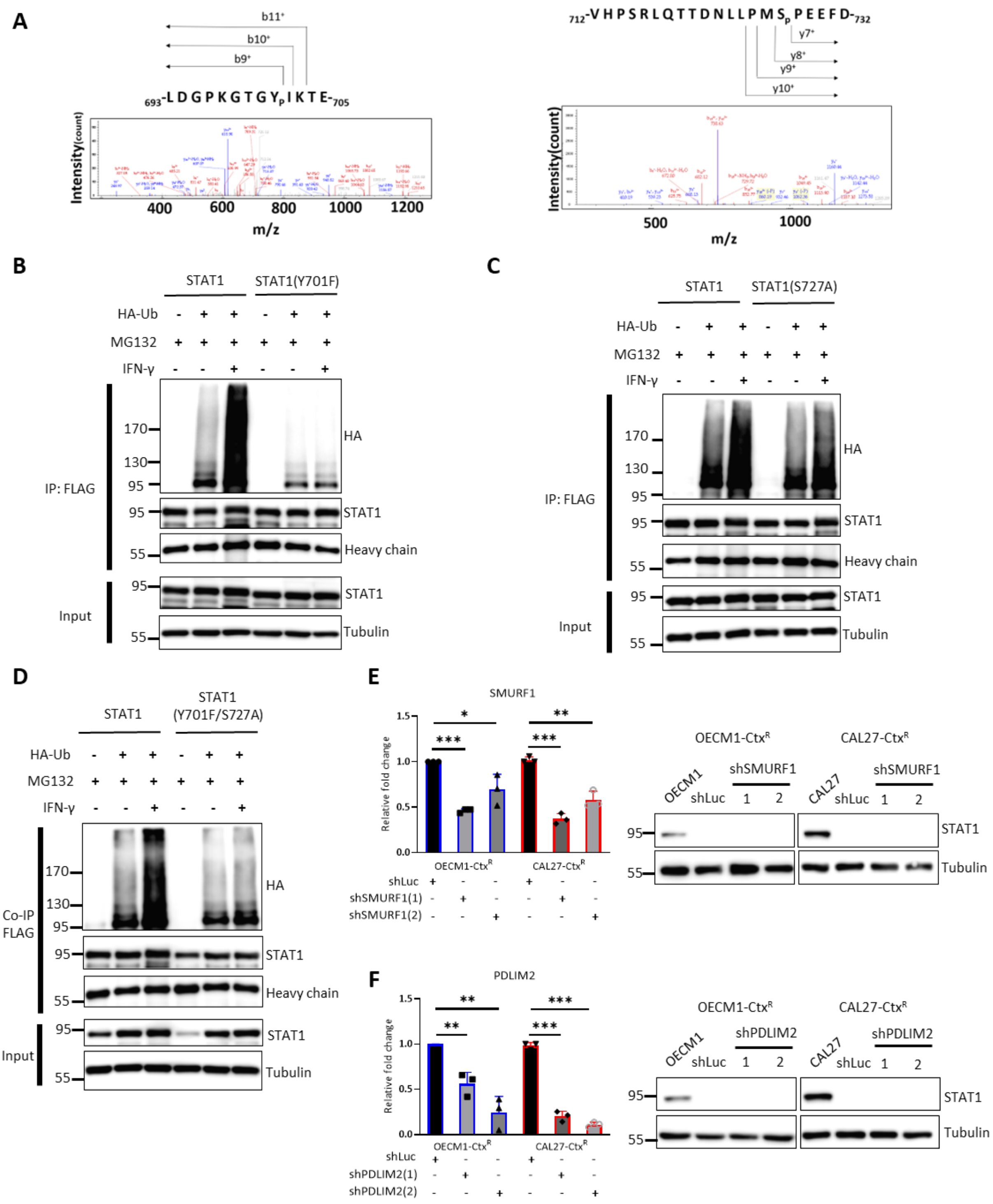

Figure S4. Phosphorylation and polyubiquitination of STAT1 in cetuximab-resistant HNSCC cells and IFN-γ treated cells. Related to Figure 4.

(A) Mass spectrometry analysis showing STAT1 phosphorylation at Tyrosine 701 (left) and Serine 727 (right).

(B) Representative western blot analysis of STAT1 polyubiquitination in 293T cells transduced with wild-type STAT1 and the Tyr701-unphosphorylatable mutant STAT1(Y701F). Cells were treated with MG132 (10 μM) and IFN-γ (100 ng/ml) for 30 minutes. α-Tubulin was used as the loading control in the input group. Experiments were performed in triplicate.

(C) Representative western blot analysis of STAT1 polyubiquitination in 293T cells transduced with wild-type STAT1 and the Ser727-unphosphorylatable mutant STAT1(S727A). Cells were treated with MG132 (10 μM) and IFN-γ (100 ng/ml) for 30 minutes. α-Tubulin was used as the loading

control in the input group. Experiments were performed in triplicate.

(D) Representative immunoprecipitation and western blot analysis of STAT1 polyubiquitination in 293T cells transduced with wild-type STAT1 and the double mutant STAT1(Y701F/S727A). Cells were treated with MG132 (10 μM) and IFN-γ (100 ng/ml) for 30 minutes. α-Tubulin was used as the loading control in the input group. Experiments were performed in triplicate.

(E) Left panel: qRT-PCR examining *SMURF1* level in OECM1-Ctx^R and CAL27-Ctx^R transduced with control shLuc or shSMURF1 shRNA (n=3). Data presents in mean ± SD. Statistical analyses were performed using an unpaired Student's t-test. *p<0.05, **p<0.01, ***p<0.001. Right panel: Representative western blot analysis of STAT1 level. α-tubulin was used as the loading control. Experiments were performed in triplicate.

(F) Left panel: qRT-PCR examining PDLIM2 level in OECM1-Ctx^R and CAL27-Ctx^R transduced with control shLuc or shPDLIM2 shRNA (n=3). Data presents in mean ± SD. Statistical analyses were performed using an unpaired Student's t-test. **p<0.01, ***p<0.001. Right panel: Representative western blot analysis of STAT1 level. α-tubulin was used as the loading control. Experiments were performed in triplicate.

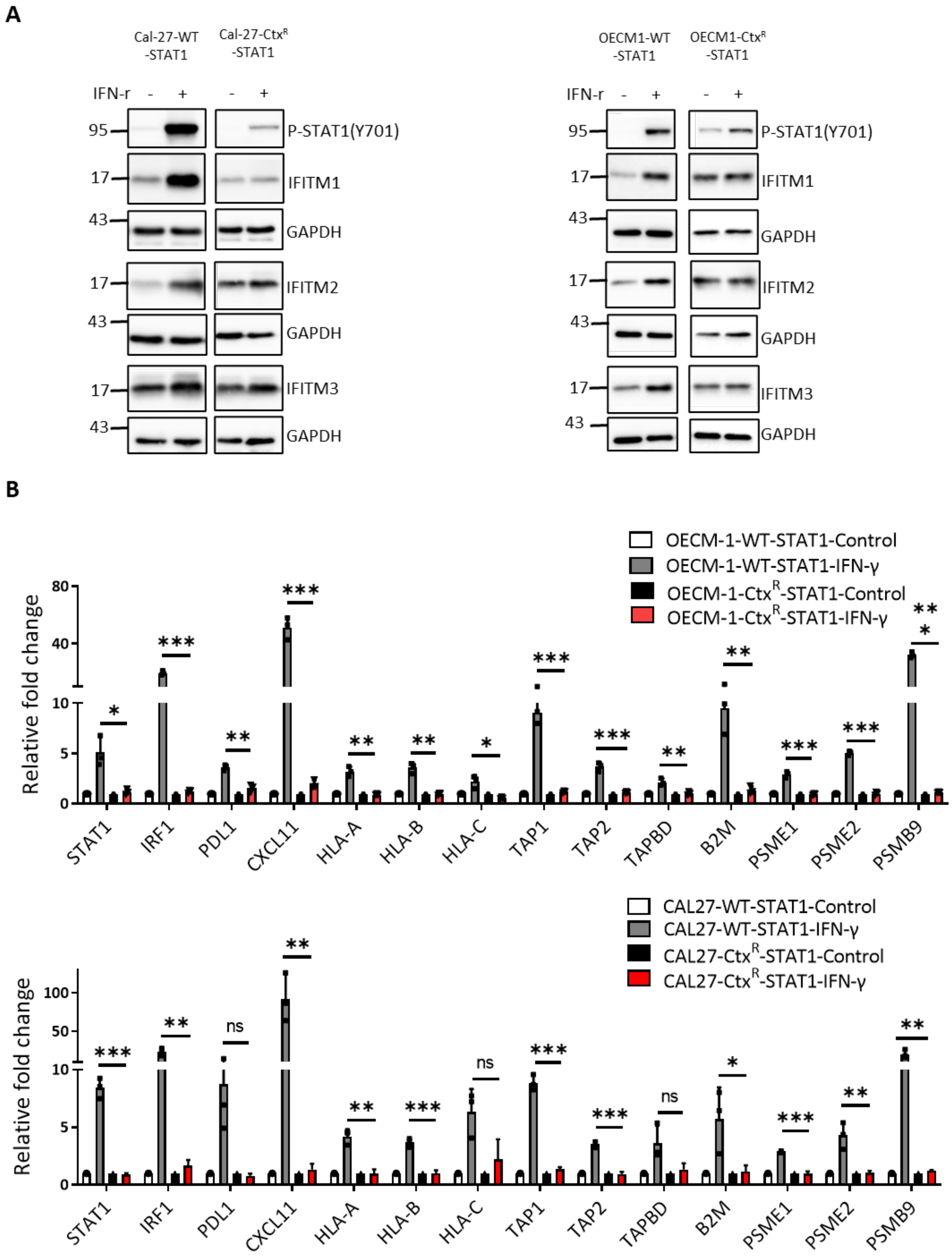
A
Cal-27-WT -STAT1
Cal-27-Ctx^R -STAT1
OECM1-WT -STAT1
OECM1-Ctx^R -STAT1
IFN-r - + - +
95
17
43
P-STAT1(Y701)
IFITM1
GAPDH
IFITM2
IFITM3
B
OECM-1-WT-STAT1-Control
OECM-1-WT-STAT1-IFN-γ
OECM-1-Ctx^R-STAT1-Control
OECM-1-Ctx^R-STAT1-IFN-γ
Relative fold change
STAT1
IRF1
PDL1
CXCL11
HLA-A
HLA-B
HLA-C
TAP1
TAP2
TAPBD
B2M
PSME1
PSME2
PSMB9
CAL27-WT-STAT1-Control
CAL27-WT-STAT1-IFN-γ
CAL27-Ctx^R-STAT1-Control
CAL27-Ctx^R-STAT1-IFN-γ
ns

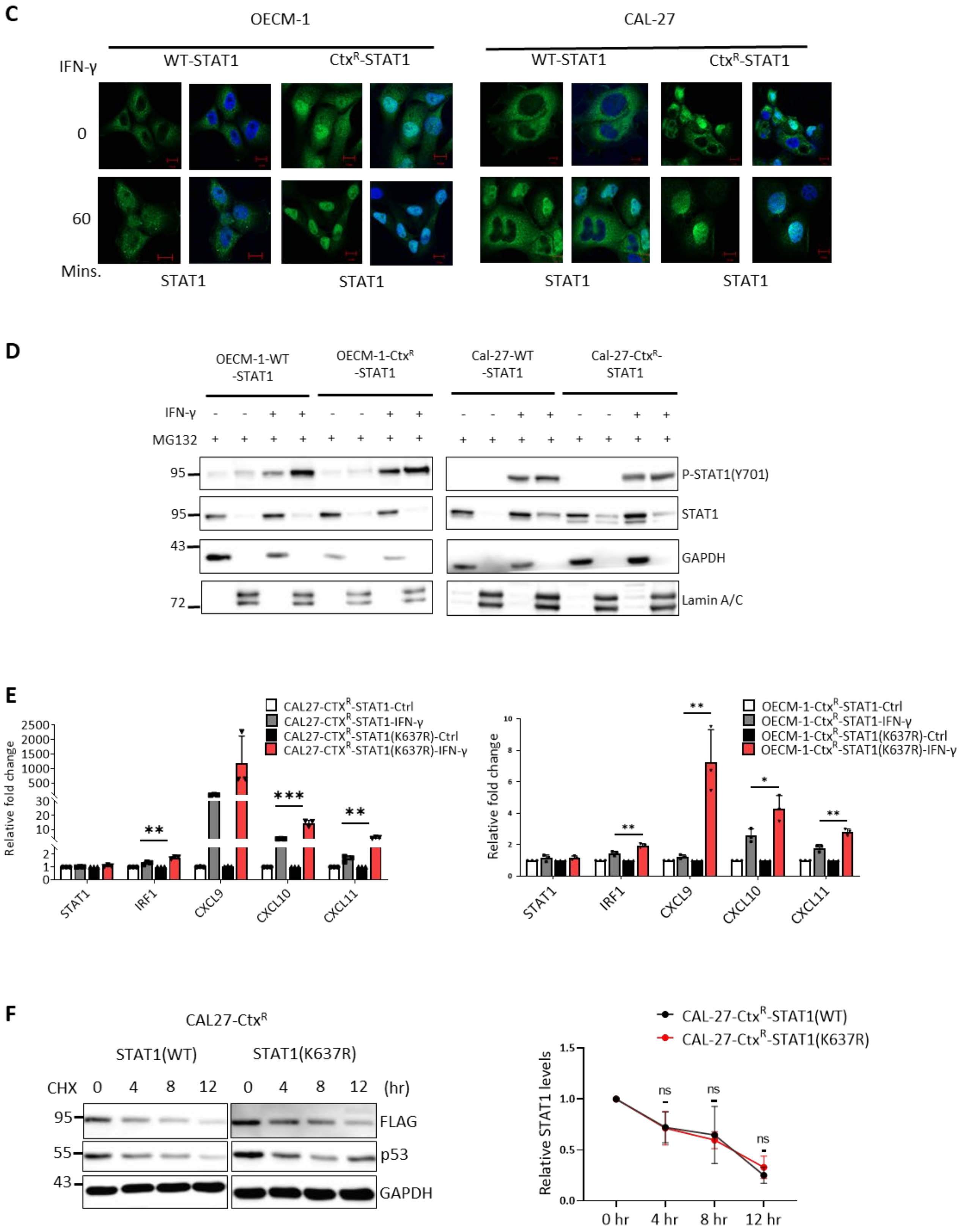
C
OECM-1
CAL-27
IFN-γ
WT-STAT1
CtxR-STAT1
0
60
Mins.
STAT1
D
OECM-1-WT -STAT1
OECM-1-CtxR -STAT1
Cal-27-WT -STAT1
Cal-27-CtxR- STAT1
IFN-γ
MG132
95
43
72
P-STAT1(Y701)
STAT1
GAPDH
Lamin A/C
E
CAL27-CTXR-STAT1-Ctrl
CAL27-CTXR-STAT1-IFN-γ
CAL27-CTXR-STAT1(K637R)-Ctrl
CAL27-CTXR-STAT1(K637R)-IFN-γ
Relative fold change
STAT1
IRF1
CXCL9
CXCL10
CXCL11
OECM-1-CtxR-STAT1-Ctrl
OECM-1-CtxR-STAT1-IFN-γ
OECM-1-CtxR-STAT1(K637R)-Ctrl
OECM-1-CtxR-STAT1(K637R)-IFN-γ
F
CAL27-CtxR
STAT1(WT)
STAT1(K637R)
CHX 0 4 8 12 (hr)
95
55
43
FLAG
p53
GAPDH
CAL-27-CtxR-STAT1(WT)
CAL-27-CtxR-STAT1(K637R)
Relative STAT1 levels
ns
0 hr
4 hr
8 hr
12 hr

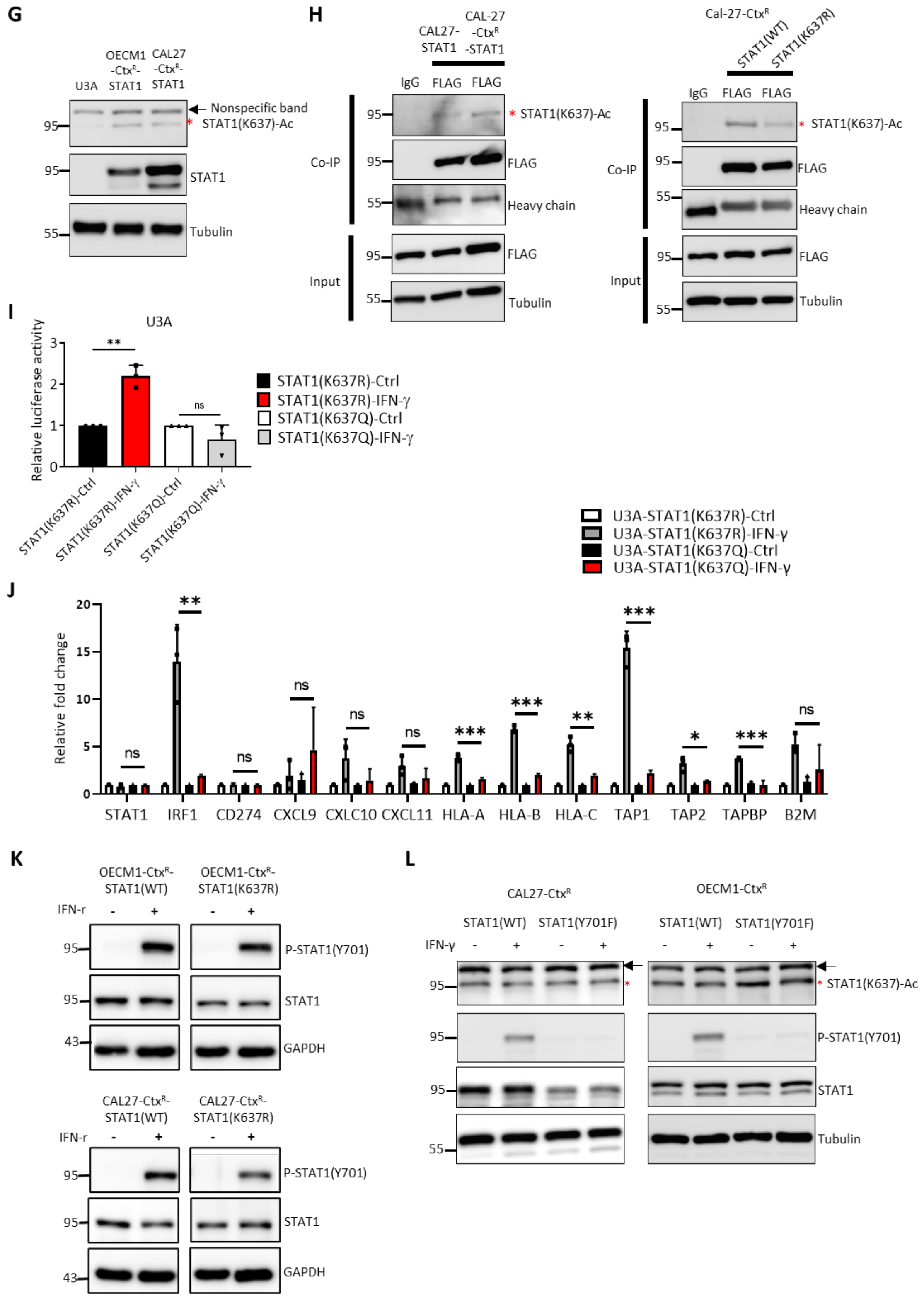

Figure S5. Lys637 acetylation reduces STAT1 transcriptional activity. Related to Figure 5.

(A) Representative western blot analysis of IFITM1, IFITM2 and IFITM3 in CAL27-WT/CAL27-Ctx^{R} (left) or OECM1-WT/OECM1-Ctx^{R} transduced with STAT1 (CAL27-WT-STAT1/CAL27-Ctx^{R}-STAT1 and OECM1-WT-STAT1/OECM1-Ctx^{R}-STAT1) treat with IFN-γ (100 ng/ml) for 24 hrs. GAPDH was used as the loading control. Experiments were performed in triplicate.

(B) qRT-PCR analysis of IFN-γ response-associated gene expression in OECM1-WT/Ctx^R (upper) and CAL27-WT/Ctx^R (lower) cells transfected with STAT1 and treated with IFN-γ (100 ng/ml) for 24 hours. n=3 (each with two technical replicates). Data presents in mean ± SD. Statistical analyses were performed using an unpaired Student's t-test. *p<0.05, **p<0.01, ***p<0.001, ns = not significant.

(C) Immunofluorescence analysis of STAT1 protein localization upon IFN-γ (100 ng/ml) stimulation for 60 minutes in OECM1-WT/Ctx^R (left) and CAL27-WT/Ctx^R (right) cells transfected with STAT1. Scale bar: 10 μm.

(D) Representative western blot analysis of p-STAT1(Y701) localization by nucleus/cytoplasm fractionation assay. GAPDH was used as cytoplasmic loading control and lamin A/C was used as nucleus loading control. Experiments were performed in triplicate.

(E) qRT-PCR analysis of IFN-γ response-associated gene expression in CAL27-Ctx^R (left) and OECM1-Ctx^R (right) cells transfected with wild-type STAT1 or STAT1(K637R), treated with IFN-γ (100 ng/ml) for 24 hours. n=3 (each with two technical replicates). Data presents in mean ± SD. Statistical analyses were performed using an unpaired Student's t-test. *p<0.05, **p<0.01, ***p<0.001.

(F) Pulse-chase assay for STAT1 protein stability. Left: Representative western blot analysis of wild-type STAT1 and STAT1(K637R) protein degradation under cycloheximide (20 μg/ml) treatment for the indicated times in CAL27-Ctx^R cells. GAPDH was used as the loading control. Right: Quantification of the results. n=3. Data display mean ± SD. Statistical analyses were performed using an unpaired Student's t-test. ns = not significant.

(G) Western blot analysis of the STAT1 Lys637 acetylation in STAT1-null U3A cell and CAL-27-Ctx^R, OECM1-Ctx^R cells transfected with STAT1 (CAL27-Ctx^R-STAT1 and OECM1-Ctx^R-STAT1). Star indicates the band of detected STAT1 Lys637 acetylation. Arrow indicates the non-specific binding of antibody. α-Tubulin was used as a loading control. The experiments were performed in triplicate.

(H) Co-immunoprecipitation and western blot analyses of STAT1 Lys637 acetylation using a STAT1 Lys637 acetylation-specific antibody in CAL27 and CAL27-Ctx^R cells transfected with wild type STAT1 (CAL27-STAT1(WT) and CAL27-Ctx^R-STAT1(WT) (left), and CAL27-Ctx^R transduced wild type or Lys637 mutated STAT1 (CAL27-CtxR-STAT1(WT) and CAL27-Ctx^R-STAT1(K637R)) (right) were treated with MG132 (10 μM) 16 h. α-Tubulin was used as a loading control.

(I) Luciferase reporter assay evaluating the transcriptional activity of STAT1(K637Q) and STAT1(K637R) mutants in STAT1-null U3A cells treated with IFN-γ (100 ng/ml) for 24 hours. n=3 (each with two technical replicates). Data display mean ± SD. Statistical analyses were performed using an unpaired Student's t-test. **p<0.01, ns = not significant.

(J) qRT-PCR analysis of IFN-γ response-associated gene expression in U3A cells transduced with STAT1(K637R) / STAT1(K637Q), treated with IFN-γ (100 ng/ml) for 24 hours. n=3 (each with two technical replicates). Data display mean ± SD. Statistical analyses were performed using an unpaired Student's t-test. *p<0.05, **p<0.01, ***p<0.001, ns = not significant.

(K) Representative western blot analysis of STAT1 phosphorylation (Tyr701) in OECM1-Ctx^R (upper) and CAL27-Ctx^R (lower) cells transduced with wild-type STAT1 or STAT1(K637R) with or without IFN-γ (100 ng/ml) treatment. GAPDH was used as the loading control. Experiments were performed in triplicate.

(L) Western blot analysis of STAT1 Lys637 acetylation in CAL27-Ctx^R (left) and OECM1-Ctx^R (right) cells transduced with wild-type STAT1 or STAT1(Y701F) with or without IFN-γ (100 ng/ml) treatment. α-tubulin was used as the loading control. Star indicates the band of detected STAT1 Lys637 acetylation. Arrow indicates the non-specific binding of antibody.

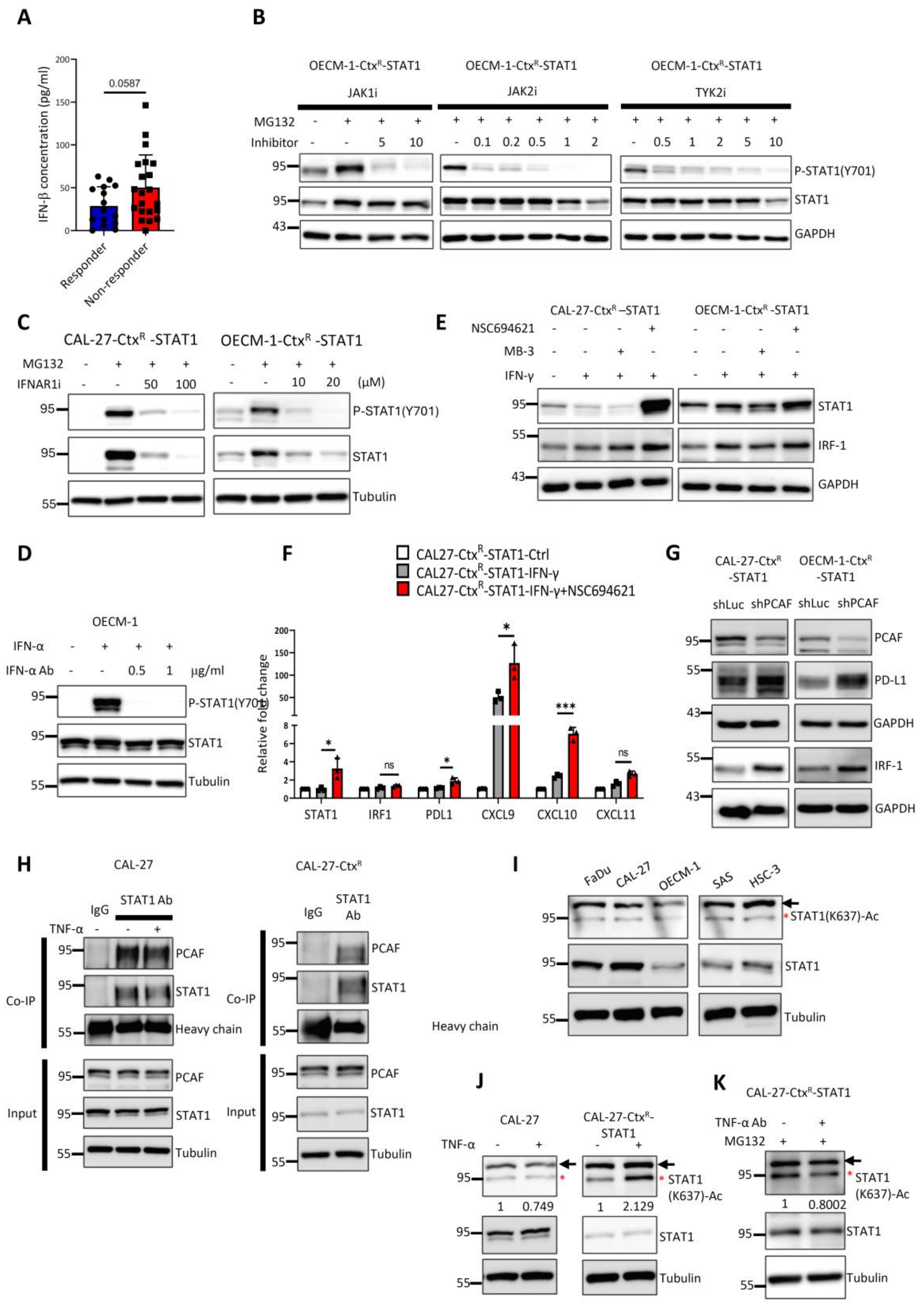

Figure S6. Upstream regulators of STAT1 inactivation in cetuximab-resistant HNSCC. Related to Figure 6.

(A) ELISA of IFN-β levels in sera from HNSCC patients receiving ICB treatment, with (n=15) or without (n=21) a response to ICB. The histogram shows mean ± SD. Statistical analyses were performed using an unpaired Student's t-test.

(B) Representative western blot analysis of STAT1 Tyr701 phosphorylation in OECM-1-Ctx^R-STAT1 cells treated with MG132 (20 μM) combined with JAK1, JAK2, or TYK2 inhibitors (μM) for 16 h at the indicated concentrations. GAPDH was used as the loading control. Experiments were performed in triplicate.

(C) Representative western blot analysis of STAT1 Tyr701 phosphorylation in CAL27-Ctx^R (left) and OECM1-Ctx^R (right) cells transfected with STAT1 (CAL27-Ctx^R-STAT1 and OECM1-Ctx^R-STAT1) and treated with MG132 (10 μM) combined with a IFNAR1 inhibitor at the indicated concentrations for 16 hours. GAPDH was used as the loading control. Experiments were performed in triplicate.

(D) Representative western blot analysis of STAT1 Tyr701 phosphorylation in OECM-1 cells treated with IFN-α (10 ng/ml) combined with an IFN-α neutralizing antibody for 30 minutes at the indicated concentrations. α-Tubulin was used as the loading control. Experiments were duplicated.

(E) Representative western blot analysis of the IFN-γ response in CAL-27-Ctx^R and OECM1-Ctx^R cells transfected with STAT1 (CAL27-Ctx^R-STAT1 and OECM1-Ctx^R-STAT1) and treated with IFN-γ (100 ng/ml) combined with a GCN5 inhibitor MB-3 (400 μM) or NSC694621 (20 μM). GAPDH was used as a loading control. The experiments were performed in triplicate.

(F) qRT-PCR analysis of IFN-γ response-associated gene expression in CAL27-Ctx^R cells transfected with STAT1 (CAL27-Ctx^R-STAT1) and treated with or without IFN-γ (100 ng/ml) and a PCAF inhibitor NSC694621 (20 μM) treatment for 24 hours. n=3 (each with two technical replicates). Data are presented as mean ± SD. Statistical analyses were performed using an unpaired Student's t-test. *$p<0.05$, ***$p<0.001$, ns = not significant.

(G) Representative western blot analysis of PCAF knockdown experiment in CAL27-Ctx^R and OECM1-Ctx^R transduced with wild-type STAT1 (CAL27-Ctx^R-STAT1 and OECM1-Ctx^R-STAT1) and infected with lentivirus composed of shLuc or shPCAF shRNA. GAPDH was used as loading control. The experiments were performed in triplicate.

(H) Immunoprecipitation and western blot analysis for detecting STAT1 and PCAF interaction in CAL27 cells treat with or without TNF-α (20 ng/ml) for 24 h (left). STAT1 and PCAF interaction in CAL27-Ctx^R cells treat with MG132 (20 μM) for 16 h (right). α-tubulin was used as loading control.

(I) Western blot analysis of STAT1 Lys637 acetylation in five wild-type HNSCC cell lines (FaDu, CAL-27, OECM-1, SAS, HSC3). GAPDH was a loading control. Star indicates the band of detected STAT1 Lys637 acetylation. Arrow indicates the non-specific binding of antibody.

(J) Western blot analysis of STAT1 Lys637 acetylation in CAL27 (left) and CAL27-Ctx^R-STAT1 (right) treat with TNF-α (20 ng/ml) for 24 hrs. α-tubulin was used as loading control. Star indicates the band of detected STAT1 Lys637 acetylation. Arrow indicates the non-specific binding of antibody. Quantification of Lys637 acetylated STAT1 is shown alongside the blots.

(K) Western blot analysis of CAL27-Ctx^R-STAT1 treat with MG132 (10 μM) combined with TNF-α neutralized antibody (200 ng/ml) for 16 h. α-tubulin was used as loading control. Star indicates the band of detected STAT1 Lys637 acetylation. Arrow indicates the non-specific binding of antibody. Quantification of Lys637 acetylated STAT1 is shown alongside the blots.

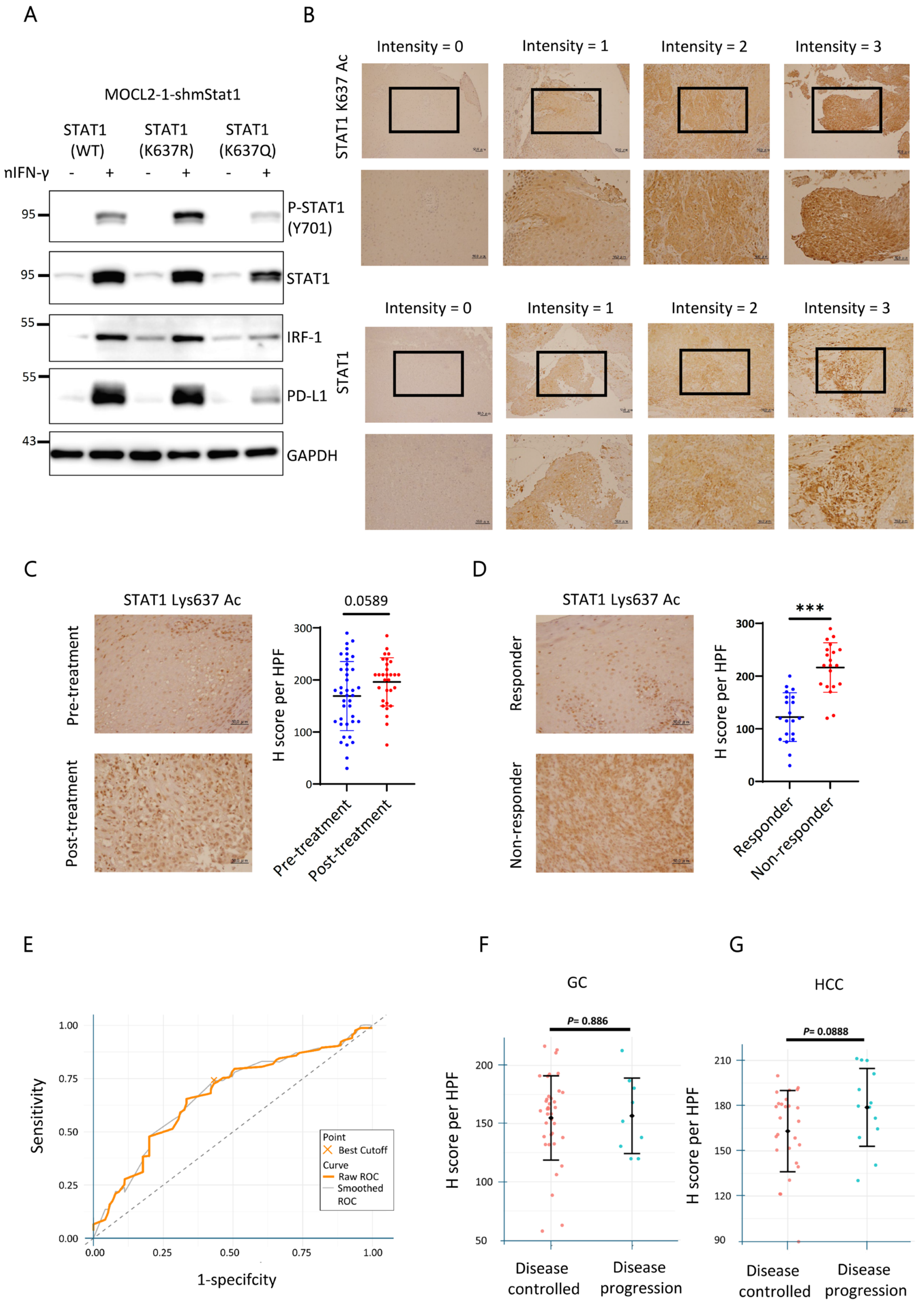

Figure S7. Clinical relevance of STAT1 Lys637 acetylation. Related to Figure 7.

(A) Representative western blot analysis of the indicated protein levels in the murine oral squamous cell carcinoma cell line MOC-L2-1 transduced with murine shRNA against mStat1 (targeting the 3'UTR) combined with human hSTAT1(WT), hSTAT1(K637R) or hSTAT1(K637Q) and treated with mIFN-γ (100 ng/ml) for 24 hours. GAPDH was used as the loading control. Experiments were performed in triplicate.

(B) Representative images of STAT1 Lys637 acetylation (upper) and STAT1 (lower) staining with varying intensities. Scale bar: 50 μm.

(C) Left: Representative IHC images of STAT1 Lys637 acetylation in HNSCC patients before (ROI=40) and after cetuximab treatment (ROI=30). Scale bar: 50 μm. Right: Quantification of IHC images showing STAT1 Lys637 acetylation before and after cetuximab treatment. Data are presented as mean ± SD. Statistical analyses were performed using an unpaired Student's t-test.

(D) Left: Representative IHC images of STAT1 Lys637 acetylation in HNSCC responder (ROI=20) and non-responder (ROI=20) groups to cetuximab treatment. Scale bar: 50 μm. Right: Quantification of IHC images in responder and non-responder groups to cetuximab treatment. Data are presented as mean ± SD. Statistical analyses were performed using an unpaired Student's t-test. ***$p<0.001$.

(E) Time-dependent reviewer operating characteristic curve (ROC) for 12-month overall survival prediction using H-score across the entire patient cohort ($n = 148$). The cutoff was labeled with x.

(F) Quantification of IHC images of STAT1 Lys637 acetylation in gastric cancer patients with disease control versus progression to ICB treatment. Data are presented as mean ± SD. Statistical analyses were performed using an unpaired Student's t-test.

(G) Quantification of IHC images of STAT1 Lys637 acetylation in HCC patients with disease control versus progression to ICB treatment. Data are presented as mean ± SD. Statistical analyses were performed using an unpaired Student's t-test.

Table S1. Patient characteristics and multivariate analysis of overall survival of THNS registry cohort. Related to Figure 1.

Table S1A. Patient characteristics of THNS# registry cohort. Related to Figure 1.

	Cetuximab naive (%) (n=60)	Cetuximab resistant (%) (n=104)	p-value
Age y/o (95% CI)	55.0 (52.3 – 57.6)	54.1 (50.3 – 57.9)	0.41
Sex			
Female	4 (6.7)	11 (10.6)	0.40
Male	56 (93.3)	93 (89.4)	
Primary location			
Oral cavity	41 (68.3)	67 (64.4)	0.33¶
Oropharynx	8 (13.3)	18 (17.3)	
Hypopharynx	10 (16.7)	10 (9.6)	
Larynx	1 (1.7)	6 (5.8)	
Others	0 (0.0)	3 (2.9)	
HPV-associated&			
Positive	3 (5.0)	6 (5.8)	0.88
Negative	10 (16.7)	20 (19.4)	
Clinical stage%			
Stage I-II	14 (23.3)	26 (25.0)	0.90
Stage III	7 (11.7)	10 (9.6)	
Stage IV	39 (65.0)	64 (61.5)	
Recurrence pattern$			
Locoregional recurrence only	13 (21.6)	49 (47.1)	0.002*
Distant metastasis	42 (70.0)	52 (50.0)	
Previous curative RT			
Yes	15 (25.0)	33 (31.7)	0.18¶
No	45 (75.0)	67 (64.4)	
NA	0 (0.0)	4 (3.8)	
Previous curative surgery			
Yes	39 (65.0)	75 (72.1)	0.34
No	21 (35.0)	29 (19.4)	
Platinum refractory			
Yes	28 (46.7)	46 (44.2)	0.76
No	32 (53.3)	58 (55.8)	

#Taiwan Head and Neck Society ¶ Fisher's exact test, *p<0.05, & Only 39 patients had HPV-PCR or p16-IHC staining, % 4 patients in cetuximab resistant group do not have recorded initial staging, $ 5 patients in cetuximab naïve group, and 3 patients in cetuximab resistant group do not have specified recurrent-only or distant metastasis status.

Table S1B. Multivariate analysis of overall survival in cetuximab-naïve versus cetuximab-resistant patients from THNS registry. Related to Figure 1.

	Reference	HR (95% CI)	p
Cetuximab naive	Cetuximab resistant	0.66 (0.44-1.00)	0.048
Male	Female	0.88 (0.46-1.58)	0.62
Age ≥ 65	Age < 65	1.04 (0.68-1.60)	0.87
Non-oral HNSCC	Oral cavity SCC	0.94 (0.61-1.46)	0.80
Stage III-IV	Stage I-II	0.82 (0.53-1.28)	0.38
Prior RT	No prior RT	1.20 (0.79-1.83)	0.40
Prior surgery	No prior surgery	1.15 (0.72-1.84)	0.56
Distant metastasis	Locoregional recurrence only	0.51 (0.07-3.97)	0.52

Table S2. Patient characteristics of TVGH cohort. Related to Figure 1.

	Cetuximab first (%) (n=54)	IO first (%) (n=35)	p-value
Age y/o (95% CI)	54.5 (54.2-60.8)	59.8 (55.1–64.6)	0.584
Sex			
Female	7 (13.0)	5 (14.3)	0.889¶
Male	47 (87.0)	30 (85.7)	
Primary location			
Oral cavity	22 (40.7)	23 (65.7)	0.069
Oropharynx	7 (13.0)	5 (14.3)	
Hypopharynx	18 (33.3)	3 (8.6)	
Larynx	5 (9.3)	2 (5.7)	
Others	2 (3.7)	2 (5.7)	
Clinical T stage			
T0	1 (1.8)	0 (0.0)	0.642¶
T1	4 (7.5)	4 (11.4)	
T2	14 (25.9)	7 (20.0)	
T3	10 (18.5)	5 (14.3)	
T4	22 (40.7)	14 (40.0)	
NA	3 (5.6)	5 (14.3)	
Clinical N stage			
N0	16 (29.6)	13 (37.1)	0.433¶
N1	9 (16.7)	2 (5.7)	
N2	23 (42.6)	13 (37.1)	
N3	3 (5.6)	3 (8.6)	
NA	3 (5.6)	4 (11.4)	
Clinical M stage			
M0	51 (94.4)	29 (82.9)	0.137¶
M1	2 (3.7)	2 (5.7)	
NA	1 (1.9)	4 (11.4)	
Previous definitive CCRT			
Yes	50 (92.6)	28 (80)	0.152¶
No	4 (7.4)	7 (20)	
Previous definitive surgery			
Yes	38 (70.4)	23 (65.7)	0.644

No	16 (29.6)	12 (34.3)	
Prior treatment lines			
0	16 (29.6)	8 (22.9)	0.165¶
1	28 (51.9)	13 (37.1)	
2	9 (16.7)	12 (34.3)	
3	1 (1.9)	2 (5.7)	
Platinum refractory			
Yes	39 (72.2)	14 (40.0)	0.002*
No	15 (27.8)	21 (60.0)	

¶ Fisher's exact test; *p<0.05

Table S8. List of Cetuximab resistant signature genes. Related to Figure 2.

Upregulated gene

gene name	gene id
ATP2C1	ENSG00000017260
CXCL1	ENSG00000163739
CXCL3	ENSG00000163734
EGFR	ENSG00000146648
IL11	ENSG00000095752
IL12A	ENSG00000168811
IL1B	ENSG00000125538
IL6	ENSG00000136244
IRAK2	ENSG00000134070
NAMPT	ENSG00000105835
SLC7A2	ENSG00000003989
SOCS3	ENSG00000184557
ST8SIA4	ENSG00000113532

Table S9. Characteristics of HNSCC, GC and HCC patients receiving ICB treatment and analyzed by STAT1 Lys637 acetylation. Related to Figure 7.

Table S9A. Characteristics of HNSCC patients receiving ICB treatment and analyzed by STAT1 Lys637 acetylation. Related to Figure 7.

	Total (n=63)	High H-score (≥166) n=38	Low H-score (<166) n=25	*P* value
Age (median, Q1-Q3)	56.6 (50.9-63.3)	56.9 (50.9-62.9)	56.5 (52.6-64.3)	0.70
Age (>65)	14 (22.2)	8 (21.1)	6 (24%)	0.78
Sex Male	56 (88.9)	35 (92.1)	21 (84)	0.421
Primary tumor site				0.64
Oral cavity	30 (47.6)	19 (50)	11 (44)	0.80
Oropharynx	17 (27.0)	11 (28.9)	6 (24)	0.59
Larynx	4 (6.3)	3 (7.9)	1 (4)	
Hypopharynx	8 (12.7)	3 (7.9)	5 (20)	
Others	4 (6.3)	2 (5.3)	2 (8)	
Treatment				
Types of immunotherapy				0.54
Pembrolizumab	49 (77.8)	31 (81.6)	18 (72)	
Nivolumab	14 (22.2)	7 (18.4)	7 (28)	
Combination with chemotherapy (n, %)	42 (66.7)	28 (73.7)	14 (56)	0.18
Lines of treatment (median, Q1-Q3)	1 (1-2)	1 (1-2)	2 (1-2)	0.18
Pathologic features (n, %)				
TPS > 0%	57 (90.5)	36 (94.7)	21 (84)	0.20
TPS≥1%	54 (85.7)	34 (89.5)	20 (80)	0.36
CPS≥1	53 (84.1)	34 (89.5)	19 (76)	0.26
ENE	16 (25.3)	11 (28.9)	5 (20)	0.56
LVI	17 (27.0)	8 (21.1)	9 (36)	0.25
PNI	26 (41.3)	16 (42.1)	10 (40)	0.87
WPOI-5	8 (12.7)	4 (10.5)	4 (16)	0.52
P16	2 (3.2)	1 (2.6)	1 (4)	0.55

TPS: tumor proportion score, CPS: combined positive score; ENE: extranodal extension; LVI: lymphovascular invasion; PNI: perineural invasion; WPOI-5: worst pattern of invasion-5; HR: hazard ratio

Table S9B. Characteristics of GC patients receiving ICB treatment and analyzed by STAT1 Lys637 acetylation. Related to Figure 7.

	Total (n=46)	High H-score (≥166) n=19	Low H-score (<166) n=27	*P* value
Age (median, Q1-Q3)	62.6 (57.2-77.0)	65.2 (55.9-81.3)	62.9 (59.0-71.5)	0.973
Age > 65 (n. %)	24 (52.1)	10 (52.6)	14 (51.9)	0.958
Primary tumor site (n, %)				0.623
Gastroesophageal junction cancer	4 (8.7)	0	4 (14.8)	
Gastric cancer	42 (91.3	19 (100)	23 (85.2)	
Metastasis				
Metastatic before immunotherapy	30 (65.2)	10 (52.6)	20 (74.1)	0.133
Metastatic site				
Non-regional lymph nodes	9 (19.6)	3 (52.6)	6 (22.2)	0.716
Liver	7 (15.2)	3 (15.8)	4 (14.8)	0.928
Lung	4 (8.7)	1 (5.3)	3 (11.1)	0.632
Peritoneum	16 (34.8)	5 (26.3)	11 (40.7)	0.312
Others	4 (8.7)	3 (15.8)	1 (3.7)	0.292
Treatment (n, %)				
Treatment setting				
Neoadjuvant	5 (10.9)	1 (5.3)	4 (14.8)	0.387
Perioperative	4 (8.7)	2 (10.5)	2 (7.4)	0.714
Adjuvant	6 (13.0)	3 (15.8)	3 (11.1)	0.680
Locally Advanced/Metastasis	26 (56.5)	11 (57.9)	15 (78.9)	0.606
Recurrent	5 (10.9)	2 (10.5)	3 (11.1)	0.950
Operation	22 (47.8)	7 (36.8)	15 (55.6)	0.211
Types of immunotherapy				0.675
Pembrolizumab	7 (14.2)	4 (21.1)	3 (11.1)	
Nivolumab	35 (76.1)	13 (68.4)	22 (81.5)	
Durvalumab	4 (8.7)	2 (10.5)	2 (7.4)	
Combination with chemotherapy	38 (82.6)	13 (68.4)	25 (92.6)	0.051

Lines of prior systemic treatment (median, Q1-Q3)	0 (0-1)	0.5 (0-1)	0 (0-0.5)	0.694
Pathologic features (n, %)				
Poor differentiation	30 (65.2)	12 (63.2)	18 (66.7)	
Tumor cell PD-L1 expression				
CPS ≥ 1	36 (78.3)	13 (68.4)	23 (85.2)	0.175
CPS ≥ 5	28 (60.9)	11 (57.9)	17 (63.0)	0.914
Non-reported or invalid	8 (17.4)	4 (21.1)	4 (14.8)	0.490
HER2				
Positive	2 (4.3)	1 (5.3)	1 (3.7)	0.801
Negative	44 (95.7)	18 (94.7)	26 (96.3)	
EBER				
Positive	2 (4.3)	0	2 (7.4)	0.611
Negative	18 (39.1)	8 (42.1)	10 (52.6)	
Non-reported or invalid	26 (56.5)	11 (57.9)	15 (55.6)	
Microsatellite instability status				0.683
Microsatellite stable	17 (37.0)	5 (26.3)	12 (44.4)	
Microsatellite instability-high	10 (21.7)	4 (21.1)	6 (22.2)	
Non-reported or invalid	19 (41.3)	10 (52.6)	9 (33.3)	
For patients who received operation (n=22)				
Lauren's classification				0.889
Diffuse type	6 (27.3)	3 (42.9)	3 (20.0)	
Interstitial type	1 (4.5)	0	1 (6.7)	
Mixed type	9 (40.9)	2 (28.6)	7 (46.7)	
Unknown	6 (27.3)	2 (28.6)	4 (26.7)	
Lymphatic emboli	9 (40.9)	2 (28.6)	7 (31.8)	0.673
Vascular emboli	5 (22.7)	1 (14.3)	4 (26.7)	0.840

PD-L1: Programmed death-ligand 1; CPS: Combined Positive Score; HER2: Human Epidermal Growth Factor Receptor 2; EBER: Epstein–Barr virus–encoded small RNA; HR: Hazard Ratio

Table S9C. Characteristics of HCC patients receiving ICB treatment and analyzed by STAT1 Lys637 acetylation. Related to Figure 7.

Potential factors (n,%)	Total (n=39)	High H-score (≥166) n=23	Low H-score (<166) n=16	*P* value
Age (median, Q1-Q3)	64.0 (55.1-73.4)	69.2 (53.9-74.2)	63.9 (59.3-69.9)	0.789
Age > 65	18 (46.2)	12 (52.2)	6 (37.5)	0.516
Etiology				0.740
HBV hepatitis	22 (56.4)	12 (52.2)	10 (62.5)	
HCV hepatitis	2 (5.1)	1 (4.3)	1 (6.3)	
non-hepatitis	14 (35.9)	9 (39.1)	5 (31.3)	
Child–Pugh classification (n, %)				0.631
A	35 (89.7)	20 (87.0)	15 (93.8)	
B	4 (10.3)	3 (13.0)	1 (6.3)	
Barcelona Clinic liver cancer (BCLC) stage				0.307
B	11 (28.2)	9 (39.1)	6 (37.5)	
**C**	28 (71.8)	18 (78.3)	10 (62.5)	
Portal vein thrombosis	22 (56.4)	14 (60.9)	8 (50)	0.531
Metastasis				
Metastatic before immunotherapy	20 (51.3)	12 (52.2)	8 (50)	0.894
Metastatic site				
Bone	9 (23.1)	4 (17.4)	5 (31.3)	0.444
Lung	8 (20.5)	4 (17.4)	4 (25)	0.694
Peritoneum	4 (10.3)	4 (17.4)	0	0.130
Others	3 (7.7)	3 (13.0)	0	0.255
Alpha-fetoprotein ≥400 ng per milliliter	19 (48.7)	14 (60.9)	5 (31.3)	0.105
Treatment (n, %)				
Prior local treatment	26 (66.7)	14 (60.9)	12 (75)	0.495
Types of immunotherapy				0.169
Pembrolizumab	2 (5.1)	1 (4.3)	1 (6.3)	
Nivolumab	10 (25.6)	8 (34.8)	2 (12.5)	
Atezolizumab	26 (66.4)	13 (56.5)	13 (81.3)	

Durvalumab	2 (5.1)	1 (4.3)	1 (6.3)	
Lines of prior systemic treatment (median, Q1-Q3)	0 [0-0]	0 [0-1]	0 [0-0]	0.075

HBV: Hepatitis B virus, HCV: hepatitis C virus

Table S10. Determination of the cut-off value of STAT1 Lys637 acetylation. Related to Figure 7.

Test Variable	Sensitivity	1-Specificity	Youden.index
-Inf	1.000	1.000	0.000
60	1.003	0.978	0.025
90	1.000	0.958	0.042
110	0.979	0.938	0.041
113	0.960	0.938	0.022
117	0.961	0.927	0.034
118	0.942	0.927	0.015
120	0.921	0.897	0.024
124	0.922	0.886	0.037
125	0.903	0.886	0.017
128	0.903	0.876	0.027
130	0.893	0.819	0.074
138	0.894	0.808	0.086
140	0.870	0.737	0.132
149	0.871	0.726	0.145
150	0.828	0.666	0.161
155	0.829	0.655	0.174
158	0.831	0.644	0.187
159	0.831	0.602	0.228
160	0.780	0.505	0.274
161	0.781	0.494	0.287
162	0.757	0.497	0.261
163	0.759	0.485	0.274
164	0.741	0.464	0.278
165	0.741	0.443	0.297
**166**	**0.743**	**0.432**	**0.325**
168	0.725	0.420	0.305
169	0.685	0.421	0.265
170	0.636	0.333	0.302
171	0.616	0.333	0.283
173	0.612	0.326	0.286
176	0.581	0.311	0.270
177	0.572	0.305	0.267
178	0.555	0.283	0.271
180	0.478	0.200	0.279
181	0.421	0.200	0.221
182	0.401	0.200	0.202
183	0.382	0.200	0.182

Test Variable	Sensitivity	1-Specificity	Youden.index
185	0.385	0.177	0.208
186	0.366	0.177	0.189
188	0.347	0.177	0.169
190	0.239	0.111	0.128
191	0.217	0.112	0.105
194	0.218	0.091	0.127
195	0.220	0.079	0.141
198	0.192	0.073	0.119
199	0.182	0.068	0.114
200	0.144	0.058	0.086
202	0.135	0.052	0.083
203	0.135	0.042	0.093
210	0.096	0.000	0.096
211	0.077	0.000	0.077
215	0.058	0.000	0.058
220	0.039	0.000	0.039
223	0.019	0.000	0.019
236	0.000	0.000	0.000

Table S11. Cox regression analysis of risk factors for OS and PFS of HNSCC, GC and HCC patients receiving ICB. Related to Figure 7.

Table S11A. Cox regression analysis of risk factors for OS and PFS of HNSCC patients receiving ICB (n=63). Related to Figure 7.

Variables	PFS (progression free survival)				OS (overall survival)			
	Univariate		Multivariate		Univariate		Multivariate	
	HR (95% CI)	*P* value	HR (95% CI)	*P* value	HR (95% CI)	*P* value	HR (95% CI)	*P* value
Age (>65)	0.50 (0.22-1.12)	**0.092**	1 (0.97-1.02)	0.64	1.04 (0.48-2.23)	0.92		
Sex	1.32 (0.52-3.39)	1.32			1.72 (0.52-5.64)	0.37		
Primary tumor recurrence (Oral cavity vs others)	1.04 (0.52-1.78)	0.90			1.25 (0.64-2.46)	0.51		
Primary tumor recurrence (Oropharynx/larynx vs others)	0.92 (0.48-1.76)	0.81			0.74 (0.36-1.56)	0.43		
Treatment								
Types of immunotherapy	1.89 (0.84-4.28)	*0.13*			1.15 (0.50-2.64)	0.75		
Combination with chemotherapy	2.80 (1.28-6.13)	**0.01**	2.39 (1.06-5.38)	**0.007**	1.98 (0.92-4.30)	**0.082**	1.67 (0.76-3.66)	0.204
Lines of treatment	1.19 (0.95-1.49)	*0.13*			1.19 (0.90-1.59)	0.22		
Pathologic features								
TPS > 0%	0.77 (0.30-1.96)	0.58			0.69 (0.26-1.78)	0.44		
TPS≧1%	0.58 (0.27-1.25)	*0.16*			0.97 (0.75-1.27)	0.85		
CPS≧1	1.08 (0.96-1.22)	*0.18*			1.07 (0.95-1.20)	0.29		
ENE (positive)	1.24 (0.62-2.50)	0.55			1.45 (0.69-3.05)	0.33		
LVI (positive)	1.05 (0.53-2.06)	0.89			1.19 (0.40-1.75)	0.64		
PNI (positive)	1.12 (0.60-2.10)	0.71			1.00 (0.50-1.99)	1.00		
WPOI-5 (positive)	1.06 (0.44-2.52)	0.90			0.79 (0.28-2.26)	0.67		
P16 (positive vs negative)	1.55 (0.75-3.22)	0.24			30.7(0.00-1.0085E+38)	0.94		
H-score of STAT1 Lys637	2.2 (1.04-3.93)	**0.038**	1.57 (0.79-3.13)	**0.035**	2.2 (1.01-4.58)	**0.035**	1.95(0.92-4.13)	0.082

TPS: tumor proportion score, CPS: combined positive score; ENE: extranodal extension; LVI: lymphovascular invasion; PNI: perineural invasion; WPOI-5: worst pattern of invasion-5; HR: hazard ratio

Table S11B. Cox regression analysis of risk factors for PFS and OS of GC patients receiving ICB (n=46). Related to Figure 7.

Variables	PFS (progression free survival)				OS (overall survival)			
	Univariate		Multivariate		Univariate		Multivariate	
	HR (95% CI)	*P* value	HR (95% CI)	*P* value	HR (95% CI)	*P* value	HR (95% CI)	*P* value
Age (>65)	0.96 (0.04-2.11)	0.918	‘		0.90 (0.30-2.68)	0.848		
Primary tumor site (EGJ vs gastric)	0.50 (0.12-2.17)	0.358			0.04 (0.0-67.42)	0.396		
Metastatic site (peritoneum vs others)	1.08 (0.45-2.57)	0.866			2.32 (0.70-7.77)	0.17		
(non-regional lymph nodes vs others)	0.96 (0.40-2.31)	0.927			1.64 (0.44-6.11)	0.458		
Treatment								
Treatment settings (Metastases/advanced vs others)	1.91 (0.77-4.76)	0.163			37.7 (0.28-507.5	0.147		
Operation	0.31 (0.13-0.71)	**0.006***	0.29 (0.13-0.68)	**0.004***	0.02 (0.00-1.13)	**0.057**[#]		
Types of immunotherapies (Durvalumab vs others)	0.57 (0.08-4.22)	0.58			0.82 (0.22-2.99)	0.762		
Combination with chemotherapy	2.88 (0.86-9.67)	**0.088**			1.70 (0.37-7.73)	0.494		
Prior chemotherapy to ICB	0.45 (0.19-1.08)	**0.073**			0.20 (0.04-0.9)	**0.036***		
Pathologic features								
Poor differentiation	1.27 (0.47-3.46)	0.641			1.21 (0.33-4.43)	0.779		
CPS ≧ 1	0.70 (0.30-1.61)	0.396			0.54 (0.07-4.23)	0.555		
CPS ≧ 5	0.86 (0.48-1.53)	0.610			0.79 (0.33-4.88)	0.728		
HER2 (positive)	1.37 (0.31-6.10)	0.680			0.046 (0-632.6)	0.611		
EBER (positive)	1.32 (0.86-2.04)	0.208			1.41 (0.74-2.67)	0.299		
MSS	2.51 (1.29-4.87)	**0.007***	2.57 (1.32-5.00)	**0.005***	3.41 (1.28-9.07)	**0.014***		
Lauren's classification (diffuse vs others)	3.11 (1.18-8.25)	**0.022***						
Lymphatic emboli	1.36 (0.27-6.99)	0.710						
Vascular emboli	2.31 (0.27-20.0)	0.447					3.56 (1.39-9.13)	**0.008***
H-score of STAT1 Lys637	1.42 (0.65-3.07)	0.378			3.07 (1.00-9.46)	**0.05**	2.67 (0.86-8.29)	0.09

PD-L1: Programmed death-ligand 1; CPS: Combined Positive Score; HER2: Human Epidermal Growth Factor Receptor 2; MSS: Microsatellite stability; EBER: Epstein–Barr virus–

encoded small RNA; HR: Hazard Ratio

*P < 0.05

† Lauren's classification, lymphatic emboli and vascular emboli were only evaluated in PFS, due to patients with available data were all alive (no death events)

#The hazard ratio for operation could not be reliably estimated in the multivariate model due to extremely low event rates in one subgroup, resulting in unstable model convergence and inflated confidence intervals

Table S11C. Cox regression analysis of risk factors for PFS and OS of HCC patients receiving ICB (n=39). Related to Figure 7.

Variables	PFS (progression free survival)				OS (overall survival)			
	Univariate		Multivariate		Univariate		Multivariate	
	HR (95% CI)	*P* value	HR (95% CI)	*P* value	HR (95% CI)	*P* value	HR (95% CI)	*P* value
Age (>65)	0.76 (0.34-1.71)	0.508			1.79 (0.52-6.10)	0.355		
Etiology (Viral vs non-viral)	1.358 (0.58-3.18)	0.482			3.18 (0.69-14.78)	0.140		
Child–Pugh classification (B vs A)	1.84 (0.53-6.34)	0.334			4.35 (0.88-21.47)	***0.071***		
Barcelona Clinic liver cancer (BCLC) stage (C vs B)	1.29 (0.51-3.28)	0.596			2.89 (0.63-13.35)	0.174		
Alpha-fetoprotein ≥400 ng per milliliter	1.19 (0.53-2.65)	0.675			2.70 (0.79-9.18)	0.111		
Portal vein thrombosis	1.03 (0.43-2.18)	0.942			2.77 (0.79-9.64)	0.113		
**Metastasis**								
Metastatic before immunotherapy	1.36 (0.58-3.18)	0.482			1.52 (0.21-2.78)	0.474		
Metastatic site								
Lung	1.28 (0.5-3.23)	0.599			2.82 (0.58-13.89)	0.199		
Peritoneum	1.72 (0.14-2.49)	0.465			0.90 (0.11-7.22)	0.921		
Others	2.52 (0.74-8.62)	0.140	2.39 (0.69-8.24)	0.168	5.29 (1.0-26.18)	**0.037***	5.73 (1.07-30.54)	**0.041***
Treatment								
Prior local treatment	0.53 (0.22-1.25)	0.145	0.54 (0.23-1.29)	0.167	0.138 (0.03-0.57)	**0.006***	0.13 (0.03-0.56)	**0.006***
Types of immunotherapy								
Atezolizumab vs others	0.85 (0.36-1.99)	0.704						
H-score of STAT1 Lys637	1.26 (0.55-2.88)	0.587			6.39 (0.82-50.00)	**0.077**		

HBV: Hepatitis B virus, HCV: hepatitis C virus; HR: Hazard Ratio, CI: Confidence Interval

Table S12. Treatment response to ICB-based regimen according to H-score of STAT1 Lys637. Related to Figure 7.

**Cancer type**	**GC**	**HCC (RECIST 1.1)**	**HCC (mRECIST)**	**HNSCC**
N (H score ≥ 166)	19	23	23	38
N (H score < 166)	27	16	16	25
**Treatment Response, n(%)**				
CR (H score ≥ 166)	0	1 (4.3)	5 (21.7)	1 (3.6)
CR (H score < 166)	3 (11.1)	0	0	4 (16.0)
PR (H score ≥ 166)	11 (57.9)	8 (42.1)	6 (26.1)	8 (21.1)
PR (H score < 166)	9 (33.3)	8 (50.0)	9 (56.3)	14 (56.0)
SD (H score ≥ 166)	4 (21.1)	5 (21.7)	3 (13.0)	10 (26.3)
SD (H score < 166)	10 (37.9)	4 (25.0)	3 (18.8)	5 (20.0)
PD (H score ≥ 166)	4 (21.1)	9 (39.1)	9 (39.1)	19 (50.0)
PD (H score < 166)	5 (18.5)	4 (25.0)	4 (25.0)	2 (8.0)
**Clinical Outcomes, % (95% CI)**				
DCR (H score ≥ 166)	78.9 (60.6-97.2)	60.9 (40.9-80.8)	60.9 (40.9-80.8)	50.0 (27.5-72.5)
DCR (H score < 166)	81.5 (66.8-96.1)	75 (53.8-96.2)	75 (53.8-96.2)	92.0 (80.9-100)
P value	0.831	0.495	0.495	0.001*
ORR (H score ≥ 166)	57.9 (35.7-80.1)	39.1 (19.2-59.1)	47.8 (27.4-68.2)	23.7 (4.5-42.8)
ORR (H score < 166)	44.4 (25.7-63.2)	50 (25.5-74.5)	56.3 (31.9-80.6)	72.0 (53.7-90.4)
P value	0.55	0.342	0.748	<0.0001*

GC: gastric cancer, HCC: hepatocellular carcinoma, HNSCC: head and neck squamous cell carcinoma, CR: complete response, PR: partial response, SD: stable disease, PD: progressive disease, DCR: disease control rate, ORR: objective response rate, mRECIST: modified Response Evaluation Criteria in Solid Tumors Version, RECIST: Response Evaluation Criteria in Solid Tumors Version

Table S13. ELISA for validation of specificity of antibody for Lys637-acetylated STAT1. Related to STAR Methods.

Polyclonal antibody

Anti- ac-STAT1-Lys637 hot peptide:											
Mean OD value:											
Dilute	No.1	No.2	No.3	No.4	No.5	No.6	No.7	No.8	No.9	No.10	(-) sera-1
500x	2.648	2.009	1.943	2.357	1.554	2.94	1.457	3.103	2.613	2.941	0.069
2500x	1.496	0.925	0.768	1.238	0.614	1.759	0.635	2.081	1.45	1.912	0.055
12500x	0.531	0.301	0.239	0.438	0.19	0.704	0.2	0.907	0.509	0.804	0.047
62500x	0.162	0.104	0.088	0.14	0.077	0.22	0.084	0.292	0.158	0.249	0.048

Blank=0.046

Anti-STAT1-Lys637 cold peptide:											
Mean OD value:											
Dilute	No.1	No.2	No.3	No.4	No.5	No.6	No.7	No.8	No.9	No.10	(-) sera-1
500x	1.289	2.11	2.237	2.312	1.407	2.418	1.174	2.144	2.543	2.8	0.065
2500x	0.447	0.985	1.043	1.234	0.516	1.131	0.514	0.976	1.367	1.782	0.051
12500x	0.139	0.315	0.335	0.447	0.163	0.384	0.169	0.303	0.464	0.75	0.047
62500x	0.066	0.108	0.114	0.136	0.07	0.128	0.075	0.105	0.155	0.242	0.048

Monoclonal antibody

Anti- ac-STAT1-Lys637 hot peptide:								
Mean OD value:								
Dilute	2E2(IgM)	5B2(IgM)	5B11(IgM)	7C7(IgM)	8C1(IgM)	9D4(IgM)	10F8(IgM)	2E11(IgM)
1x	1.387	3.691	3.462	3.714	1.911	1.276	3.597	2.619
10x	2.212	3.089	2.95	3.129	3.323	1.729	3.567	2.989
100x	0.703	3.375	3.451	2.073	2.91	0.582	1.86	2.125

Blank=0.047

Anti-STAT1-Lys637 cold peptide:								
Mean OD value:								
Dilute	2E2(IgM)	5B2(IgM)	5B11(IgM)	7C7(IgM)	8C1(IgM)	9D4(IgM)	10F8(IgM)	2E11(IgM)
1x	1.632	3.556	3.391	3.498	1.572	1.325	3.478	2.575
10x	2.461	3.359	3.073	3.364	3.342	1.898	3.524	2.952
100x	0.821	3.482	3.633	1.846	3.075	0.769	1.901	1.977

Blank=0.048

Table S14. qRT-PCR primers list. Related to STAR Methods.

gene name	gene id	Forward (5'-3')	Reverse (5'-3')
GAPDH	ENSG00000111640	CGCTCTCTGCTCCTCCTGTTC	TTGACTCCGACCTTCACCTTCC
STAT1	ENSG00000115415	CCTGCTGCGGTTCAGTGA	GGTTCAACCGCATGGAAGTC
IRF1	ENSG00000125347	GAGGAGGTGAAAGACCAGAGCA	TAGCATCTCGGCTGGACTTCGA
CD274	ENSG00000120217	TCAATGCCCCATACAACAAA	TGCTTGTCCAGATGACTTCG
CXCL9	ENSG00000138755	CGTGGTAAAACACTTGCGGATATT	CAATCATGCTTCCACTAACCGACT
CXCL10	ENSG00000169245	GCCATTCTGATTTGCTGCCTT	GCACTGCATCGATTTTGCTC
CXCL 11	ENSG00000169248	GATGCCTAAATCCCAAATCGAA	GGCAGTGGAATTCTGATTGTCA
HLA-A	ENSG00000206503	AGATACACCTGCCATGTGCAGC	GATCACAGCTCCAAGGAGAACC
HLA-B	ENSG00000234745	CTGCTGTGATGTGTAGGAGGAAG	GCTGTGAGAGACACATCAGAGC
HLA-C	ENSG00000204525	GGAGACACAGAAGTACAAGCGC	ACATCCTCTGGAGGGTGTGAGA
TAP1	ENSG00000168394	CGTCCACCCTGAGTGATTCT	GACACTGATCCCCAGAGCAT
TAP2	ENSG00000204267	AGGAGGCTGCTTCACCTACA	TGAGTTCAGCTCCCCTGTCT
TAPBP	ENSG00000231925	GAGCCTGTTCTCATCACCATGG	GTAGGCAAAGCTCAAGTCCAGC
B2M	ENSG00000166710	GTGCTCGCGCTACTCTCTCT	TCAATGTCGGATGGATGAAA
PSMB9	ENSG00000240065	ATGGAACCCTGGGAGGAATG	GAGCAATAGCGTCTGTGGTG
PSME1	ENSG00000092010	TGCCTGATCCAGTCAAGGAG	CACAGGGAGGACCTTTGTCT
PSME2	ENSG00000100911	GAGAAAGTCCTGTCCCTGCT	CCAGCACCTTCTCCTGGATT
OAS1	ENSG00000089127	TTTGATGCCCTGGGTCAGTT	AGTTCTGTGAAGCAGGTGGA
EIF2AK2	ENSG00000055332	GCACCCAGATTTGACCTTCC	CTTGGCCAAATCCACCTGAG
ADAR	ENSG00000160710	ATATCAGCACTGCTCCGTGT	GGATTGTGCCTTCTCCGTTC
IFITM1	ENSG00000185885	TCTTCTTGAACTGGTGCTGTC	GTCGCGAACCATCTTCCTGT
IFITM2	ENSG00000185201	CCTTGACCTGTATTCCACT	GCCATTGTAGAAAAGCGT
IFITM3	ENSG00000142089	TCCCACGTACTCCAACTTCCA	AGCACCAGAAACACGTGCACT
ISG15	ENSG00000187608	CGCAGATCACCCAGAAGATCG	TTCGTCGCATTTGTCCACCA
TRIM5	ENSG00000132256	CTGGAGATGCTGAGGCAGAAGC	GTCCAGGATGTCTCTCAGTTGC
IFNGR1	ENSG00000027697	AGTGCTTAGCCTGGTATTCATCTG	GGCTGGTATGACGTGATGAGTG
IFNGR2	ENSG00000159128	CTCCATTCTGCCTGGGTGACAA	CGTGGAGGTATCAGCGATGTCA
JAK1	ENSG00000162434	GAGACAGGTCTCCCACAAACAC	GTGGTAAGGACATCGCTTTTCCG
JAK2	ENSG00000096968	CCAGATGGAAACTGTTCGCTCAG	GAGGTTGGTACATCAGAAACACC
CD34	ENSG00000174059	TAGCCTTGCAACATCTCCCA	TTAAACTCCGCACAGCTGGA
FUT4	ENSG00000196371	CAGAAGACGGTCTGACTTGC	GCTTCATCAAGGCCACAGAG
MADCAM1	ENSG00000099866	CCAACAGGCTCGTCCAAAC	CAGAGGTGATAGGTGGGCAA
NTAN1	ENSG00000157045	GAACCGACACCAAAGCTGAG	TTTGTGACAACTGCCTGTCG
CD80	ENSG00000121594	AGTTAGAAGGGGAAATGTCGC	TCAGGGTAAGACTCCACTTCTG
CD86	ENSG00000114013	ATTCTGAACTGTCAGTGCTTGC	CTTCTTAGGTTCTGGGTAACCG
LGALS9	ENSG00000168961	CTTTCATCACCACCATTCTG	ATGTGGAACCTCTGAGCACTG
TNFRSF14	ENSG00000157873	TCT CTG CTG CCA GAC A	GCC ACA GCA GAA CAG A

CD276	ENSG00000103855	GTGGGGCTGTCTGTCTGTCTCAT	GCTGTCAGAGTGTTTCAGAGGCT
VTCN1	ENSG00000134258	AGGGAGTGGAGGAGGATACAG	GCAGCAGCCAAAGAGACAG
ATP2C1	ENSG00000017260	GAAGGCTGCAGACATTGGAG	TGCTGCTATACTCGTGCTCA
CXCL1	ENSG00000163739	AGGGAATTCACCCCAAGAAC	CACCAGTGAGCTTCCTCCTC
CXCL3	ENSG00000163734	AGCACCAACTGACAGGAGAG	GTCCTTTCCAGCTGTCCCTA
EGFR	ENSG00000146648	CCAAACTGCACCTACGGATG	ACGGGATCTTAGGCCCATTC
IL11	ENSG00000095752	CCACCACACCTGACCCATAG	TTGACCTTGCAGCTTTGTCC
IL1B	ENSG00000125538	AGCTGAGGAAGATGCTGGTT	GTGATCGTACAGGTGCATCG
IL6	ENSG00000136244	CAGGAGCCCAGCTATGAACT	CAGGGAGAAGGCAACTGGA
IRAK2	ENSG00000134070	CAACTTGTGGACCTCCTGTG	CCGGTTTCCAGTTCAGGATG
NAMPT	ENSG00000105835	GCAGAAGCCGAGTTCAACAT	CTTTGCTTGTGTTGGGTGGA
SLC7A2	ENSG00000003989	TGCAGACACTTGGGTCAGAT	CCCTCCAGGCTGTGTCTAAT
SOCS3	ENSG00000184557	CGCCTTAAATGCTCCCTGTC	CCCATCCAGGCTGAGTATGT
IL12A	ENSG00000168811	AGTGGAGGCCTGTTTACCAT	AAGGCACAGGGCCATCATAA
ST8SIA4	ENSG00000113532	CAGAGGTTACTGGCTGACCA	GGGCCAGAATCCATACAGGT
Smurf1	ENSG00000198742	AGTCCTCAGACACGAACTGTCG	GTCGCATCTTCATTATCTGGCGG
PDLIM2	ENSG00000120913	GAGAAGTGCAGTACCAGCATCG	GCATCTTCAGGTTCAGCCCACA
